# Advances in photocatalytic degradation of organic pollutants in wastewaters: harnessing the power of phthalocyanines and phthalocyanine-containing materials

**DOI:** 10.1039/d3ra06598g

**Published:** 2023-11-20

**Authors:** Sara R. D. Gamelas, João P. C. Tomé, Augusto C. Tomé, Leandro M. O. Lourenço

**Affiliations:** a LAQV–REQUIMTE, Department of Chemistry, University of Aveiro 3810–193 Aveiro Portugal actome@ua.pt leandrolourenco@ua.pt; b Centro de Química Estrutural, Institute of Molecular Sciences, Departamento de Engenharia Química, Instituto Superior Técnico, Universidade de Lisboa 1049–001 Lisboa Portugal jtome@tecnico.ulisboa.pt

## Abstract

Access to clean water is increasingly challenging worldwide due to human activities and climate change. Wastewater treatment and utilization offer a promising solution by reducing the reliance on pure underground water. However, it is crucial to develop efficient and sustainable methods for wastewater purification. Among the emerging wastewater treatment strategies, photocatalysis has gained significant attention for decomposing organic pollutants in water, especially when combined with sunlight and a recoverable photocatalyst. Heterogeneous photocatalysts have distinct advantages, as they can be recovered and reused without significant loss of activity over multiple cycles. Phthalocyanine dyes, with their exceptional photophysical properties, are particularly valuable for homogeneous and heterogeneous photocatalysis. By immobilizing these photosensitizers in various supports, hybrid materials extend their light absorption into the visible spectrum, complementing most supports' limited UV light absorption. The novelty and research importance of this review stems from its discussion of the multifaceted approach to treating contaminated wastewater with phthalocyanines and materials containing phthalocyanines. It highlights key aspects of each study, including photocatalytic efficiency, recyclability characteristics, investigation of the generation of oxygen species responsible for degradation, identification of the major degradation byproducts for each pollutant, and others. Moreover, the review includes tables that illustrate and compare the various phthalocyanines and supporting materials employed in each study for pollutant degradation. Additionally, almost all photocatalysts mentioned in this review could degrade at least 5% of the pollutant, and more than 50 photocatalysts showed photocatalytic rates above 50%. When immobilized in some support, the synergistic effect of the phthalocyanine was visible in the photocatalytic rate of the studied pollutant. However, when performing these types of works, it is necessary to understand the degradation products of each pollutant and their relative toxicities. Along with this, recyclability and stability studies are also necessary. Despite the good results presented in this review, some of the works lack those studies. Moreover, none of the works mentions any study in wastewater.

## Abstract 2

Access to clean water is increasingly challenging worldwide due to human activities and climate change.

## Graphical abstract

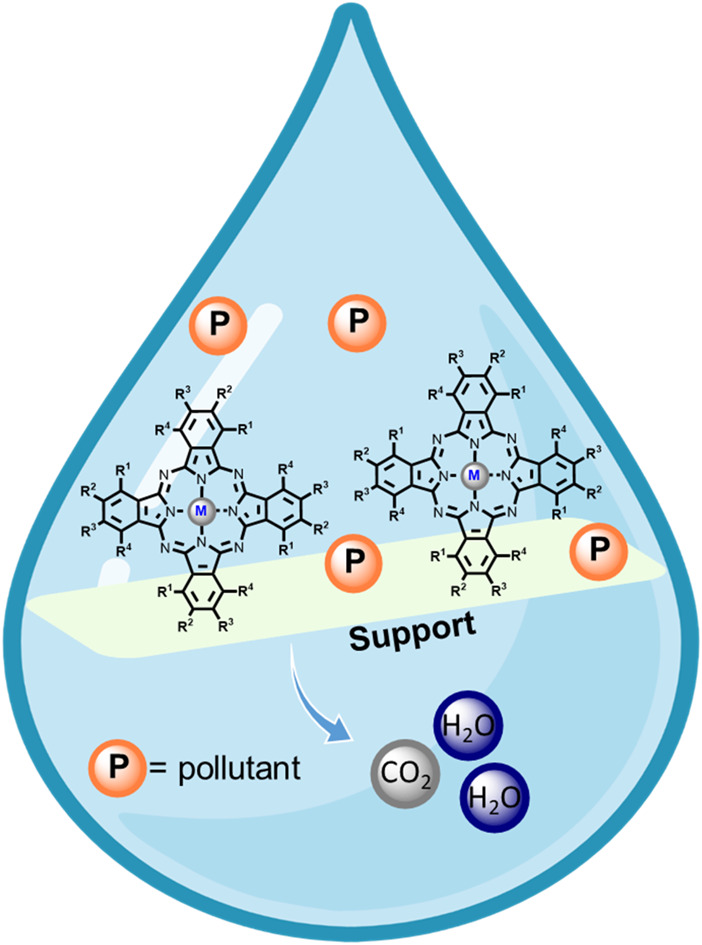

## 1. Introduction

Clean water is an essential requirement for human health. The principal drinking water sources include groundwater, lakes, canals, rainwater, and sea water.^1,2^ Recently, there has been a concern worldwide about providing sustainable, pure water due to the continuous increase in consumption, population growth, and industrial activity.^3,4^ The demand for freshwater resources for domestic or industrial use has led to scarcity.^3,5^ This demand is expected to increase by nearly one-third in 2050, according to the United Nations' World Water Development Report 2018.^6^ Therefore, to meet the increased water requirement, the scientific community is developing efficient wastewater treatment methods.^7^ Conventional wastewater treatments consist of a combination of physical, chemical, and biological processes to remove organic matter and, in some cases, inorganic nutrients.^8^ The methods for water purification are divided into six processes: adsorption, biotechnological, magnetic, membrane, and (photo)catalytic.^1^ Concerning the photocatalytic processes, the disadvantages of the ‘traditional’ homogenous photocatalysts, namely their recovery and reuse, were overcome by the advent of heterogeneous photocatalysts and allowed the implementation of large-scale photocatalytic transformations. However, homogenous photocatalysts are still useful for finding potentially promising molecules for heterogeneous photocatalysis.^9,10^

In the past decade, nanomaterials such as SiO_2_, ZnWO_4_, ZnO, fibrous materials, ferritic nanomaterials, and carbon-based and TiO_2_ nanomaterials have been reported in UV-visible light photocatalysis.^7,11^ Among them, the most studied and used are the TiO_2_ nanomaterials, discovered over three decades ago. They showed great photocatalytic activity, hydrophobicity, long-term stability, lower toxicity and costs (compared with other nanomaterials), and self-cleaning ability.^9,10^ To apply heterogeneous photocatalysis to wastewater treatment, the cost of the process should be minimal. Thus, recyclability presents a vital feature for a photocatalyst.^10,12^

There is an urge to find alternatives to improve the photocatalytic performance of materials frequently used as photocatalysts. For example, some materials only absorb UV light, representing *ca*. 5% of the solar spectrum. For example, modifying these materials with dyes can improve their photocatalytic efficiency under solar light.^10,13^ In fact, the sensitization of the photocatalyst induces faster destruction of the organic pollutants during the photocatalytic activity.^7,10,14^

**Fig. 1:**
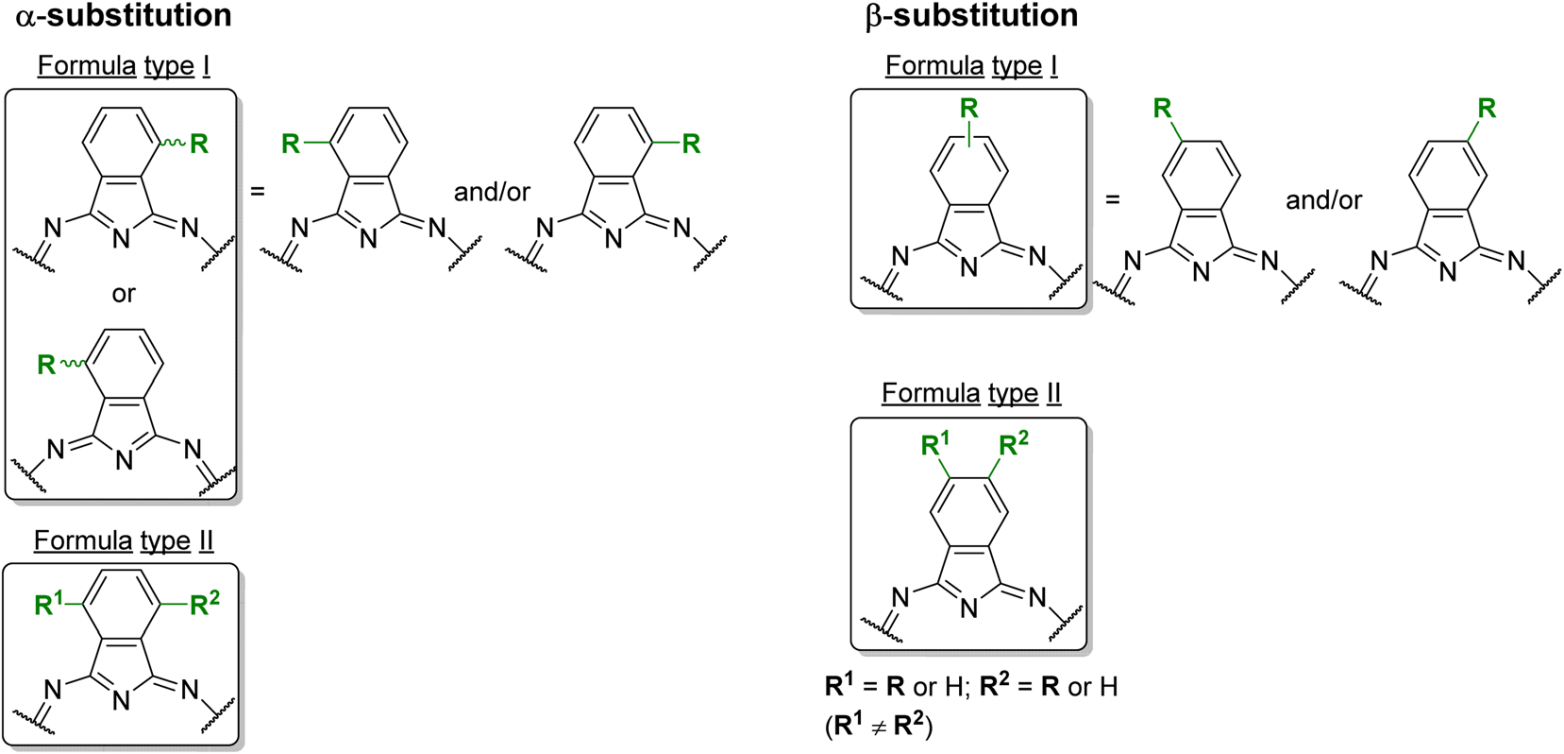
Condensed and simplified structural chemical formulae used for phthalocyanine having an α- or β-substitution patterns.

Phthalocyanines (Pcs) are dyes that can be photoactivated by visible light to promote the degradation of organic materials.^15^ Regarding chemical structure, Pcs are synthetic aromatic compounds consisting of four iminoisoindoline units with 18 delocalized π-electrons and display excellent absorption in the visible and near-infrared region of the electronic spectrum.^15–19^ Usually, the synthesis of a phthalocyanine from a mono-substituted phthalonitrile affords a mixture of regioisomers due to the variation of the peripheral position of the R groups (Fig. 1). Similarly, the tetramerization of a non-symmetrical disubstituted phthalonitrile (R^1^ ≠ R^2^) also gives a mixture of regioisomeric phthalocyanines. Given that, in this paper there are symmetrical and non-symmetrical tetra- and octasubstituted phthalocyanines with various regioisomers that are represented by the condensed structural formulae notation “Formula type I′′ and “Formula type II” (Fig. 1). So, the type I notation, despite not being formal, is often preferred to type II due to its high level of clarity, and it will be used in this review when needed.

Moreover, Pcs present great structural flexibility and particular properties that can be explored in several applications, including catalysts,^20,21^ sensors,^22–24^ solar cells,^18,25–28^ photosensitizers in biological targets,^16,17,29,30^ and, more importantly, as photocatalysts.^15,24,31,32^ When supported in a semiconductor, under visible light irradiation, Pcs are excited and inject electrons into the semiconductor's conduction band to initiate the photocatalytic process.

This review focuses on the photocatalytic degradation of organic pollutants typically found in wastewater by using several phthalocyanines as catalysts. There are several highly cited reviews in photocatalysis,^15,32,33^ but an up-to-date review is needed. A comprehensive review of the published works in this field during the last 17 years is presented herein. The pollutants mentioned in this review are divided into five main sections: phenol and phenol derivatives, organic dyes, agrochemicals, pharmaceuticals, and other pollutants. The novelty and research significance of this review are based on mentioning the multidisciplinary treatment of pollutant wastewater using phthalocyanines and phthalocyanine-containing materials, evidencing the main details of each work when these parameters are determined and provided in the multidisciplinary reports, such as the: (i) photocatalytic rates, (ii) recyclability properties, (iii) study of the main generated oxygen species responsible for the degradation, (iv) the main degradation products of each pollutant, and (v) among others. However, there is a lack of some of these parameters in several reports. In this review, it is also provided some tables to show and compare the different phthalocyanines and supports used in each work for pollutant degradation. At the end of the review, some key insights and future perspectives will be presented to inspire future research.

## 2. Mechanism of the photocatalytic degradation of organic pollutants

Among the various methods to remove the organic pollutants in wastewater, photooxidation is the most used. It involves the *in situ* generation of reactive oxygen species (ROS) that react with the pollutants, leading to their oxidation and, preferably, decomposition.^31^

**Fig. 2:**
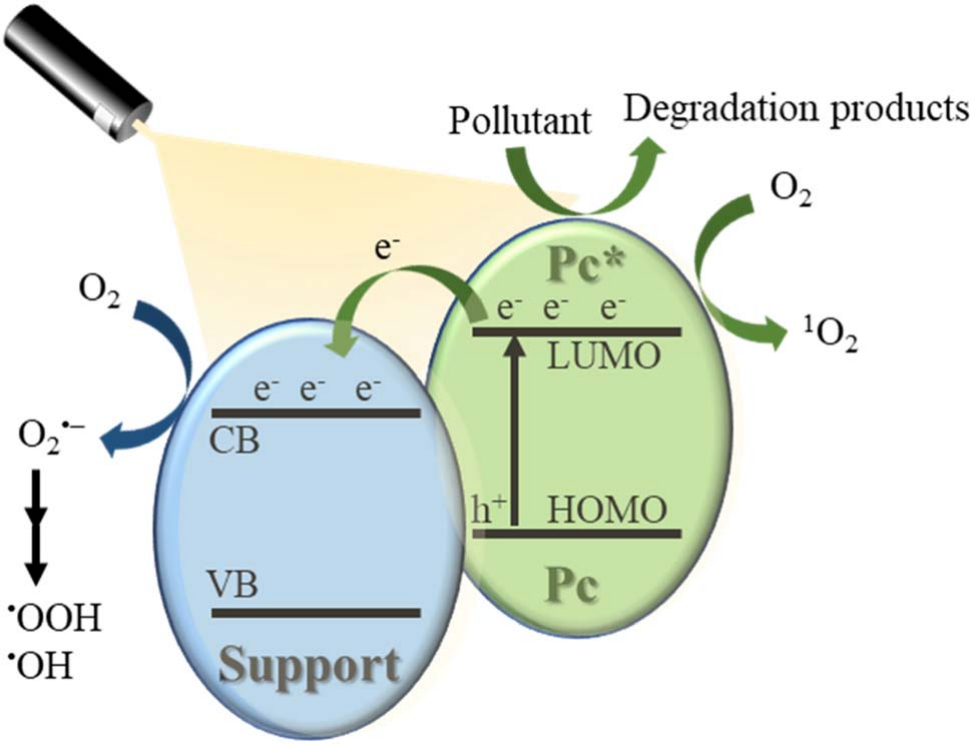
Proposed mechanism for the Pc mediated photodegradation of wastewater pollutants under visible light irradiation.

Under visible light irradiation, (metallo)phthalocyanines (MPc, M = 2H or metal ion) in a support system are excited and transfer electrons to the conduction band of the support (Fig. 2). The conduction band mediates the electron flow from the MPc to the electron acceptors on the support. It is important to highlight that the electron transfer between MPc and the conduction band of the support must be faster than the relaxation to the ground state.^7,34^ On the other hand, the excited phthalocyanine (MPc*) can act as a sensitizing oxidant (reacting directly with the pollutant) or transfer its energy to molecular oxygen to form ROS. Moreover, in the presence of water, which can act as an electron donor, the oxidized MPc can be regenerated quickly and, after a series of charge transfer reactions, superoxide radical anions (O_2_˙^−^), hydroperoxyl radicals 
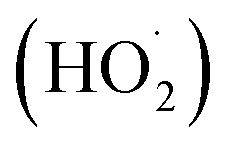
, and hydroxyl radicals (˙OH) are produced as powerful oxidizing species.^35–37^ In some studies, H_2_O_2_ is used to increase the amount of OH˙ and, thus, enhance the photocatalytic activity.^14,31,38^ The final step in the photocatalytic reaction is the relaxation of the photocatalyst to the ground state; a new catalytic cycle is then started.^7,9,10,14,33^ In the case of solar irradiation, UV light can also excite the support, increasing the formation of ROS.^7^

## 3. Photocatalytic degradation of organic pollutants

Water contaminants such as phenolic compounds, dyes, agrochemicals, pharmaceuticals, *etc.*, are hazardous to humans and harmful to the environment. Many of these substances are resistant to natural degradation processes. These contaminants reach natural waters through domestic and industrial activities in a continuous way, and that is attracting global concern. Even in localised contaminant sources, like industrial effluents, their elimination by conventional methods (chemical precipitation, filtration, electro-deposition, ion-exchange adsorption, and membrane systems) can be either slow or difficult.^39^ For the organic contaminants, photooxidation is a promising alternative to those methods.

Due to structural differences, each contaminant type raises specific degradation problems. Therefore, the results concerning the photooxidation/degradation of organic pollutants are discussed here by contaminant families, aiming to highlight the successes and drawbacks of the method for each contaminant type. It is important to mention that complete mineralization of the pollutants should be achieved in an appropriate period. The conversion of a pollutant into another compound, which could also be toxic, should be avoided.^40^ Therefore, each pollutant's degradation should be studied to evaluate if complete mineralization was achieved. Unfortunately, the degradation products of the pollutants are not mentioned in many of the studies of published articles. Moreover, many published works also do not mention recyclability studies. The recyclability of the composites used as photocatalysts is also essential when considering their viability in water and wastewater treatment.^41^

### 3.1. Phenol and phenol derivatives

The first family of contaminants to be discussed is the phenolic compounds. These are aromatic compounds with one or more hydroxyl groups linked to the aromatic ring(s). These compounds are found in the wastewater of several industries like petroleum refineries, chemical synthesis, plastics, dyes, detergents, and textiles.^42^ The appearance of phenolic compounds could also arise in the aquatic environment from natural sources, namely through algal secretion, hydrolysable tannins, and flavonoids. However, the most hazardous ones are phenol derivatives like chlorophenols, nitrophenols, bisphenol A (BPA), naphthol, catechol, *etc.*^39^ The presence of phenolic pollutants and their metabolites in living cells can cause mutagenicity, carcinogenicity, and endocrine-disrupting chemicals.

For this reason, they are considered human health and environmental hazards.^43^ The elimination of these derivatives is sometimes incomplete, so finding promising alternatives for its removal from wastewater becomes essential. The following sections will mention the degradation of these pollutants using several phthalocyanines. Also, in the following sections, studies regarding the degradation of chlorophenols using several phthalocyanines will be reported.

#### 3.1.1. Phenol

**Fig. 3:**
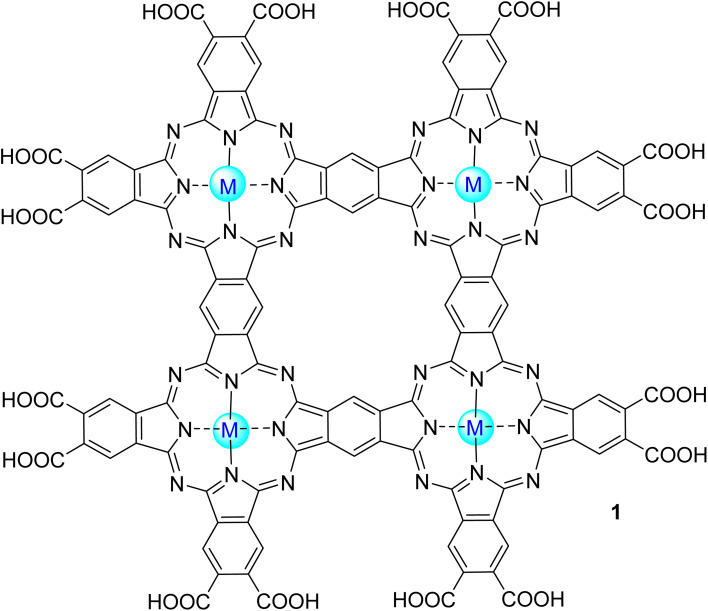
Polynuclear phthalocyanine complexes M1.

Phenol is an organic aromatic compound with a hydroxyl group linked to a phenyl ring. This can exist in the environment either naturally or chemically. In Nature, it appears as a part of coal and creosote, decomposing organic materials, and a by-product of plant metabolism. Chemically, it can be produced through the oxidation process of toluene. The presence of a high concentration of phenol in wastewater can lead to a carcinogenic problem. It can also lead to chlorine in water and form chlorophenols, which are also toxic to organisms.^44^ For this reason, it becomes important to study its elimination. So, Iliev and co-workers^45^ studied the photodegradation of phenol in water under visible light irradiation (*λ* > 400 nm) using Zn(ii), Co(ii), and Al(iii) mononuclear phthalocyanines and the water-soluble polynuclear metallophthalocyanine complexes Zn1 and Al1 (Fig. 3). In an alkaline medium (pH = 13), achieving the best degradation rate was possible using the Zn1 (*r* = 128.80 min^−1^). The activity of this catalyst could be increased to *r* = 152.44 min^−1^ by adding bulky cations, like tetrabutylammonium, that decrease the aggregation of the polynuclear Zn1 and, consequently, increase the ^1^O_2_ quantum yield (Table 1).

**Table 1:** Photophysical parameters of the derivatives 1, 2, 3, and 18 that were used for phenol photodegradation^45–50^

MPc	Support	Light	Irradiation time (min)	Half-life time (*r*, min^−1^)	Efficiency (rate or %)	Recycle	Ref.
Zn1	—	Visible light (*λ* > 400 nm)	—	152.44	—	—	45
Zn2				1324.30			
Al1				39.21			
Al2				17.13			
Co1				0.12			
Pd3	FDU-15		420	—	61%^a^^,^^d^		46
					69%^b^^,^^d^		
			360		98%^c^^,^^d^		
					56%^a^		
Al3	TiO_2_		590		90%		47
H_2_4	TiO_2_		600		1.5^e^		48
Fe4	Graphene		180		70%	4 cycles (10% loss)	49
	AlO_3_				—	—	
	CNTs						
Cu4	Surfactant modified bentonite		240		20%		50
Co4					30%		
AlCl4					70%		

a pH 11.

b pH 5, and.

c pH 7.

d With H_2_O_2_.

e mol CO_2_ per mol substrate, light intensity at 38 mW cm^−2^.

**Fig. 4:**
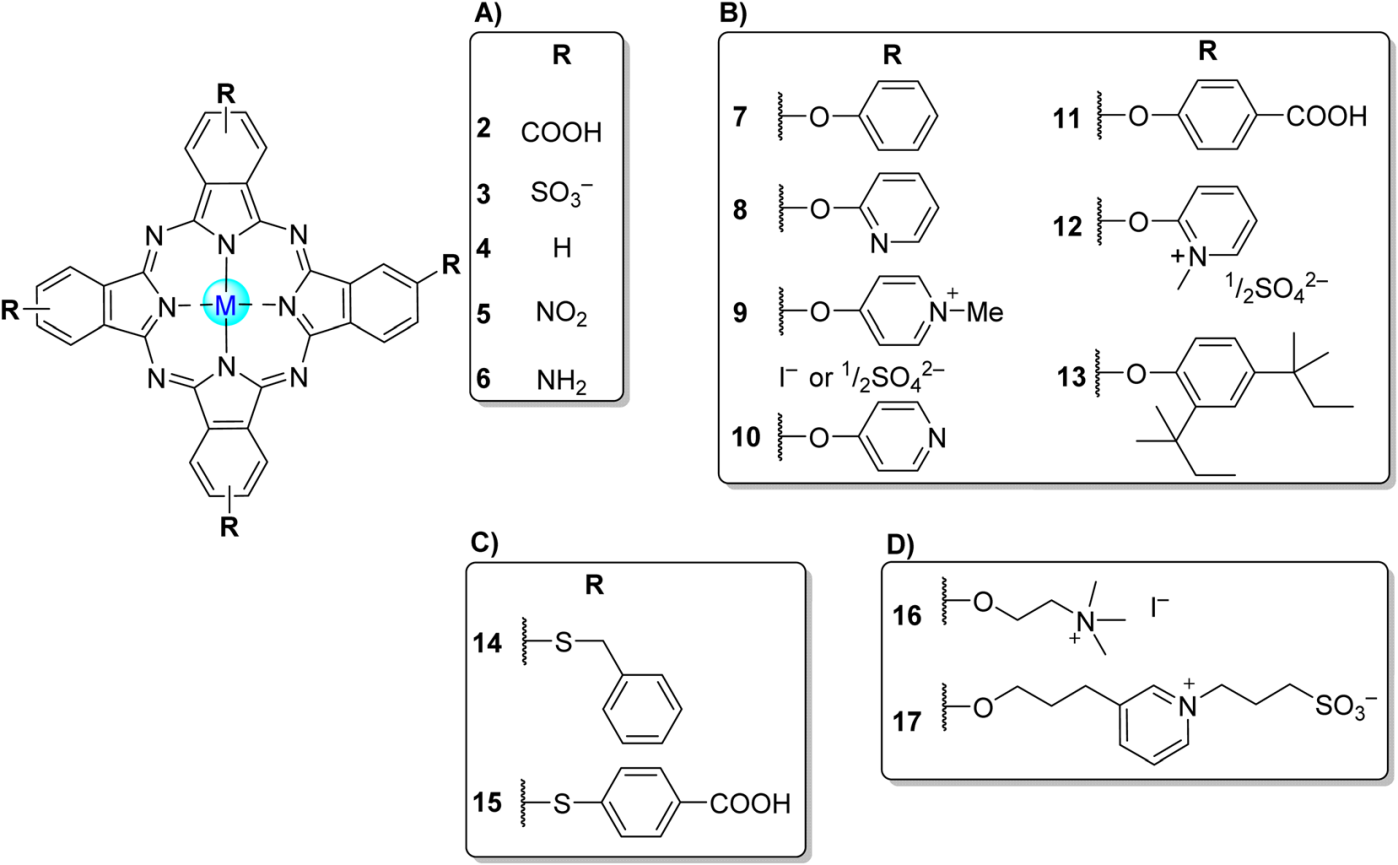
Symmetrical tetra-β-substituted phthalocyanines (A) 2–6,^34,45–96^ (B) 7–13,^84,85,97–100^ (C) 14,^50,101^15^35,101^ and (D) 16,^56,85^17.^102^

**Fig. 5:**
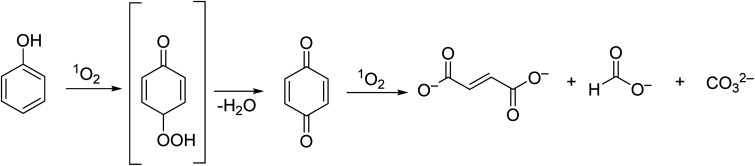
Degradation products of phenol, reported by Iliev and co-workers.^45^

Under the same conditions, the phenol degradation rate was lower using the mononuclear Zn2 (Fig. 4) (*r* = 27.0 min^−1^).^45^ When compared with Al2Cl (Fig. 4) (*r* = 17.13 min^−1^), the Al1Cl and Co1 (Fig. 3) exhibited higher photocatalytic activities (*r* = 39.21 and 0.12 min^−1^, respectively). The authors confirmed that ^1^O_2_ is the main ROS involved in the degradation process of phenol. The oxidation product, 1,4-benzoquinone, could be further degraded into fumarate and maleate at an alkaline pH (pH = 13, Fig. 5). No assays regarding the recyclability and photostability of the photocatalyst were performed in this study.

Wu, Xing, and co-workers^46^ studied the photodegradation efficiency of phenol in water (at different pH) under visible light irradiation in the presence of H_2_O_2_, using Pd3 (Fig. 4) immobilized on a mesopolymer (Table 1). The photodegradation rate could achieve 61% and 69% at acidic and neutral pH values within 420 min. On the other hand, there was 98% phenol degradation at alkaline pH after 360 min. Also, H_2_O_2_ was essential for the photocatalytic process as it increased from 56% to 98% under the same conditions when H_2_O_2_ was added. After four cycles, the photodegradation of phenol remains unchanged using Pd1–FDU-15. The degradation products are the same as reported before.^45^

Xu and co-workers^47^ and Iliev and co-workers^48^ studied the photooxidation of phenol using TiO_2_ modified with Al3 and H_2_4 (Fig. 4) in aqueous media under visible light irradiation (*λ* > 400 nm). The first authors compared the photooxidation rates with that obtained with H_2_4 immobilized on Al_2_O_3_. This rate was obtained by measuring the amount of CO_2_ produced at the end of the catalytic process. When using H_2_4–TiO_2_ (1.50 mol CO_2_/mol substrate), the photooxidation rate is much higher than H_2_4–Al_2_O_3_ (0.95 mol CO_2_ per mol substrate). The second authors could efficiently degrade phenol (∼90%) within 590 min with an optimum amount of Al1 (Table 1) loaded on a TiO_2_ (1.0 wt%). Concerning the main ROS involved in the photodegradation process, Iliev^48^ reported O_2_˙^−^ and HOO˙, which could lead to the same products reported before by these authors.^45^ Xu and co-workers^47^ identified the same ROS as Iliev^48^ but did not report the degradation products. None of these works mentioned the photostability studies of photocatalysts.

Yang and co-workers^49^ studied the photodegradation of phenol in aqueous media under visible light irradiation (*λ* ≥ 420 nm) in the presence of H_2_O_2_ (Table 1) and using Fe4 (Fig. 4) immobilized on graphene nanosheets (Fe4/GR) as the photocatalyst. According to the authors, the π–π stacking interaction between Fe4 and graphene (GR) forms a donor–acceptor system. The loading of Fe4 promotes the exfoliation of the graphene sheets and enables the dispersion of Fe4 on graphene. As expected, the introduction of Fe4 into the GR greatly enhanced the photocatalytic activity of the composites since the π–π interactions between the planar aromatic GR and Fe4 enable the electron transfer from the donor (Pc) to the acceptor (support). Within 180 min of irradiation, the GR/Fe4-0.25 (25 wt%) could achieve a photocatalytic rate of 77%, compared with Fe4/Al_2_O_3_ and Fe4/CNT, which were unable to degrade phenol. For the other materials used, such as Al_2_O_3,_ the donor–acceptor system does not occur. The Fe4/GR could be reused up to 4 times without significant loss of activity (10%). In this study, the authors analysed the mechanism involved in forming the ROS (O_2_˙^−^, HOO˙, and ˙OH) but did not identify the main degradation products nor the stability and photostability of the used materials.

Xu and co-workers^50^ studied the photodegradation of phenol under visible light irradiation (*λ* > 450 nm) using AlCl4, Cu4, and Co4 (Fig. 4) immobilized into modified bentonite (with the surfactant cetyltrimethylammonium bromide). The best photooxidation rate was achieved using AlCl4 as a catalyst (complete degradation after 240 min of irradiation). The authors also performed studies involving other phenols, such as 4-chlorophenol (4-CP), 4-nitrophenol (4-NP), 2,4-dichlorophenol (2,4-DCP), and 2,4,6-trichlorophenol (2,4,6-TCP), which will be discussed later. Moreover, the photooxidation rate of phenol suffers a gradual loss of activity after 4 cycles of the experiment using AlCl4. This loss of activity might be due to the degradation of the surfactant by the generated singlet oxygen or by the reduction of the sorption process that seems essential for the degradation process.

#### 3.1.2. 4-Methylphenol (*p*-cresol)

4-Methylphenol or *p*-cresol has been shown to cause uremia (retention of solutes by healthy kidneys). This molecule is part of the protein-bound uremic toxin milieu. The toxicological effects of *p*-cresol are related to its metabolism end products.^111,112^ It is highly resistant to natural degradation and can persist in the environment. This persistence and its harmful characteristics require a specific treatment given that the current methods have serious drawbacks like extreme operating conditions and the generation of harmful intermediates. Given all of this, it is necessary to find new alternatives to degrade this pollutant.^113^

FDU is a hexagonal mesoporous material, and this type of mesopolymer material has high physicochemical stability (despite the pH variation of the solution) and can adsorb phenolic pollutants from wastewater through π–π interactions and hydrogen bonding.^114,115^ So, Xing and co-workers^51^ studied the photocatalytic degradation of 4-methylphenol under visible light irradiation by using a palladium phthalocyanine Pd3 (Fig. 4) graphed through π–π interactions onto the FDU-14 mesopolymer (FDU-14-Pd3). After 180 min in basic conditions and with H_2_O_2_, it was possible to achieve 97% degradation of 4-methylphenol. Only 76% and 79% degradation rates were achieved in acidic and neutral conditions, respectively. This can be explained by the fact that 4-methylphenol must be deprotonated to 4-methylphenolate, which is more susceptible to oxidizing agents. Adding H_2_O_2_ to the photocatalytic system improved the degradation rate from *ca*. 40% to 97% after 180 min of light irradiation. In the dark, there is no degradation of 4-methylphenol. The degradation rate of 4-methylphenol remained unchanged after the experiment four times using the FDU-14-Pd3 photocatalyst. Similar photooxidation rates of 4-methylphenol were obtained using Pd3-FDU-15.

#### 3.1.3. 4-Nitrophenol

4-Nitrophenol (4-NP) is widely used in synthesising pesticides, drugs, and dyes in industries that release it into the environment. Acute exposure to 4-NP leads to liver and kidney damage, cancer, and systemic poisoning. In case of direct discharge in wastewater, it can endanger public health.^116,117^ So, developing new methods to eliminate this threat.

**Fig. 6:**
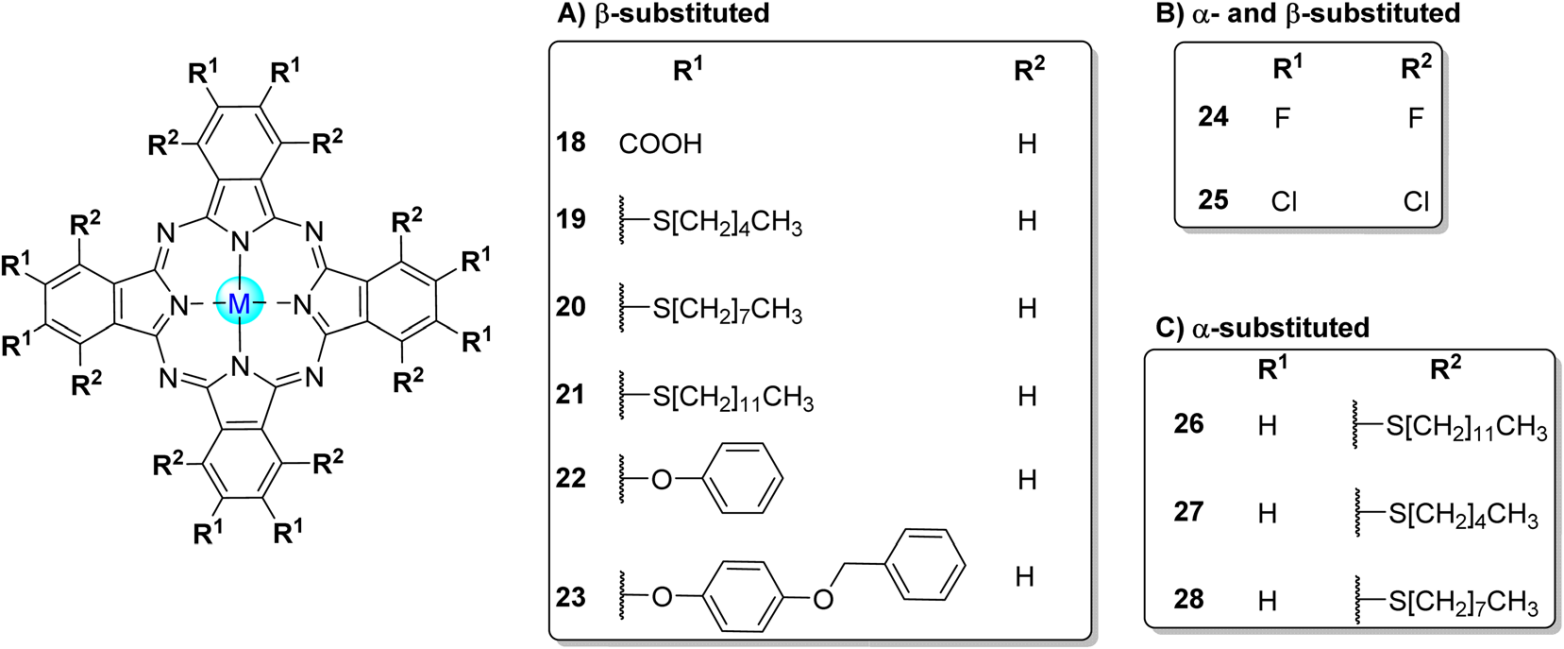
Symmetrical octa-substituted phthalocyanines in the (A) β-position 18,^52,54,103–106^19–23,^97,107^ (B) β- and α-positions 24,^53,108^25^53,86,109^ and (C) α-position 26,^97,110^27^97^ and 28.^97^

Nyokong and co-workers^52,84,97,118^ reported the photodegradation of 4-NP in aqueous or organic media in the presence of a large diversity of phthalocyanines. In a first study,^52^ the water-soluble octacarboxy Zn18 (Fig. 6) and a mixture of mono-, di-, tri-, and tetra-sulfonated zinc(ii) phthalocyanines (ZnPcS_mix_) were used as photocatalysts, and the experiments were carried out in aqueous solutions at pH = 8.2. The authors concluded that the quantum yields of 4-NP degradation were closely correlated with the singlet oxygen quantum yields (*Φ*_Δ_) and the phthalocyanines' aggregation (Table 2). Zn18 showed the highest *Φ*_Δ_ (*Φ*_4NP_ = 6.5 × 10^−3^ and 29% aggregation) followed by ZnPcS_mix_ (*Φ*_4NP_ = 7.9 × 10^−4^ and 49% aggregation) and the Zn3 (*Φ*_4NP_ = 1.5 × 10^−4^ and 78% aggregation).^52^ However, in this study, the most effective catalyst was ZnPcSmix since Zn18 degrades readily during catalysis. The products of the photodegradation of 4-NP were identified as hydroquinone and 4-nitrobenzene-1,2-diol.

**Table 2:** Photophysical parameters regarding the degradation of 4-NP with several derivatives 1–12, 19–21, 28, 45–48^52,53,84,97,107,118,119^

MPc	Support	Light	*Φ*_Δ_	*t*_1/2_ (min)	Efficiency (*Φ*, % or rate)	Recycle	Ref.
Zn3	—	Visible light (*λ* = 600 nm)	<0.01	—	1.5 × 10^−4^^a^	—	52
Zn18			0.52		6.5 × 10^−3^^a^		
ZnPc_mix_			0.48		7.9 × 10^−4^^a^		
Al4Cl	Surfactant modified bentonite	Visible light (*λ* > 450 nm)	—		73%^b^		50
Lu7OAc	PS	Visible light (*λ* > 400 nm)		61.9	—		97
Lu8OAc				72.8			
Zn4				93.7			
Lu29OAc			0.22	63.9			
Lu7OAc			0.28	50.2			
Lu12OAc			0.17	35.9			
Lu8OAc			0.15	37.9			84
	PSU		—	0			
Zn10			0.24	57.3			
Pd19	SWCNTs	Visible light (*λ* > 400 nm)	0.22	—	1.52 × 10^−3^	3 cycles (7% loss)	118
Pd20			0.22		1.32 × 10^−3^		
Pd21			0.27		1.74 × 10^−3^		
Pd22			0.19		0.92 × 10^−3^		
Pd23			0.2		1.27 × 10^−3^		
Pd26			0.20		1.86 × 10^−3^		
Pd27			0.23		1.67 × 10^−3^		
Pd28			0.21		1.33 × 10^−3^		
Pd19	—	Visible light (*λ* = 600 nm)	0.38		14 × 10^−4^^c^	—	107
Pd20			0.36		15 × 10^−4^^c^		
Pd21			0.39		18 × 10^−4^^c^		
Pd22			0.30		9.0 × 10^−4^^c^		
Pd23			0.32		16 × 10^−4^^c^		
Pt22			0.29		0.3 × 10^−4^^c^		
Pt23			0.26		0.7 × 10^−4^^c^		
Mg4			0.40		25%^d^		53
Al4Cl			0.29		89%^d^		
Zn4			0.67		53%^d^		
Zn5			0.11		45%^d^		
Zn6			0.11		54%^d^		
Zn24			0.13		75%^d^		
Zn25			—		23%^d^		
Cu13	TiO_2_	UV			100%^e^		120
H_2_13					50%^e^		
No					86%^e^		

a Light intensity (photons per s per cm^2^): 4.1 × 10^16^.

b After 240 min of irradiation.

c Light intensity (photons per s per cm^−2^): 3.5 × 10^20^.

d 5.0 × 10^16^.

e After 60 min of irradiation.

In another study,^107^ a set of water-insoluble palladium(ii) and platinum(ii) phthalocyanines (Pd19–Pd23 and Pt22, Pt23, Fig. 6 and Table 2) were used as photocatalysts. In terms of quantum yield, the Pd(ii) complexes exhibited better results than the Pt(II) ones being Pd21 (*Φ*_4NP_ = 18 × 10^−4^) the best photocatalyst followed by Pd23 (*Φ*_4NP_ = 16 × 10^−4^), Pd20 (*Φ*_4NP_ = 15 × 10^−4^), Pd19 (*Φ*_4NP_ = 14 × 10^−4^), Pd22 (*Φ*_4NP_ = 9 × 10^−4^), Pt23 (*Φ*_4NP_ = 0.7 × 10^−4^), and Pt22 (*Φ*_4NP_ = 0.3 × 10^−4^). The experiments were carried out in dichloromethane in the presence of triethylamine. The degradation products were identified as hydroquinone and 1,4-benzoquinone. Concerning the (photo)stability of the catalysts, the authors only mentioned that Pd7 was stable during the photocatalytic studies.

The degradation of 4-NP in aqueous media under visible light irradiation (*λ* = 600 nm) using the water-insoluble MPc complexes Mg4, Zn4, Al4Cl (Fig. 4), Zn5, Zn6 (Fig. 4), Zn24, and Zn25 (Fig. 6) as photocatalysts was also accessed (Table 2).^53^ The best photocatalytic rate was obtained using Al4Cl (with 89 ± 8% degradation of 4-NP after 100 min), followed by Zn24 (75%), Zn6 (54%), Zn4 (53%), Zn5 (45%), Mg4 (25%), and Zn25 (23%). The photocatalytic activity of these MPcs involved Type I (radicals) and Type II (singlet oxygen) mechanisms. The major products of the photodegradation of 4-NP were identified as 4-nitrobenzene-1,2-diol, hydroquinone, 1,4-benzoquinone, and fumaric acid. The authors did not report any studies regarding the stability of the photocatalysts during the assay.

**Fig. 7:**
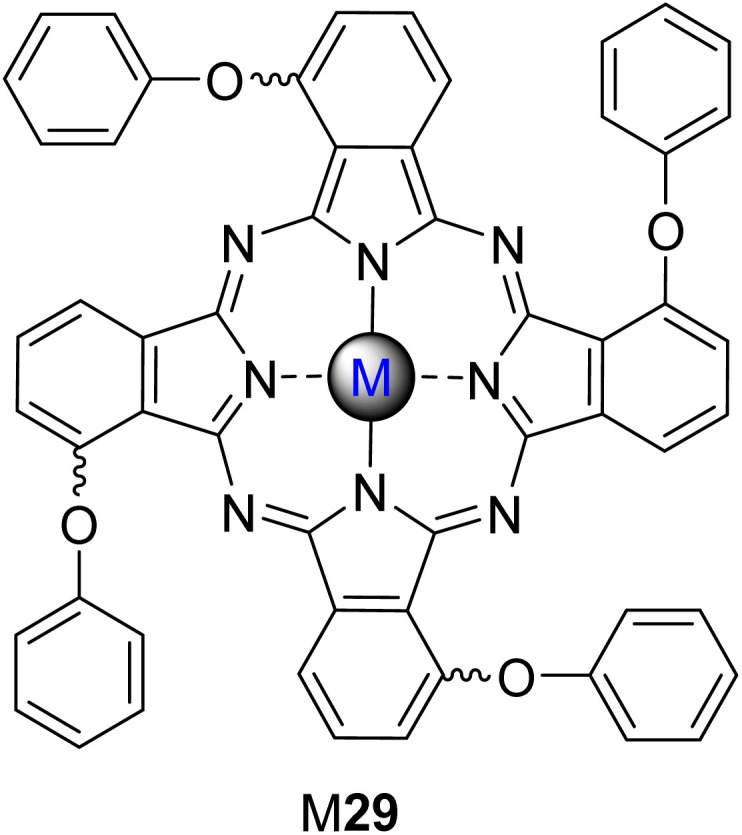
Lu(iii)29OAc α-substituted phthalocyanine complex used by Nyokong and co-workers.^84,97,121^

The same authors^97^ studied the photooxidation of 4-NP under visible light irradiation (*λ* > 400 nm) in aqueous media (pH = 8.2) when using tetra-substituted phthalocyanines bearing phenoxy and 2-pyridyloxy groups (7 and 8, Fig. 4) that were immobilized in polystyrene (PS) polymer fibre (Table 2). There was no photocatalytic degradation of 4-NP when using the non-immobilized PS fibres. However, the photodegradation of 4-NP was observed using supported Lu7OAc and Lu8OAc, being slightly higher when using Lu7OAc/PS fibre (*t*_1/2_ = 61.9 min^−1^) than using Lu8OAc/PS fibre (*t*_1/2_ = 72.8 min^−1^) or Zn4/PS fibre (*t*_1/2_ = 93.7 min^−1^). Table 2 compares the photoactivity of Lu7OAc (Fig. 4) with that of the isomeric α-substituted Lu29OAc (Fig. 7), indicating that substituents at β-positions improve photocatalysis. In this study, the major degradation products of 4-NP were hydroquinone and 4-nitrobenzene-1,2-diol, with a slight formation of 1,4-benzoquinone. Regarding the (photo)stability of the catalysts, it is only mentioned that a slight photodecomposition of Lu8OAc was observed after 12 h of irradiation.

Nyokong and co-workers^84^ studied the photodegradation of 4-NP, 4-CP, and methyl orange (MO) under aqueous media (pH = 12) using a series of peripherally substituted zinc(ii) and Lu(iii)OAc phthalocyanine complexes 6–12 (Fig. 4) and 29 (Fig. 7) immobilized on PS, PSU, PAA, and PUR (Tables 2, 3 and 7). Different phthalocyanine complexes were immobilized in several polymers: (i) Zn7, Zn8, Zn12, Lu29OAc–7, 8, and Lu12OAc in PS polymer fibres; (ii) Zn9, Zn10, and Lu8OAc in PSU polymer fibres; and (iii) Zn6, Lu6OAc, and Lu10OAc in the PAA and PUR fibres. Complex Lu11OAc was covalently linked to PUR by amide bonds. Similarly, Zn6 and Lu6OAc were covalently linked to PAA through amide bonds. The three phthalocyanines were mixed with PAA and PUR to assess the difference in the photocatalytic activity between the hybrids with or without a chemical bond.

**Table 3:** Photophysical parameters for the degradation of 4-CP under visible light irradiation (*λ* > 400 nm)

MPc	Support	*Φ*_Δ_	Irradiation time (min)	*t*_1/2_ (min)	Efficiency (%)	Recycle	Ref.
Al4Cl	Bentonite	—	300	—	75	—	50
Lu29OAc	PS		—	68.6	—		121
Zn4				1824.1			
Lu29OAc		0.22	—	63.6		—	84
Lu7Ac		0.28		50.2			
Lu8OAc		0.17		64.8			
Lu12OAc		0.15		70.7			
Zn6		a		—			
Lu6OAc							
Zn9	PSU	0.21		63.6			
Zn10		0.24		57.3			
Lu8OAc		0.26		—			
Lu11OAc	PUR	0.11					
In30Cl	PS	—	866.0				98
In9Cl			217				
In31Cl			182.0				
Zn14	AuNPs- PS		17.8				101
	PS		52.1				
Al3	Amberlite	30	—		70		54
Pd26	SWCNTs	—	1440		42		110
	—		—		91		
Zn2	g-C_3_N_4_		90		98	10 cycles (<5% loss)	55
	g-C_3_N_4_/PAN		270			5 cycles (<5% loss)	56
Al3	TiO_2_		360		100	—	47
Co3			90		90		57
					100^b^		
					95		58
Co15			30		100	5 cycles (9% loss)	35
Zn15						5 cycles (12% loss)	
Co15			30		99	—	99
Zn15					98		
Co32			120		88		36
Zn32					90		
Co33					85	5 cycles (37% loss)	
Zn33					86	5 cycles (34% loss)	
Without Pc					3	—	

a Not determined due to the solubility of the polymer in water.

b With H_2_O_2_.

According to the authors, the PAA polymer fibres were unsuitable for application in aqueous media due to their extensive solubility that induces a gelatinous solution. No photocatalytic degradation of any pollutants occurred even after 720 min of irradiation using the covalently linked Lu11OAc–PUR fibres. The same occurred for the fibres where Lu11OAc and PUR were mixed. These results contrast those reported for Lu29OAc and Lu7Oac, which are very photoactive materials.^97,122^ The absence of photocatalytic activity can be related to the morphology of the fibres because they are not porous enough for sufficient interaction between the reactive species and the photoactive phthalocyanines.

The PS and PSU polymer fibres are insoluble in water and suitable for photocatalytic degradation assays. There was a 40% degradation of 4-NP under visible light irradiation (*λ* > 400 nm) after 150 min of irradiation when using Lu7OAc/PS, Lu8OAc/PS, Lu12OAc/PS, and Lu29OAc/PS (Table 2). It was possible to obtain a better photocatalytic degradation rate when using the unquaternized Zn8/PS fibres compared to the quaternized Zn12/PS fibres. The 4-NP could be degraded more efficiently using the peripherally substituted Zn7/PS fibres compared to their non-peripheral analogue (Lu29/PS). The photooxidation rate of 4-NP could be maintained even after the photocatalyst was reused up to three times. Regarding the stability of these catalysts, the author only mentioned that the PSU functionalized with metal phthalocyanines was relatively stable under the same light intensity as in the photocatalytic studies. It is important to mention that after the first cycle of the reusability studies when dried, these fibres folded up, forming a hard lump, and could not be applied again. The 1,4-benzoquinone and hydroquinone were identified as the major products of the 4-NP degradation.

In another study,^118^ they studied the degradation of 4-NP in aqueous media under visible light irradiation (*λ* = 400–600 nm) using composites based on the adsorption of several palladium(ii) phthalocyanines 19–23 (Fig. 6) and 26–28 (Fig. 6) in single-walled carbon nanotubes (SWCNTs, Table 2). In basic media (pH = 8.5), the photocatalytic rate using all composites follows the order: 26/SWCNTs (*k*_obs_ = 1.86 × 10^−3^ min^−1^) > 21/SWCNTs (*k*_obs_ = 1.74 × 10^−3^ min^−1^) > 27/SWCNTs (*k*_obs_ = 1.67 × 10^−3^ min^−1^) > 19/SWCNTs (*k*_obs_ = 1.52 × 10^−3^ min^−1^) > 28/SWCNTs (*k*_obs_ = 1.33 × 10^−3^ min^−1^) > 20/SWCNTs (*k*_obs_ = 1.32 × 10^−3^ min^−1^) > 23/SWCNTs (*k*_obs_ = 1.27 × 10^−3^ min^−1^) > 22/SWCNTs (*k*_obs_ = 0.92 × 10^−3^ min^−1^). It was possible to obtain a better photodegradation of 4-NP for the composites bearing long alkyl chains. There was no significant loss of degradation rate of 4-NP (∼7%) after three cycles of the experiment. This study identified 1,4-benzoquinone, hydroquinone, and 4-nitrobenzene-1,2-diol as the major products of the degradation process. All photocatalysts proved to be stable for 400 min.

Palmisano and co-workers^120^ studied the degradation of 4-NP in aqueous media using H_2_13 and Cu13 (Fig. 4) immobilized in polycrystalline TiO_2_ samples and investigated their photocatalytic behaviour in the degradation of (Table 2). The photocatalysts remained stable for 420 min under irradiation. Complete degradation of 4-NP was observed after 60 min irradiation with UV light and using 1% or 1.5% Cu13/TiO_2_. On the other hand, there was only 50% degradation after 60 min irradiation (but complete degradation after 5 h) when using H_2_13/TiO_2_. Compared with bare TiO_2_ (86% degradation), only Cu13/TiO_2_ showed enhanced photocatalytic activity.

Xu and co-workers^50^ accessed the degradation of 4-NP in an aqueous medium at pH 11.2 under visible light irradiation (*λ* > 450 nm) using Al4Cl (Fig. 4) inserted into modified bentonite (with the surfactant cetyltrimethylammonium bromide) (Table 2). It was possible to achieve 73% degradation of 4-NP after 290 min of irradiation. For this pollutant, no degradation products were identified.

#### 3.1.4. 4-Chlorophenol

4-CP belongs to a group of toxic environmental pollutants that must be eliminated. Some conventional treatments like chlorination and adsorption currently perform their removal. However, they have some drawbacks, like requiring longer treatment to break down organic pollutants or generating carcinogenic by-products. Thus, photocatalysis is a promising alternative.^123^ Xu and co-workers^50^ could achieve 71% degradation of 4-CP under visible light irradiation (*λ* > 450 nm) in aqueous media using Al4Cl (Fig. 4) inserted into modified bentonite (Table 3).

**Fig. 8:**
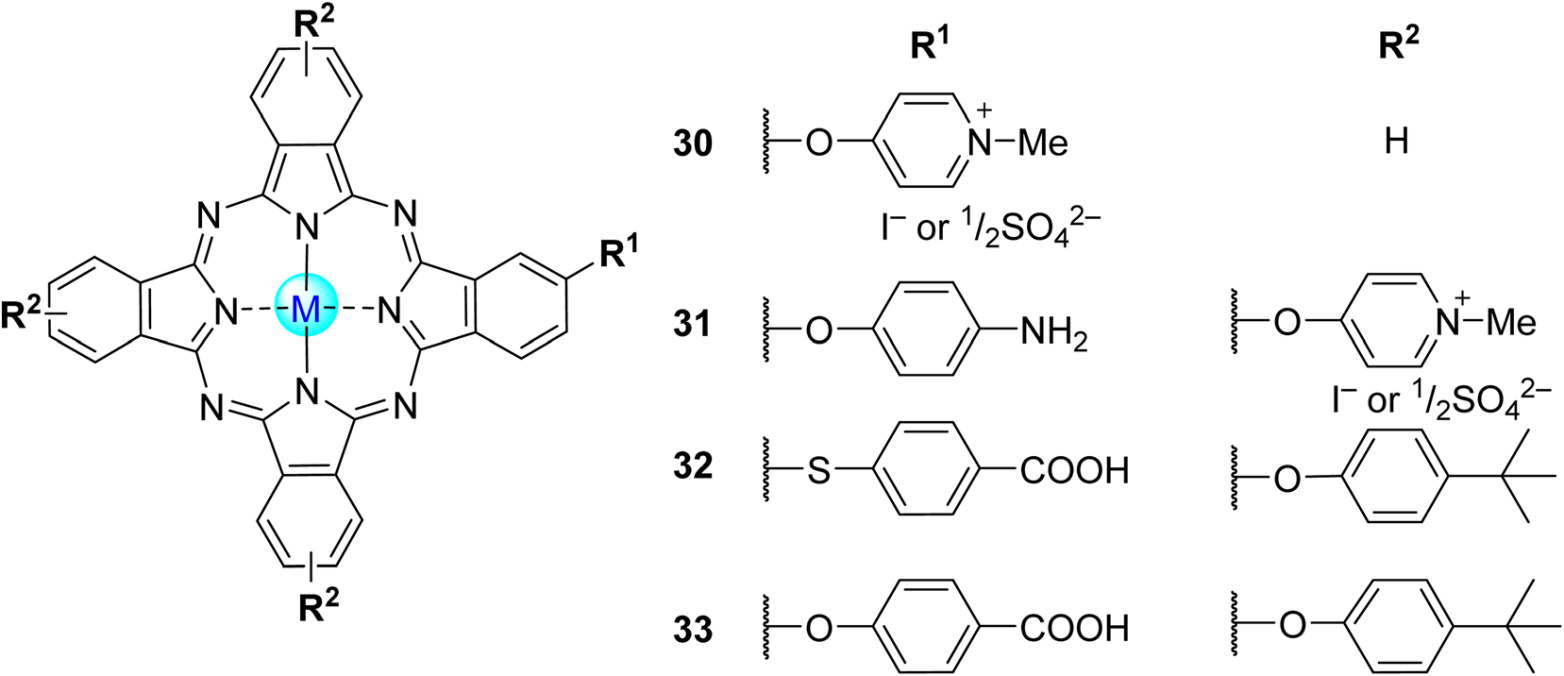
Non-symmetrical tetra-β-substituted phthalocyanines 30, 31 used by Nyokong and co-workers,^98^ and 32, 33 used by Sevim and co-workers.^36^

Nyokong and co-workers^84,98,121^ studied the degradation of 4-CP under visible light irradiation (*λ* > 400 nm) in aqueous media (pH = 12) using different phthalocyanine complexes immobilized in several polymers: (i) Zn7, 8, Lu7OAc, Lu8OAc (Fig. 4) and Lu21OAc (Fig. 6), In9 and Lu12OAc (Fig. 4), 30 and 31 (Fig. 8) in PS polymer fibres; (ii) Zn9 (Fig. 4), Zn10 (Fig. 4), and Lu8OAc (Fig. 4) in PSU polymer fibres; and (iii) Zn6, Lu6OAc (Fig. 4), and Lu10OAc (Fig. 4) in the PAA and PUR fibres (Table 3). Regarding PS fibres, these authors^101^ also prepared Zn(ii) phthalocyanine 14 (Fig. 4 and Table 3)–gold nanoparticles (AuNPs) conjugates immobilized on PS fibres. Besides polymer fibres, Nyokong and co-workers^54^ immobilized Al1 (Fig. 3) on Amberlite^®^ and supported the Pd(ii) phthalocyanine Pd26 (Fig. 6) on SWCNTs functionalized with carboxylic acid moieties.^110^

Overall, in the studies performed by Nyokong and co-workers,^54,84,98,101,110,121^ despite the catalyst used, the degradation products were hydroquinone and 1,4-benzoquinone. In the first study,^121^ upon visible light irradiation (*λ* > 400 nm) and during 720 min, the prepared composite could degrade 4-CP, showing a half-life of 30 min for the smallest concentration of 4-CP (0.156 mM). Even at the highest concentration of 4-CP (0.506 mM), the half-life is only 68.6 min (Table 4). Moreover, the Zn4 (Fig. 4)/PS fibre was also used for comparison and showed half-times much higher (1155.3 min for 0.156 mM and 1824.1 min for 0.506 mM, respectively) than the prepared Lu29OAc/PS fibre. However, after 720 min of continuous light irradiation, a photodegradation of the Lu29OAc (Fig. 7) could explain the incomplete degradation of 4-CP. The Zn4/PS fibres were degraded entirely after 720 min of irradiation. In the second study,^84^ it was possible to observe a degradation of 4-CP after 150 min under visible light irradiation (*λ* > 400 nm) using photoactive materials of Lu7OAc/PS, Lu8OAc/PS, Lu12OAc/PS (Fig. 4), and Lu29OAc/PS (Fig. 7) (Table 3). The hybrids can degrade 4-CP faster (complete degradation after 150 min) when compared with 4-NP (∼40% of degradation after 150 min). There was a faster degradation of 4-NP when using the peripherally substituted Zn7/PS fibres rather than the non-peripheral analogue (Lu29/PS).

**Table 4:** Efficiency of Co11/Zn11–TiO_2_ composites for the photocatalytic degradation of 4-CP^99^

Catalyst	Photocatalyst activity (% of degradation)				
	1st cycle	2nd cycle	3rd cycle	4th cycle	5th cycle
Co11–TiO_2_	99	97	95	92	91
Zn11–TiO_2_	98	95	93	90	88

In another study, the authors^98^ observed that a higher degradation rate constant of 4-NP was obtained using In31Cl/PS (*t*_1/2_ = 182.0 min) fibre followed by In9Cl/PS (*t*_1/2_ = 217.0 min) and In30Cl/PS (*t*_1/2_ = 866.0 min) – Table 3. The high activity of In31Cl/PS could be due to the high singlet oxygen generation values (*Φ*_Δ_ = 0.50) and the high surface area of their fibres. The number of charges did not seem to influence the photocatalytic activity of the conjugates since In9Cl/PS has more charges than In31Cl/PS and lower photocatalytic activity.

Regarding gold nanoparticles,^101^ high degradation rate constants and low lifetimes were observed for the Zn14–AuNPs/PS fibres (Fig. 4 and Table 3). Also, it was possible to achieve higher degradation rates when coupling AuNPs. In a study with Amberlite, Nyokong and co-workers^54^ observed that the composite degraded ∼70% of 4-CP after 30 min of irradiation. Regarding this pollutant, no degradation products were identified. The authors extended the photocatalytic study to 2,4-CP, 2,4,6-TCP, and pentachlorophenol (PCP). Moreover, the study was extended to other metallated phthalocyanines for this last organic pollutant. Regarding carbon materials, Nyokong and co-workers^110^ compared the degradation efficiencies of 4-CP when using Pd19/SWCNTs and Pd26 in aqueous media under visible light irradiation (Fig. 6 and Table 3). Pure Pd26 exhibited higher photocatalytic activity (91% for 4-CP) than the hybrid material (42% for 4-CP). The Pd26/SWCNTs catalyst cannot be reused without significant activity loss. Despite the low photocatalytic rates of the Pd26/SWCNTs hybrid, Pd26 had excellent photocatalytic activity and may be applied in homogenous photocatalysis. Chen, Lu and co-workers^55^ studied the photooxidation of 4-CP in aqueous media under visible light (*λ* > 400 nm) using visible light-responsive photocatalysts based on g-C_3_N_4_ and polyacrylonitrile (PAN)-supported g-C_3_N_4_ coupled with zinc(ii) phthalocyanine 2 (g-C_3_N_4_/Zn2and g-C_3_N_4_/Zn2/PAN, Fig. 4. Zn2 was covalently bonded to g-C_3_N_4_ through the carboxy group of the MPc and an amine of g-C_3_N_4_ (to form an amide). After 90 min of visible light irradiation, ∼98% of 4-CP was degraded by this photocatalyst, which is higher when compared with bare g-C_3_N_4_ and Zn2 (Table 3). These results are due to the synergistic effect between g-C_3_N_4_ and Zn2. More importantly, the photocatalytic degradation of 4-CP was as high as 99% after ten cycles, with no decrease, indicating that the catalyst has broad application for removing organics in polluted waters. The authors did not report the degradation products nor the stability of the photosensitizers under irradiation.^55^

In a new report,^56^ the authors studied the photooxidation of 4-CP using g-C_3_N_4_/Zn2 (Fig. 4) as the catalytic entity, and PAN nanofibers were employed as support to overcome the shortcomings of easy aggregation and to enable an easy recycling process. In the visible light studies, the removal rate of 4-CP using g-C_3_N_4_/Zn2/PAN as photocatalyst was higher than with g-C_3_N_4_/PAN, reaching a maximum of ∼98% after 270 min of irradiation (Table 3). The g-C_3_N_4_/Zn2/PAN was reused up to five times without significant loss of activity. In both cases, the authors did not refer to the stability of the catalysts (effect of photobleaching) under irradiation nor the degradation products of 4-CP.

Several studies were performed regarding the degradation of 4-CP with MPcs supported on TiO_2_. For example, Xu and co-workers^47^ evaluated the photocatalytic ability of Al2 (Fig. 4 adsorbed on TiO_2_ and could achieve a complete degradation after 420 min of visible light irradiation with an optimum amount of Al2 loaded on TiO_2_ of 1.0 wt% (Table 3). Also, the authors could perform four photocatalytic cycles with a decrease of 40% in the photodegradation rate, which can be due to the photobleaching of Al2 during the photocatalytic assay. 1,4-Benzoquinone, hydroquinone, formic and acetic acid were identified as the degradation products of 4-CP.

Pirbazari and co-workers^57,58^ studied the photocatalytic activity, in aqueous media, of 4-CP by using Co(ii)3 immobilized on TiO_2_ nanoparticles. In both studies,^57,58^ after 90 min under visible light irradiation (*λ* > 400 nm), it was possible to degrade 50% of 4-CP with Co3–TiO_2_, which could be increased to ∼100% in the presence of H_2_O_2_ (Table 3). These results are auspicious; however, recyclability studies are crucial to determine the potential use of this photocatalyst in wastewater treatment. In this sense, in the second study,^58^ a decrease in the efficiency of the degradation process of 95% to 60% (in the presence of H_2_O_2_) after four photocatalytic studies was observed. In this study, the photocatalytic experiments were extended to 2,4-DCP. In both studies,^57,58^ the authors reported carboxylic acids and CO_2_ as the degradation products of 4-CP.

Two studies were developed by Gül and co-workers^35,99^ where they studied the degradation of 4-CP after the incorporation of Co(ii) and Zn(ii) Pcs Co/Zn11 and Co/Zn15 (Fig. 4) into TiO_2_ semiconductors. The results showed that under visible light (*λ* > 400 nm), it was possible to achieve complete degradation of 4-CP in aqueous media after 30 min with Zn11, Co11, Zn15, and Co15 (Table 3). Moreover, they observed that the MPcs were anchored into the surface of TiO_2_ through CO–O–TiO_2_ bonds. In the first study,^99^ recyclability studies showed a loss of 16% in the degradation of the pollutant using Zn11 and Co11 after 5 cycles (Table 4). In the second study,^35^ after 5 cycles of the experiment, the majority of the photodegradation rate of 4-CP could be maintained (∼9% decrease) using Co15. On the other hand, there was a decrease of ∼16% in the degradation of 4-CP with Zn15. Herein, the authors extended the photocatalytic assays to chlorobenzene (PhCl) and 1,2,4-trichlorobenzene (TCB). None of these studies revealed the stability of the compounds under irradiation nor the degradation products of 4-CP.

Sevim^36^ developed a study regarding the photooxidation of 4-CP under visible light irradiation (*λ* > 400 nm) using non-symmetrical tetra-substituted phthalocyanine bearing one carboxy group (either 4-sulfanylbenzoic acid or 4-hydroxybenzoic acid) and three 4-*tert*-butylphenoxy substituents and their zinc(ii) and cobalt(ii) complexes (32 and 33, Fig. 8) immobilized on TiO_2_ as catalysts. The MPcs were anchored on the surface of TiO_2_ through CO–O–TiO_2_ bonds (Table 3). It was possible to achieve ≥85% of 4-CP degradation within 120 min of irradiation using all photocatalysts compared with bare TiO_2_ (3%). The best photocatalytic rate was obtained with Co33 (90%), followed by Co32 (88%), Zn33 (86%), and Zn32 (85%). The reusability studies showed that, after five cycles, there was a decrease in the photooxidation rate from 90% to 66% when using Co33 and from 85% to 63% when using Zn32. The degradation products of 4-CP were not revealed.

#### 3.1.5. Dichlorophenols

The contamination of wastewater with dichlorophenols is considered a severe threat. The toxicity of chlorophenol depends on the degree of chlorination and the substitution away from the *ortho*-position. There is reported increasing toxicity related to higher chlorinated phenolic compounds. The treatment of chlorophenols in wastewater is crucial to decrease their toxicity in water.^124^ Regarding the degradation of 2,4-DCP, Xu and co-workers^50^ could achieve a complete degradation after 60 min of visible light irradiation (*λ* > 450 nm) by using Al4Cl–bentonite (modified with the surfactant cetyltrimethylammonium bromide) (Fig. 4) in aqueous media (pH = 12, Table 5). The photocatalyst's (photo) stability was neither reported nor recyclable studies. Moreover, the final products of the degradation process were not identified.

**Table 5:** Photophysical parameters for the degradation of 2,4-DCP

MPc	Support	Light	Irradiation time (min)	Efficiency (%)	Recycle	Ref.
Al4Cl	Surfactant modified bentonite	Visible light (*λ* > 450 nm)	60	100	—	50
Al3	Amberlite	Visible light (*λ* > 400 nm)	30	40		54
Co3	MCM-41	UV-A (*λ* = 280–400 nm)	180	45		59
				93^a^	4 cycles (35% loss)	
Al3	TiO_2_	Visible (*λ* > 400 nm)	360	100	—	47
Co3			180	95		
				50	4 cycles (40% loss)	58
				100^a^		
Co4	N-GR		135	80	3 cycles (25% loss)	60
Zn4	g-C_3_N_4_		60	85	–	61

a With H_2_O_2_.

Nyokong and co-workers,^54^ on the other hand, could achieve ∼40% degradation of 2,4-DCP in aqueous media after 30 min of visible light irradiation (*λ* > 400 nm) by using Al3 immobilized on Amberlite^®^ (Fig. 4 and Table 5). 2-Chloro-1,4-benzoquinone and formic acid were identified as the degradation products of 2,4-DCP. No stability or recyclability studies were reported for the catalyst.

The photodecomposition of 2,4-DCP in aqueous media was also assessed using sulfonated cobalt(ii) phthalocyanine Co3 (Fig. 4) immobilized onto MCM-41.^59^ The immobilization of Co3 onto MCM-41 was performed through the ionic interactions between the cationic groups of the 3-(aminopropyl)triethoxysilane and the sulfonato groups of the phthalocyanine. The authors stated that both light and catalyst are essential for the degradation of 2,4-DCP since it could only be observed in the presence of the catalyst and light (visible or UV-A). When irradiated with UV-A light for 180 min, Co3 (0.6 g L^−1^) exhibited a higher decrease of 2,4-DCP degradation (93%) than when irradiated with visible light (55%) in the presence of H_2_O_2_. As indicated in Table 5, the presence of H_2_O_2_ is essential when using the UV-A light – the sample containing H_2_O_2_ + Co3 could achieve 93% degradation after 180 min of irradiation, while only 45% degradation was observed in the sample containing only Co3. After 4 cycles under UV-A irradiation, the photocatalytic degradation of 2,4-DCP could maintain up to 70%. The authors identified methyl pyruvate, dimethyl oxalate, dimethyl malonate, methyl levulinate, and methyl benzoate as the primary intermediates of 2,4-DCP degradation. However, the final degradation products were simple acids like oxalic acid and acetic acid.

Xu and co-workers^47^ and Pirbazari and co-workers^58^ accessed the degradation of 2,4-DCPusing Al3 and Co3, respectively, adsorbed on TiO_2_ as photocatalysts. The first authors,^47^ could achieve a complete degradation in aqueous media after 420 min of visible light irradiation (*λ* > 400 nm) with 1.0 wt% of Al3 loaded on TiO_2_ (Table 5). There were no studies regarding the stability of the catalyst and the degradation products of 2,4-DCP. In the second study, they achieved 50% degradation of 2,4-DCP after 180 min of visible light irradiation (*λ* > 400 nm), which could be increased to 100% in the presence of H_2_O_2_. Moreover, after four cycles, there was a loss of 40% in the photocatalytic oxidation of 2,4-DCP using Co3–TiO_2_. Regarding the degradation products of 2,4-DCP, the authors identified carboxylic acids and CO_2_.

Recently, Frajood and co-workers^60^ could degrade 80% of 2,4-DCP within 135 min of visible light irradiation (*λ* > 400 nm) by using Co4/nitrogen-doped graphene (N-GR). According to the authors, after 3 cycles, there was a loss of 25% of activity. The authors identified carboxylic acids and CO_2_ as the degradation products of 2,4-DCP. Zada, Dong, Fu, and co-workers^61^ prepare a Zn4/g-C_3_N_4_ nanocomposite for the photodegradation of 2,4-DCP. The authors could achieve a degradation of 85% after 60 min of visible light irradiation (*λ* > 420 nm). The enhanced catalytic activity of this nanocomposite is due to its high visible light absorption and effective generation of super oxide anions and holes. However, no studies were performed regarding the recyclability of the catalysts or degradation products of 2,4-DCP.

#### 3.1.6. Trichlorophenols

Phenolic compounds such as trichlorophenols are widely used in the pharmaceutical, oil, paint, explosive, paper, and agrochemical industries. They are defined as priority pollutants. Their inappropriate disposal can damage the environment due to their potential toxicity to microorganisms, plants, and animals. These organic compounds are persistent and difficult to biodegrade.^125^

**Fig. 9:**
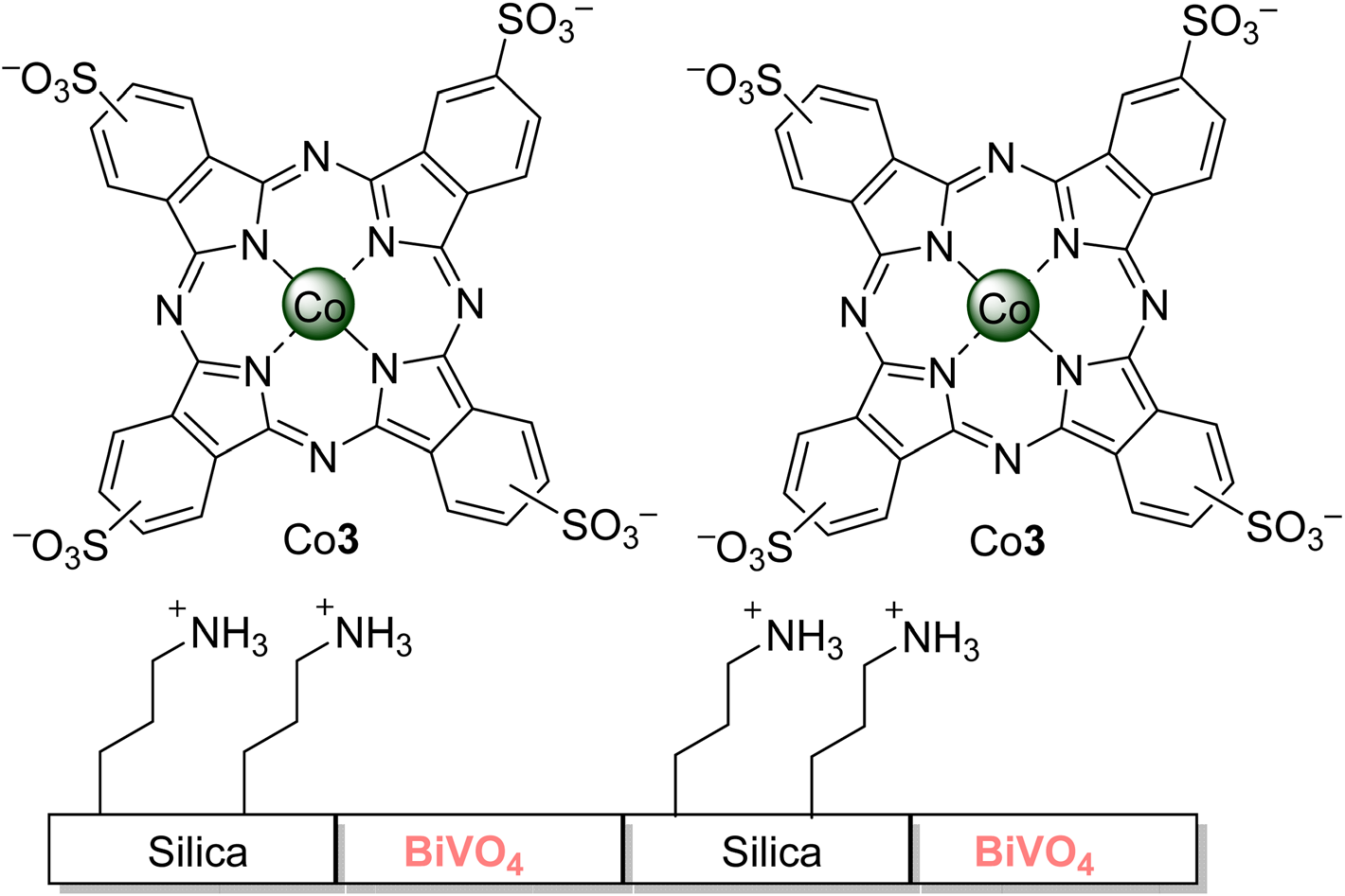
Silica–BiVO_4_ composites grafted with cobalt phthalocyanine Co3 used by Zanjanchi and co-workers^126^ for the photodegradation of 2,4,6-TCP.

Xu and co-workers^47,50^ reported two works regarding the degradation^58^ of 2,4,6-TCP. In the first study,^50^ a complete degradation was achieved in less than 60 min using visible light irradiation (*λ* > 450 nm) and Al4Cl–bentonite (modified with cetyltrimethylammonium bromide) in aqueous media (pH = 12). However, a gradual loss of activity was observed after each catalytic cycle, and it might be due to the degradation of the surfactant by the generated singlet oxygen or by reducing the sorption process. In the second work,^47^ the authors could achieve a 90% degradation in aqueous media after 420 min of visible light irradiation (*λ* > 400 nm) with 1.0 wt% of Al3 loaded on TiO_2_. No photostability studies of the catalysts nor the 2,4,6-TCP degradation products were reported. Nyokong and co-workers,^54^ on the other hand, could achieve 30% degradation in aqueous media after 30 min of visible light irradiation (*λ* > 400 nm) by using Al3 immobilized on Amberlite^®^ (Fig. 4). The 2,5-dichloro-1,4-benzoquinone was identified as the main degradation product of 2,4,6-TCP. Zanjanchi and co-workers^126^ assessed the photodegradation of 2,4,6-TCP in aqueous media using visible light irradiation (*λ* > 400 nm) and BiVO_4_–Silica composites grafted with sulfonated cobalt phthalocyanine Co3 (Fig. 9). Different catalytic activities were observed depending on the silica and loaded phthalocyanine amount. The best results (∼100% degradation after 240 min of irradiation) were obtained using the sample containing 15% silica grafted with Co3; materials without the Co3 could only degrade 50% of 2,4,6-TCP. Regarding the stability of the composite, there was a leak of 15.2%, and the composite could be recovered and reused up to 4 times without significant activity loss (3%). The degradation products were not identified, but the pH decrease during the assays could indicate the formation of carboxylic acids.

#### 3.1.7. Pentachlorophenol

Chlorinated phenols, particularly PCP, are extremely toxic environmental pollutants. Their degradation in water is complex and may lead to chlorinated dibenzo-1,4-dioxins and other organic compounds that may be more toxic than the parent compound.^127^

Nyokong and co-workers^54,110^ developed various materials for the degradation of PCP. In their first work,^54^ Al3, Zn3 (Fig. 4 and Table 6), Al18, Zn18 (Fig. 6 and Table 6), and a mixture of mono-, di-, tri-, and tetra-substituted sulfonated metalled phthalocyanines MPcS_mix_ (M = Al(iii), Zn(ii), Ge(iv), Si(iv), or Sn(iv)) were immobilized on Amberlite^®^. These materials were used as photocatalysts in the degradation of PCP in aqueous media (pH = 10) under visible light irradiation (*λ* > 400 nm). The composites showed the following activity order after 5 min of irradiation: Zn18 > SiPcS_mix_ > SnPcS_mix_ > ZnPcS_mix_ > GePcS_mix_ > Zn3 > AlPcS_mix_ ≈ Al18 > Al3. The Zn18/Amberlite was selected to perform the recyclability studies, where it was observed that there was only a 10% loss of activity after three cycles. However, when performing homogenous photocatalysis, there was a 51% degradation of the catalyst (Zn2) after 3 min of irradiation, which means that this phthalocyanine could not be applied to other pollutants as PCP since it needed more irradiation time. The tetrachloro-1,4-benzoquinone was identified as the major degradation product of PCP.

**Table 6:** Comparison of the photocatalytic degradation of PCP using different Pcs

Pc	% degradation
Zn18	30
SiPcS_mix_	26
SnPcS_mix_	24
ZnPcS_mix_	21
GePcS_mix_	17
Zn3	13
AlPcS_mix_	11
Al18	11
Al3	6

Later,^110^ they degraded PCP in a homogenous catalytic process in dichloromethane (DCM) with Pd26 (Fig. 6) and in a heterogeneous photocatalytic process in water with Pd26/SWCNTs, both under visible light irradiation (*λ* > 400 nm). It was possible to achieve a degradation rate of 70% and 30% in the homogenous and heterogenous processes, respectively. Again, tetrachloro-1,4-benzoquinone was identified as the major degradation product of PCP. The catalyst cannot be reused without a significant loss of activity. Regarding the recyclability studies using 4-CP, the activity loss after each cycle was less drastic than PCP. This can be due to the permanent adsorption of intermediates or products on the surface of the catalyst, thereby reducing the adsorption activity of the composite. Despite the low photocatalytic rates of Pd26/SWCNTs hybrid, Pd26 had excellent photocatalytic activity by itself and, thus, may be applied in homogenous photocatalysis.

Regarding silica materials, Pereira, Azenha, and co-workers^85^ studied the photooxidation of PCP with three zinc(ii) phthalocyanines Zn5, Zn7, and Zn16 (Fig. 4) immobilized into Al-MCM-41 in aqueous media. The best photocatalysts were Zn5/Al-MCM-41 (*k*_obs_ = 7.3 × 10^−4^ min^−1^) and the cationic derivative Zn16 (*k*_obs_ = 1.1 × 10^−3^ min^−1^) after 300 min of UV-visible light irradiation (*λ* = 320–460 nm). The authors identified tetrachloro-1,4-benzoquinone as the major degradation product. The photocatalytic studies were extended to fenamiphos, where the recyclability studies were performed.

#### 3.1.8. Other phenolic compounds

Other phenolic compounds like catechol, hydroquinone, salicylic acid, naphthol yellow S, and BPA are considered toxic to humans. Their elimination of wastewater must be prioritized.^128–131^

Xu and co-workers^47^ reported the degradation of hydroquinone, catechol, salicylic acid, and 4-sulfosalicylic acid in aqueous media using 1.0 wt% of Al2 (Fig. 4) loaded on TiO_2_ and visible light irradiation (*λ* > 400 nm). Here, hydroquinone and catechol were degraded at 90% and 50%, respectively, after irradiation for 420 min. However, only 20% and 10% degradation of salicylic acid and 4-sulfosalicylic acid were observed under similar conditions. Raducan and co-workers^86^ reported the photodegradation of naphthol yellow S using Cu3 (Fig. 4), Cu4 (Fig. 4), and Cu25 (Fig. 6) immobilized on TiO_2_. Notably, with Cu4, a maximum degradation of 18% was achieved after 30 min under visible light irradiation (*λ* > 400 nm). Jiang and co-workers^132^ studied the photocatalytic degradation of BPA in aqueous media using polynuclear phthalocyanines M1 (Fig. 3) under visible light irradiation (*λ* > 400 nm) for 40 min. Among the four complexes (Fe1, Cu1, Zn1, and Al1), the highest photocatalytic activity was obtained for Zn1. For this reason, only this photocatalyst was used to study the optimized conditions to degrade BPA. It is essential to highlight that Zn1 evidenced their decomposition during the photocatalytic process, and it is still possible to achieve a total degradation of the pollutant after 20 min using a tungsten lamp. With solar irradiation, the same result was achieved after 40 min. Regarding the degradation products, the authors could identify oxalic acid, hydroquinone, and 4-isopropenyl phenol.

Wu, Xing, and co-workers^46^ also studied the photodegradation of BPA but using Pd3 (Fig. 4) immobilized on FDU-15 mesopolymer. Herein, the photodegradation efficiency of BPA was also observed in aqueous media at different pH values, in the presence of H_2_O_2_, and under visible light irradiation. The pollutant was degraded within 60 min and 180 min when using 0.04 and 1.0 mmol L^−1^, respectively. The degradation products were identified as dimethyl malonate, dimethyl oxalate, dimethyl d-malate, and CO_2_. The stability and photostability of the catalyst were not evaluated. In another study, Nyokong and co-workers^98^ studied the photooxidation of BPA under visible light irradiation using indium(iii) using the phthalocyanines In9 (Fig. 4), 30, 31Cl/PS fibres (Fig. 8). The In31Cl/PS (*t*_1/2_ = 178.0 min) fibre was the best photocatalyst followed by In9Cl/PS (*t*_1/2_ = 267.0 min) and In30Cl/PS (*t*_1/2_ = 385.0 min).

The photodegradation of BPA under UV light irradiation (*λ* = 365 nm) in the presence of a ZnWO_4_/Mn17Cl material with 1 wt% Mn17Cl (Fig. 4) was investigated by Anucha and co-workers.^102^ It is highlighted that 60% of BPA was degraded after 4 h of light exposure, but the degradation could be increased to 80% by adding H_2_O_2_. However, a complete degradation of BPA could be achieved after 30 min only under visible light irradiation (*λ* > 450 nm) and adding H_2_O_2_.

### 3.2. Organic dyes

Organic dyes are another group of water pollutants of significant concern because many of the dyes used in industry are toxic to aquatic organisms. The wastewater from the dye industry has received attention in recent studies due to the toxicity of some raw materials (aromatic amines) used to produce the dyes. It has been shown that their disposal into the environment has led to severe contamination of significant areas in some countries. Decolourizing these dyes is challenging due to their stability and complex structures. Current technologies used to eliminate these compounds are considered ineffective.^9,13^

**Fig. 10:**
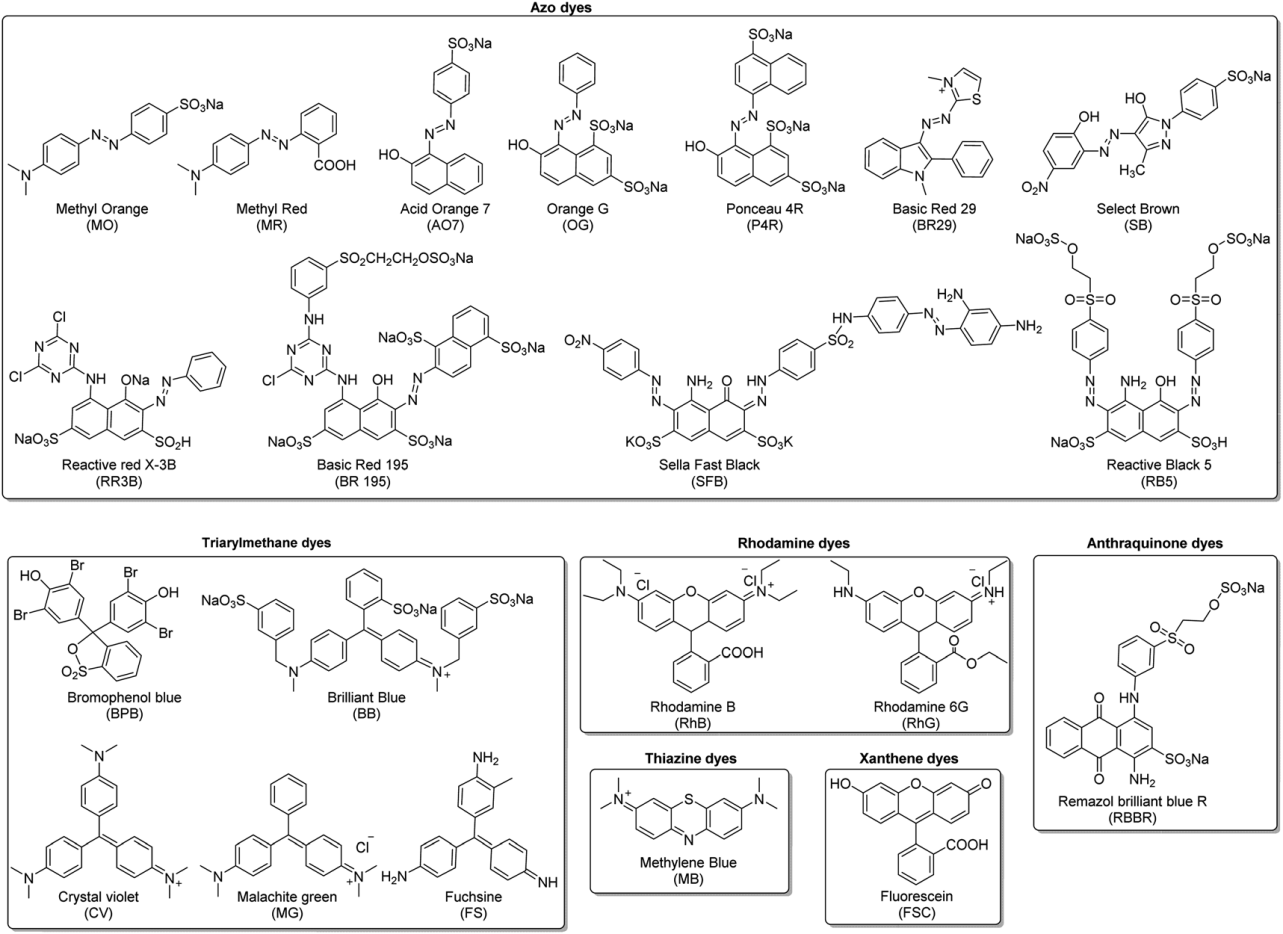
Structure of the main five groups of organic dyes described in this review.

In this review, the organic dyes were divided into six groups (Fig. 10): azo, triarylmethane, rhodamine, thiazine, xanthene, and anthraquinone dyes.

#### 3.2.1. Azo dyes

Azo dyes represent 50% of the world's annual production, making them the largest synthetic dyes group. The name derives from their azo (–N=N–) functional group that can be found with other groups as aromatic rings. Due to their high production, their toxicity has been studied extensively. Their manufacturing was related to several cancer appearances and later proved that some azo dyes were carcinogenic.^133^ For this reason, the appearance of these dyes in wastewater could be considered a considerable health problem. In this review, we reported the studies of the degradation of nine azo dyes (Fig. 10): methyl orange (MO), methyl red (MR), acid orange 7 (AO7), orange G (OG), ponceau 4R (P4R), select brown (SB), sella fast black (SFB), basic red 29 (BR29), and reactive red 195 (RR195).

##### 3.2.1.1. Methyl orange

MO is an unmanageable dye present in Nature that is hard to degrade and, if released into the environment, could cause severe threats to human health.^134^

The photocatalytic degradation of MO was studied by Nyokong and co-workers^100^ in the presence of Zn9 (Fig. 4) immobilized into PSU fibres to avoid aggregation. According to the authors, upon visible light irradiation, the use of PSU fibre (*t*_1/2_ = 135.9 min) seems to reduce the ability of the degradation of MO when compared with the Zn9 (*t*_1/2_ = 81.6 min) by itself (Table 7). Despite having higher half-life times and a lower degradation rate constant, degrading MO with the Zn9/PSU fibres was possible. It is essential to highlight that with an increase in the MO concentration, the higher half-life time for Zn9. The same authors^84^ also used a series of peripherally substituted zinc(ii) and Lu(iii)OAc phthalocyanine complexes 6–12 (Fig. 4) and 29 (Fig. 7) immobilized on PAA and PUR. In aqueous media, it was possible to degrade MO with the photoactive materials Lu7OAc/PS, Lu8OAc/PS, Lu12OAc/PS, and Lu29OAc/PS (Table 7) after 150 min under visible light irradiation (*λ* > 400 nm). These hybrids can degrade the MO slower (*t*_1/2_ = 113.63–182.43 min) than 4-NP and 4-CP. The degradation of MO could be achieved faster with peripherally substituted Zn7/PS fibres (*t*_1/2_ = 113.63 min) when compared with their non-peripheral analogue (Lu29/PS) (*t*_1/2_ = 130.78 min).

**Table 7:** Photophysical parameters for the degradation of MO

MPc	Support	Light	*Φ*_Δ_	Irradiation time (min)	*t*_1/2_ (min)	Efficiency (% or rate)	Recycle	Ref.
Zn9	PSU	Visible light (*λ* > 400 nm)	—	—	135.9	—	—	100
	—				81.6			
Lu29OAc	PS		0.22		130.8			84
Lu7OAc			0.28		113.6			
Lu8OAc			0.17		182.4			
Lu12OAc			0.15		182.4			
Zn34	PS/Ag NPs		0.28		693			135
	PS		0.26		336			
Zn35	PS/Ag NPs		0.25		336			
	PS		0.28		1125			
Zn40	CoFe PA-6		—	60	288.8			142
Zn42					364.7			
Zn40	PA-6				533.1			
Zn42					495.0			
Zn40	α-Fe_2_O_3_				46.2			143
Zn41					42.8			
Without Pc					53.3			
H_2_2	TiO_2_	Visible (*λ* > 550^a^ nm)		180	—	80^b^		87
						50^c^		
Without Pc						10^b^		
Cu3		Visible (*λ* > 400 nm)		60		80		88
H_2_4	Fe–TiO_2_			—	19.8	—		89
	TiO_2_				23.1			
Without Pc					138.6			
Zn4	ZnO	Red light (*λ* = 620 nm)		180	—	35		90
Without Pc						1		
		Visible light (*λ* = 400–800 nm)				60		
Zn4						30		
Zn36	MWCNT	Visible (*λ* > 400 nm)		240		86		136
Co3	MCM-41			120		98	4 cycles (10% loss)	91
Cu4	N-ZIF			90		0^d^	7 cycles (<5% loss)	144
						0.063/min^e^		
						0.055/min^f^		
	PACA HIOB	Visible (*λ* > 420 nm)		20		100 (0.085/min)	–	93

a Performed at 11.0 mW cm^−2^.

b Amorphous TiO_2_.

c P25 TiO_2_; with.

d 0.125.

e 0.25, and.

f 0.5 of (m_Pc_/m_ZIF_) ratio.

**Fig. 11:**
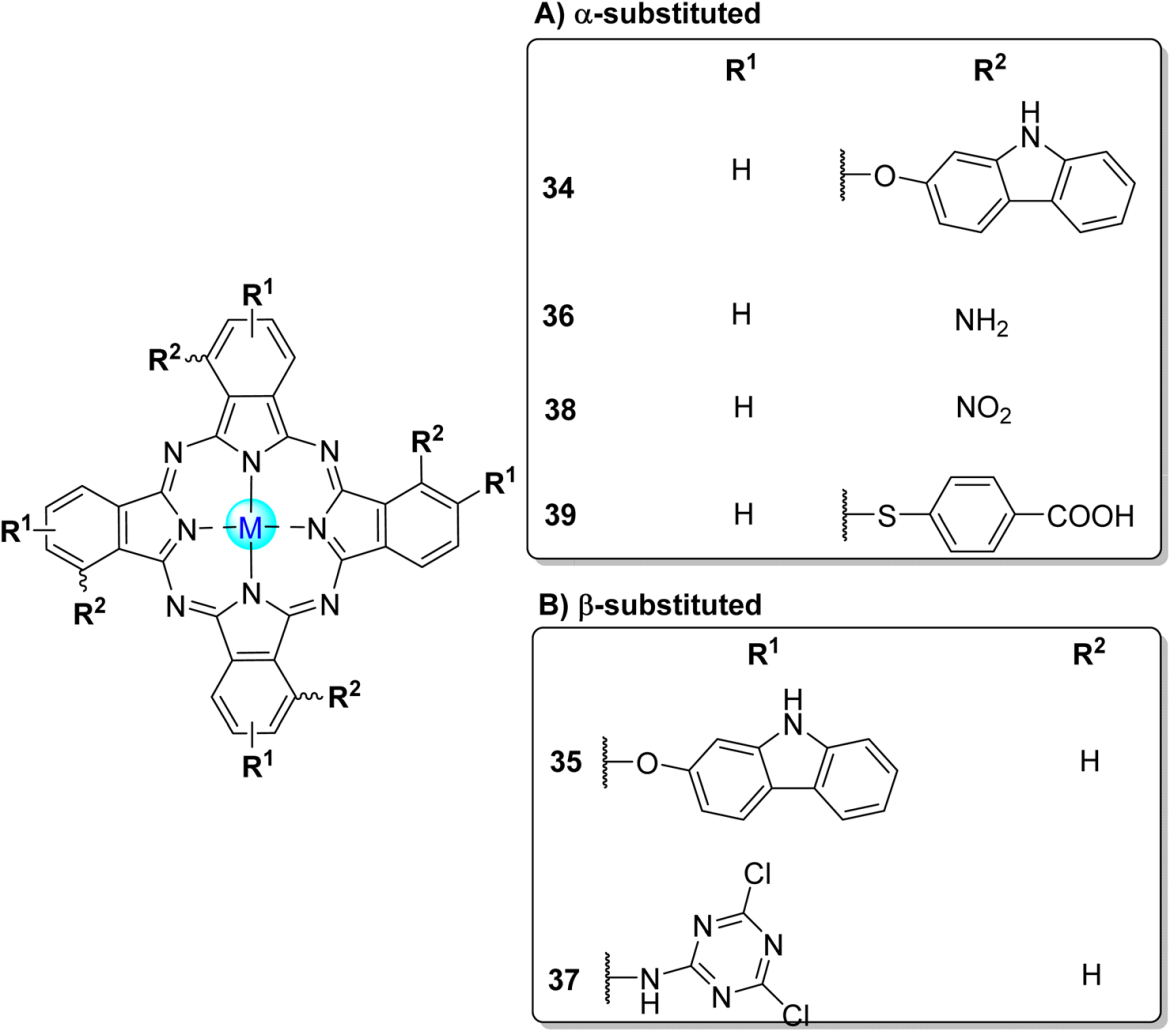
Tetra-substituted phthalocyanines: (A) α-substituted 34,^135–137^36,^37,78,136,138^38,^139^39,^140^ and (B) β-substituted 35,^135^37.^141^

By conjugating silver nanoparticles and fibres, the same authors^135^ degrade MO using α- and β-substituted zinc(ii) phthalocyanines bearing carbazole groups 34 and 35 (Fig. 11), respectively. They linked them to silver nanoparticles, which were further immobilized in PS fibres to be used in the photocatalysis of MO (Table 7). Under visible light irradiation (*λ* > 400 nm), lower half-life times for the degradation of MO were achieved using the α-substituted Zn34/PS relative to the β-substituted Zn35/PS. The photooxidation process increased in the presence of silver nanoparticles when compared with the phthalocyanines.

**Fig. 12:**
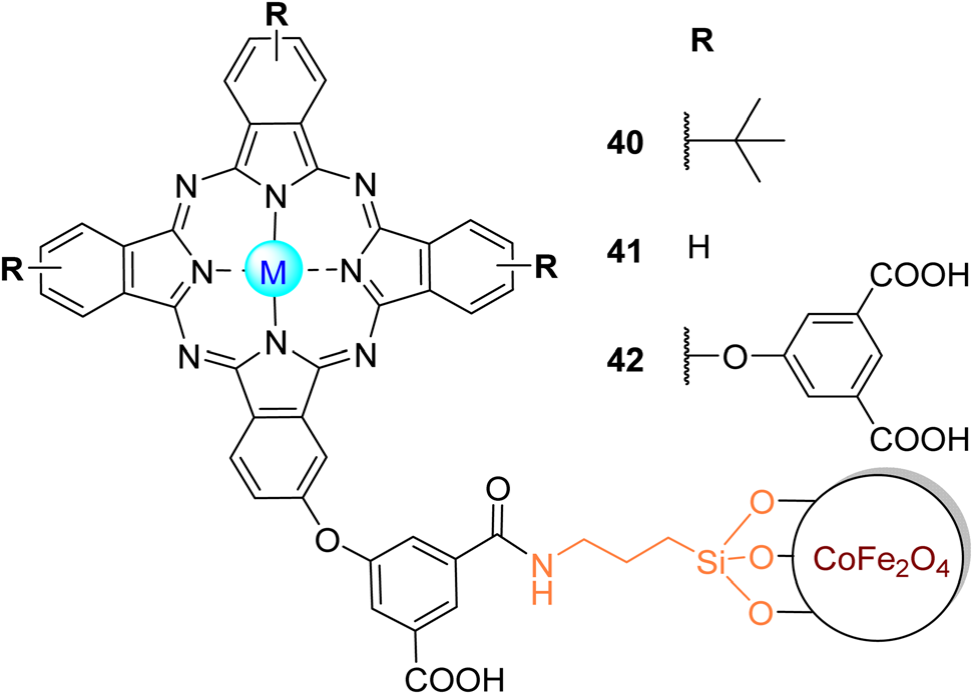
Zn40–Zn42 coupled to CoFe_2_O_4_ MNP used by Nyokong and co-workers^142,143^ for the degradation of MO.

The photooxidation of MO and OG in aqueous media under visible light irradiation (*λ* > 400 nm) with an amine-functionalized cobalt ferrite (CoFe) magnetite magnetic nanoparticle (MNP) conjugated with zinc(ii) phthalocyanines Zn40 and Zn42 (Fig. 12) was studied by Nyokong and co-workers.^142^ These MNPs were embedded into electrospun polyamide-6 (PA-6) fibres for support and catalyst regeneration after the photocatalytic process. The authors compared the photocatalytic activity of CoFe–Zn40/PA-6 and CoFe–Zn42/PA-6 with CoFe/PA-6, Zn40/PA-6, and Zn42/PA-6 (Table 7) for the MO degradation experiment. After 60 min of irradiation, it was possible to achieve better MO degradation rates with the CoFe–Zn40 and CoFe–Zn42 compared with the respective electrospun Pcs and MNPs.

More recently, the same authors^143^ studied the photodegradation of MO under visible light irradiation (*λ* > 400 nm) with fabricated α-Fe_2_O_3_ nanofibres modified with the tetra-substituted phthalocyanine Zn40 (Fig. 12) or mono-substituted phthalocyanine Zn41 (Fig. 12). It was possible to achieve a 50% degradation more rapidly using Zn41–α-Fe_2_O_3_ (*t*_1/2_ = 42.78 min), followed by Zn40–α-Fe_2_O_3_ (*t*_1/2_ = 46.20 min) and α-Fe_2_O_3_ (*t*_1/2_ = 53.31 min) (Table 7). The results show that sensitising α-Fe_2_O_3_ with phthalocyanines reduced the half-life time, increasing the photocatalyst efficiency. By increasing the amount of MO, the half-life time was also increased. After reuse, there was a slight loss of activity as observed by an increase in the half-life times: Zn41–α-Fe_2_O_3_ (*t*_1/2_ = 45.00 min) > Zn40–α-Fe_2_O_3_ (*t*_1/2_ = 48.46 min) > α-Fe_2_O_3_ (*t*_1/2_ = 69.30 min). These authors reported in some of their studies 2-amino-5-(3-hydroxy-4-oxo-cyclohexa-2,5-dienylideneamino)benzene sulfonic acid and poly(catechol) as degradation products of MO.^84,100^

Chen, Zhang, and co-workers^87^ and Wang and co-workers^88^ studied the degradation of MO in aqueous media under visible light irradiation with H_2_2/TiO_2_ amorphous hybrid (Fig. 4) and nanocrystalline anatase TiO_2_ with copper phthalocyanine Cu3 (Fig. 4), respectively (Table 7). In the first study,^87^ it was possible to achieve ∼80% of degradation with H_2_2/TiO_2_ after 180 min of light irradiation. These results are much higher when compared with H_2_4/P25 (50% within 180 min). At the same time, there was no degradation using pristine TiO_2_ and H_2_2/SiO_2_. In the second study,^88^ 80% of MO could be degraded with the hybrid Cu3/TiO_2_ within 60 min. The samples were heated at different temperatures, and the photocatalytic activity was again evaluated. After increasing the temperature, the dimeric form of Cu3 was disaggregated and vaporized or desorbed, and the amount of immobilized monomer was much higher when compared with the dimer. The excited state of the monomer has a much higher half-life than the aggregates, favouring the electron injection process. Gharagozlou and co-workers^89^ evaluated the photooxidation of MO after doping TiO_2_ with Fe and preparing H_2_4 (Fig. 4)–Fe-doped TiO_2_ (H_2_4/Fe–TiO_2_) with different Fe doping content (0, 0.05, 0.5, and 3.0 mol% Fe). As expected, the Fe amount curiously influences the activity of the TiO_2_ composites. Under visible light irradiation (*λ* > 400 nm) and in the presence of H_2_4/Fe–TiO_2_ with different doping amounts of Fe, it was possible to observe the photocatalytic activity on MO (Table 7). The results showed that the degradation of MO is more effective using H_2_4/Fe–TiO_2_ (*t*_1/2_ = 19.8 min) than using undoped H_2_4/TiO_2_ (*t*_1/2_ = 23.1 min) and bare TiO_2_ (*t*_1/2_ = 138.6 min). These results can be due to electron–hole pairs' formation and electrons' transference from H_2_4 to TiO_2_. Also, according to the field theory, Fe^2+^ is unstable compared to Fe^3+^, and a release of a trapped electron occurs, returning to the Fe^3+^ form. Initially, the photocatalytic degradation of MO increased with the increase of the doping amount of Fe on the composite, reaching a maximum activity of 0.5% and then decreasing with higher amounts of Fe. This activity can be explained by the fact that above 0.5 mol% of Fe, the Fe^3+^ ions act as recombination centres for photo-generated electrons and holes, decreasing photocatalytic activity.

Regarding zinc oxide, Ahmed, Pal, and co-workers^90^ developed a study of the degradation of MO using a hybrid containing zinc oxide nanorods and Zn4 (Fig. 4) (Zn4–ZnO) functionalized with two carboxyl groups of a tartrate molecule by interaction with the metallic zinc. According to Table 7, the authors performed the photocatalytic studies under red light (*λ* = 620 nm) and white light irradiation (*λ* > 365 nm). It was possible to degrade MO more efficiently with the hybrid material (35%) compared to bare ZnO (1%) after 180 min of red light irradiation. The best results were obtained under white light irradiation (*λ* > 365 nm), where the nanohybrid material Zn4–ZnO could achieve about 60% photocatalytic efficiency.

Xu, Li, and co-workers^136^ studied the degradation of MO in an aqueous solution under visible light irradiation (*λ* > 400 nm) by using substituted zinc(ii) aminophthalocyanine Zn36 (Fig. 11) supported by functionalized multi-walled carbon nanotubes (MWCNTs). The authors proved by Raman spectroscopy that the covalent attachment of Zn36 to MWCNTs and the π–π interactions between these two entities induce the disaggregation of the Zn36. After 4 h, 86% of MO was degraded (Table 7). The authors also assessed these photocatalysts' ability to degrade Rhodamine B (RhB).

Liu, Zhao, and co-workers^91^ studied the degradation of MO with Co4 (Fig. 4) immobilized onto MCM-41 under visible light irradiation. The authors established that either less or an excess amount of photocatalyst in the reaction solution impacted the degradation of MO. So, a low photocatalyst amount was insufficient to interact with all the MO molecules. However, an excess photocatalyst amount of Co3 caused a shielding effect in light penetration. It was possible to achieve 98% MO degradation after 120 min of light irradiation with an optimal catalyst amount of 0.2 mg mL^−1^ (Table 7). After 4 cycles, it was possible to maintain a degradation rate of 90%.

Dabiri and co-workers^144^ developed a study where they could degrade MO with nitrogen-doped carbon photocatalyst based on the carbonization of Cu4 (Fig. 4) on/in zeolitic imidazolate framework-8 (ZIF-8) hybrid–Cu4/N-PC. The authors could obtain Cu4_0.125_/N-PC, Cu4_0.25_/N-PC, and Cu4_0.5_/N-PC with different weight ratios were obtained. After 90 min of visible light irradiation (*λ* > 400 nm), there was no degradation of MO with Cu4_0.125_/N-PC (Table 7). On the other hand, in the presence of H_2_O_2_, the authors could degrade MO when using Cu4_0.25_/N-PC (0.0627 min^−1^) followed by Cu4_0.5_/N-PC (0.0551 min^−1^). Also, after seven cycles, there was a loss of 4% in the degradation rate of MO. These studies were extended to RhB.

More recently, Zang, Sun, Zhang, and co-workers^93^ studied the degradation of MO in an aqueous solution under visible light irradiation (*λ* > 420 nm) by using the Cu4 (Fig. 4) supported by poly(acrylamide-acrylic acid copolymer) hydrogel inverse opal beads (PACA HIOB). The authors assembled silica microspheres as sacrificial templates and infused monomers within the pores for UV polymerisation to form the PACA HIOBs. After 20 min of light irradiation, there was a complete degradation of MO with a degradation rate of 0.0850 min^−1^ (Table 7). The authors also assessed these photocatalysts' ability to degrade other anionic dyes as Reactive red X-3B (RR3B), reactive black 5 (RB5), Remazol brilliant blue R (RBBR) and cationic dyes as RhB and Malachite green (MG). According to the authors, the degradation kinetics of the anionic dyes, except for RBBR, were faster than those of cationic dyes. The work of Zang, Sun, Zhang, and co-workers^93^ was better than other hydrogel-based photocatalysts because the hydrogel-base inverse opal scaffold can increase light absorption, catalytically active sites, reduce charge transfer resistance and inhibit the recombination of photogenerated electron–hole pairs. Regarding MO, the authors did not study the degradation products or the recyclability of the photocatalyst.

##### 3.2.1.2. Methyl red

MR is an azo dye that appears in textiles and other commercial products. This dye can cause eye and skin sensitization and digestive tract irritation when swallowed, and thus, it is important to remove it from wastewater.^145^

**Fig. 13:**
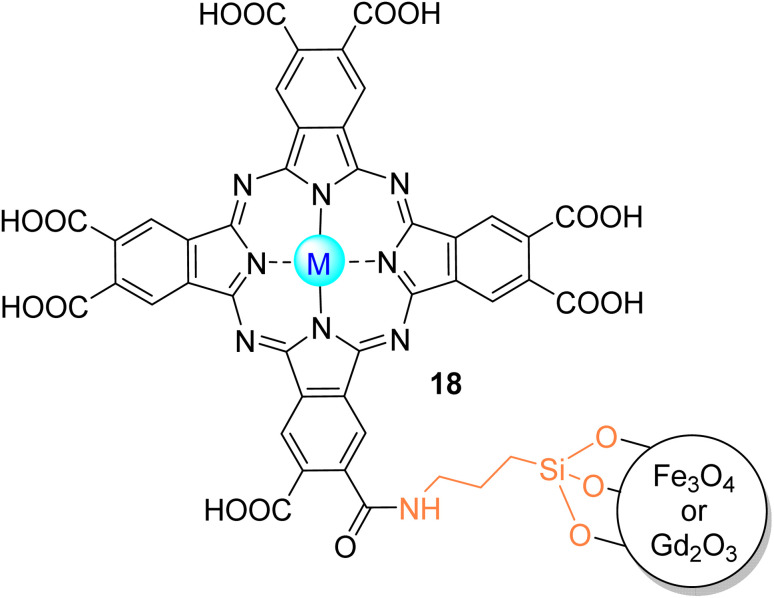
In(iii)18Cl or Zn(ii)18 covalently bonded to MNP of Fe_3_O_4_ or Gd_2_O_3_ used by Nyokong and co-workers^103^ for the degradation of MR.

Nyokong and co-workers^103^ studied the degradation of MR in aqueous media (pH = 7.4) with a covalently linked In18Cl to MNP already functionalized with (3-aminopropyl)triethoxysilane (Fig. 13). The use of MNP allows the catalyst to be recovered several times with magnetic separation. Furthermore, In18Cl and its conjugate with MNP have been embedded in PAN electrospun fibres. Under visible light irradiation (*λ* > 400 nm), it was possible to degrade more efficiently the MR with the MNP–In18Cl/PAN, given the lower time of half-life (*t*_1/2_ = 29.5 min) and higher degradation rate (*k*_obs_ = 0.0235 min^−1^). After the reuse of the catalyst, there was a slight decrease in the MR degradation rate and an increase in the half-life time. In this study, the authors did not perform stability studies, but they mentioned that the conjugates are stable because, during the singlet oxygen generation assays, the Q bands of the compounds remained intact on their absorption spectra.

##### 3.2.1.3. Acid orange 7 (or orange II)

**Fig. 14:**
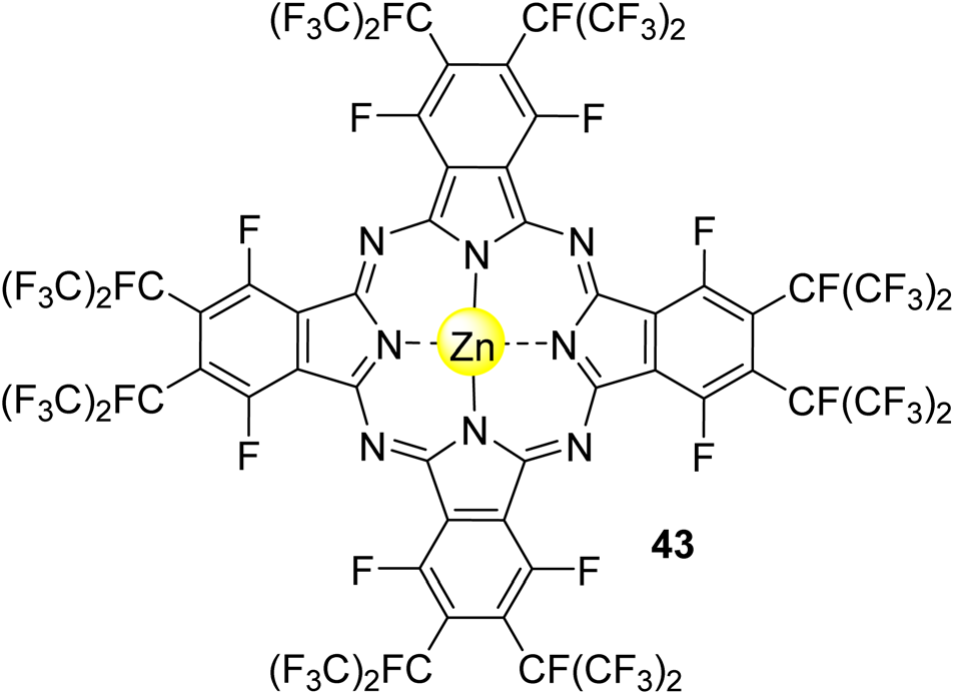
Zinc(ii) perfluoroalkyl perfluorophthalocyanine 43 used by Nowakowska and co-workers^147^ for the degradation of AO7.

Acid orange 7 (AO7), also known as orange II, is an azo dye extensively used for dyeing wool. This dye is non-biodegradable, and for that reason, its removal from wastewater is needed.^146^ Nowakowska and co-workers^147^ studied its degradation in aqueous media under visible light irradiation (*λ* = 400–550 nm) using the fluorinated phthalocyanine Zn43 (Fig. 14) immobilized into bentonite as the photocatalyst. A degradation of 80% was observed after 200 min of light irradiation. Reusability studies showed that the catalyst could be reused four times without significant activity loss (15%).

The degradation of AO7 in aqueous media using visible light irradiation was also evaluated by Schneider and co-workers^104^ in the presence of Cu18 (Fig. 6) immobilized on g-C_3_N_4_. A maximum degradation rate of 95% was achieved after 180 min of light irradiation with 0.5 wt% of Cu18. Recyclability studies of the catalyst revealed a slight decrease in activity (5% loss) after five cycles. Qu and co-worker^141^ selected AO7 as a target pollutant for their decomposition, evaluating the use of cobalt(ii) phthalocyanine Co37 (Fig. 11) immobilized on activated carbon fibres. The AO7 removal was enhanced using Co37/carbon fibres as a catalyst and UV light irradiation (*λ* = 365 nm). In fact, within 120 min of irradiation, there was a 23% enhancement in the dye removal. The best photocatalytic rate (47%) was achieved using a 2 g L^−1^ catalyst concentration. The authors also assessed the influence of H_2_O_2_ addition, where it was possible to achieve 99% of the photodegradation rate within 60 min of irradiation with a concentration of H_2_O_2_ of 12.5 mmol L^−1^. After four cycles, the photocatalytic rate was still above 92%, meaning no significant activity loss occurred.

##### 3.2.1.4. Orange G

Orange G (OG) is non-biodegradable, toxic, and potentially carcinogenic.^148^ The photooxidation of OG in aqueous media under visible light irradiation (*λ* > 400 nm) using Zn18 immobilized on the surface of magnetite magnetic nanoparticles (Fig. 13) was studied by Antunes and co-workers.^105^ According to the authors, it was possible to degrade OG more efficiently using the nanoparticle (lower half-life time (*t*_1/2_ = 131 min) and rate (*k*_obs_ = 0.0053 min^−1^)) when compared with the phthalocyanine by itself (*t*_1/2_ = 157 min, *k*_obs_ = 0.044 min^−1^). After one reuse, the catalyst remained stable towards OG photodegradation with a slight decrease in the degradation rates (*k*_obs_ = 0.0049 min^−1^) and an increase in the half-life time (*t*_1/2_ = 141 min). Also, for the degradation of orange G, Nyokong and co-workers^95,142^ prepared conjugates of octa-substituted carboxylic zinc(ii) phthalocyanine Zn18 with Gd_2_O_3_ MNPs with PA-6 fibres (Fig. 13) and an amine-functionalized CoFe MNP conjugated with zinc(ii) phthalocyanines Zn40 and Zn42 (Fig. 12). In their first study,^142^ after 60 min under visible light irradiation (*λ* > 400 nm), the electrospun Pcs CoFe–Zn40 (*t*_1/2_ = 41.50 min) and CoFe–Zn42 (*t*_1/2_ = 44.42 min) were the most effective photocatalysts compared with the respective Pc-MNP conjugates (*t*_1/2_ = 51.33 and 52.11 min, respectively). In their second study,^95^ OG was easily degraded (∼90%) after 30 min of visible light irradiation (*λ* > 400 nm) in the presence of the composite Zn2–MNPs/PA-6 fibres. More recently, Yilmaz and co-workers^140^ could degrade OG in aqueous media with a tetra-substituted thiocarboxylic zinc(ii) phthalocyanine 39 (Fig. 11) immobilized onto Au@SiO_2_. After 25 min of visible light irradiation (*λ* > 400 nm), there was an 80% of OG degradation. After 3 cycles, half of the photocatalytic activity was lost.

##### 3.2.1.5. Ponceau 4R (acid red 18), select brown (acid brown 98) and sella fast black (acid black 210)

Textile dyes like P4R, SB and SFB are intended to resist degradation, chemically stable, non-biodegradable, toxic, and carcinogenic.^62,86^ Machado and co-workers^62^ studied the degradation of P4R under visible light irradiation with f Zn4 (Fig. 4) functionalized on TiO_2_ (Zn4/TiO_2_ 1.6 wt%). According to the authors, the as-prepared composite showed a better performance when compared with bare TiO_2_ reaching 50% of P4R degradation within 120 min. For the photodegradation of P4R, SB and SFB, Raducan and co-workers^86^ used Cu3 (Fig. 4)–TiO_2_, Cu4–TiO_2_ (Fig. 4), and Cu25–TiO_2_ (Fig. 6). The different photocatalytic activities in the aqueous media of these nanocomposites are presented in Table 8. Regarding the recyclability studies, the authors selected Cu3–TiO_2_ and brilliant blue as a dye.

**Table 8:** Photocatalytic efficiencies of Cu3, Cu4, and Cu25 immobilized on TiO_2_.^86^

Pollutant/MPc	No	Cu3	Cu4	Cu25
P4R (%)	20	20	20	20
SB (%)	42	60	42	60
SFB (%)	8	0	5	18

##### 3.2.1.6. Reactive black 5

RB5 is most widely used for dying cotton and other cellulose fibres. There is a huge consumption of this dye in industry.^149^ Zang, Sun, Zhang, and co-workers studied^93^ the degradation of RB5 in an aqueous solution under visible light irradiation (*λ* > 420 nm) by using the Cu4 (Fig. 4) supported by PACA HIOB. After 20 min of light irradiation, there was a complete degradation of RB5 with a degradation rate of 0.0772/min. The authors also assessed these photocatalysts' ability to degrade other anionic dyes such as Reactive red X-3B (RR3B), RBBR and cationic dyes such as RhB and Malachite green (MG). Regarding RB5, the authors did not study the products of degradation or recyclability.

##### 3.2.1.7. Basic red 29 and reactive red 195

Azo dyes like BR29 and RR195 are resistant to biodegradation under aerobic conditions, whereas anaerobic treatment could be applied successfully. However, anaerobic processes cannot be used in wastewater treatments because the breakdown of these dyes can form aromatic amines, which are considered more toxic than the dyes themselves.^150,151^

The degradation of BR29 and RR195 using Fe4/AT-PAN and Fe4/PAN (Fig. 6) was studied in aqueous media under visible light irradiation by Han, Zhao, and co-workers.^63^ In the presence of H_2_O_2_, it was possible to have a complete degradation of RR195 with and without light irradiation with the AT-PAN as a catalyst. On the other hand, only 20% of RR195 could be degraded either in the presence or absence of light, showing no difference in using an irradiation system for both dyes. Regarding the PAN fibres catalyst, the influence of visible light is noticeable, with an increase in the degradation rate of 50–90% for BR29 and 40–80% for RR195, respectively. The degradation products of these dyes were not identified, and the authors did not perform stability and photodecomposition studies of the catalysts.

##### 3.2.1.8. Reactive red X-3B

RR3B is an azo dye used in fabric dyeing and represents almost 60% of the total dyes used in the dyeing industry. Due to their toxicity, its discharge into the water will threaten aquatic and human organisms.^152^ Zang, Sun, Zhang, and co-workers^93^ studied the degradation of RR3B in an aqueous solution under visible light irradiation (*λ* > 420 nm) by using the Cu4 (Fig. 4) supported by PACA HIOBs. After 15 min of light irradiation, there was a complete degradation of RR3B with a degradation rate of 0.1218 min^−1^. After five cycles, the degradation efficiency was still 98.1%. The authors also assessed these photocatalysts' ability to degrade other anionic dyes, such as RBBR and cationic dyes, such as RhB and Malachite green (MG). Regarding RR3B, the authors did not study the degradation products.

#### 3.2.2. Triarylmethane dyes

Triarylmethanes are one of the most used types of dyes in the textile industry. These dyes are considered toxic and carcinogenic. There are some physicochemical methods that are already used to degrade these dyes, but they are considered inefficient and expensive and can produce by-products that can be more toxic than the dyes.^153^ In this section, crystal violet (CV), bromophenol blue (BPB), brilliant blue (BB), and fuchsine (FS) are used as model pollutants.

##### 3.2.2.1. Crystal violet

**Fig. 15:**
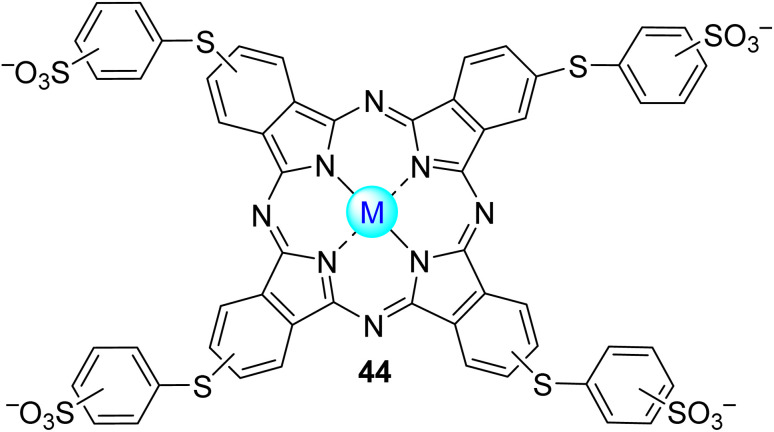
Phthalocyanine 44 used by Mohamed, Youssef and co-workers^155,157^ for the degradation of CV and BPB.

CV is considered carcinogenic with acute cytotoxicity to some animals and plants. The inadequate discharge into the environment can cause some serious health problems.^154^ For the degradation of CV, Mohamed and co-workers^155^ used a composite based on ZnO and Cu44 (Fig. 15) and a solar simulator as an irradiation source. A complete degradation was achieved with the composite Cu44/ZnO in the presence of H_2_O_2_ (80% in the absence of H_2_O_2_) after 40 min of irradiation in aqueous conditions (pH = 5). These results showed an enhancement of the photocatalytic activity compared with bare ZnO and Cu44. There was a decrease of 10% in the photocatalytic degradation of CV after three cycles of the experiment.

##### 3.2.2.2. Bromophenol blue

BPB is a triarylmethane dye considered hazardous for all living beings, negatively affecting photosynthesis in aquatic organisms. It is considered carcinogenic, mutagenic, and allergenic.^156^ For the BPB degradation in aqueous media, Mohamed and Youssef^157^ used a nickel(ii) phthalocyanine Ni44 (Fig. 15) immobilized on TiO_2_ nanoparticles and visible light irradiation (*λ* > 400 nm). The characterization of the resulting material showed that Ni44 is anchored to the surface of TiO_2_ through SO_2_–O–TiO_2_ bonds. It was possible to achieve complete degradation of BPB within 50 min. No significant loss of activity was observed after 3 cycles.

##### 3.2.2.3. Brilliant blue (acid blue 9) and fuchsine (basic violet 14)

BB was a popular colorant in the textile industry and used as a common food additive. Due to its toxic effects on humans and animals, it was banned. It is reported to be carcinogenic, causing reproductive and neurological disorders, allergies, and trouble breathing.^158^ FS is also carcinogenic and mutagenic and has a very slow degradation in Nature.^159^

Raducan and co-workers^86^ studied the degradation of BB and FS in aqueous media using Cu3–TiO_2_ (Fig. 4), Cu4–TiO_2_ (Fig. 4), and Cu25–TiO_2_ (Table 9). Regarding the recyclability studies, the authors selected Cu3–TiO_2_ as the catalyst and BB as the pollutant. The results showed that after 10 cycles a photodegradation rate of 20% was achieved for the selected dye.

**Table 9:** Cu3–, Cu4–, and Cu25–TiO_2_ degradation efficiencies against brilliant blue and Fuchsine

Pollutant/MPc	No	Cu3	Cu4	Cu
BB (%)	10	10	22	22
FS(%)	38	38	58	40

##### 3.2.2.4. Malachite green

MG is an extensively used biocide in aquaculture with genotoxic and carcinogenic properties.^160^ So, Zang, Sun, Zhang and co-workers^93^ studied the degradation of MG in an aqueous solution under visible light irradiation (*λ* > 420 nm) by using the Cu4 (Fig. 4) supported by PACA HIOBs. MG was degraded entirely after 40 min of light irradiation with a degradation rate of 0.0601 min^−1^. The authors also assessed these photocatalysts' ability to degrade other anionic dyes, such as RBBR and cationic dyes, such as RhB. Regarding MG, the authors did not study the degradation products or the recyclability of the photocatalyst.

#### 3.2.3. Rhodamine dyes

Rhodamines are used for dye laser materials, but they are considered the most toxic dyes in the textile industry because of their high stability and non-biodegradable properties. This review will discuss the photodegradation of Rhodamine B (RhB) and Rhodamine G (RhG). Both dyes are classified as carcinogenic and neurotoxic, causing respiratory tract infections. When inhaled and ingested, they can cause liver and thyroid damage and eye and skin irritations. The traditional methods to remove these dyes require a complex treatment and present several disadvantages, such as high energy consumption and the formation of toxic by-products.^161,162^

##### 3.2.3.1. Rhodamine B

RhB is a dye used in the textile industry and in food processing. However, since it has been classified as carcinogenic, it has been forbidden from being used in food processing for decades. Several strategies exist to obtain MPcs with high floating properties, like making phthalocyanine super-hydrophobic. So, for the degradation of RhB in aqueous media under visible light irradiation (*λ* > 400 nm), Shao, Chen, and co-workers^64^ used a hierarchical nanostructure with a hollow interior space composed of zinc(ii) phthalocyanine Zn4 (Fig. 4). Within 660 min, 89% of the RhB was photodegraded in the presence of the hierarchical tubular structure (Table 10).

**Table 10:** Photocatalytic parameters for the degradation of RhB

MPc	Support	Light	Efficiency (%)	Irradiation time (min)	Recycle	Ref.
Zn4	—	Visible (*λ* > 400 nm)	89	660	—	64
Cu45–CMP			50	150		65
Zn45–CMP			50			
Co5			80			
Co45–CMP			100	30	4 cycles (10% loss)	
Co46	Chitosan	UV	99	60	5 cycles (40% loss)	164
Fe4	PAN	Visible (*λ* > 400 nm)	100	60	5 cycles (12% loss)	63
	AT-PAN					
Zn4	TiO_2_		70	—	7 cycles (10% loss)	66
—	P-25		10		—	
Zn4	TiO_2_	Sunlight	90			
—	P-25		80			
Co4	TiO_2_	Solar	73	90		34
Fe4			88			
No			78			
Zn52	TiO_2_	Visible (*λ* > 400 nm)	90	180		165
Cu5			87	240		67
Fe5	TiO_2_/carbon fibres	Visible (*λ* = 400–700 nm)	91	180	3 cycles (<5% loss)	68
	No		35			
Without Pc	P25		27			
Without Pc	Carbon fibres		46			
Cu4	rGO	Visible (*λ* > 400 nm)	96	210	—	69
Zn4	MWCNTs		93	60		70
	No		54			
No	MWCNTs		4			
Zn36			88	240	3 cycles (<5% loss)	136
No			19			
			14	120	—	139
Zn38			93			
	No		54			
Cu47	Perylene diimide		80	180		166
Sn4	NiWO_4_	UV	71 (1 wt%)	120		71
			48 (3 wt%)			
			49 (2 wt%)			
	No		37			
No	NiWO_4_		30			
Cu5	BiOCl/PAN	UV-visible	80	180	3 cycles (0% loss)	167
Fe4	AT-PAN	Visible (*λ* > 400 nm)	100	80	5 cycles (0% loss)	73
Zn16	g-C_3_N_4_/PAN		98	120	—	56
		Solar	100	180	5 cycles (<5% loss)	
Co4	GR–ZnO			150	—	74
No				140		
Cu4^a^	N-ZIF	Visible (*λ* > 400 nm)	0.0627 min^−1^	90	7 cycles (<5% loss)	144
Mn3	TiO_2_–SiO_2_		100	240	—	168
Fe18	AT/Fe_3_O_4_		96	300		106
Cu4	PACA HIOBs	Visible (*λ* > 420 nm)	40	100 0.0685 min^−1^		93

a With 0.25 mg of photocatalyst amount.

**Fig. 16:**
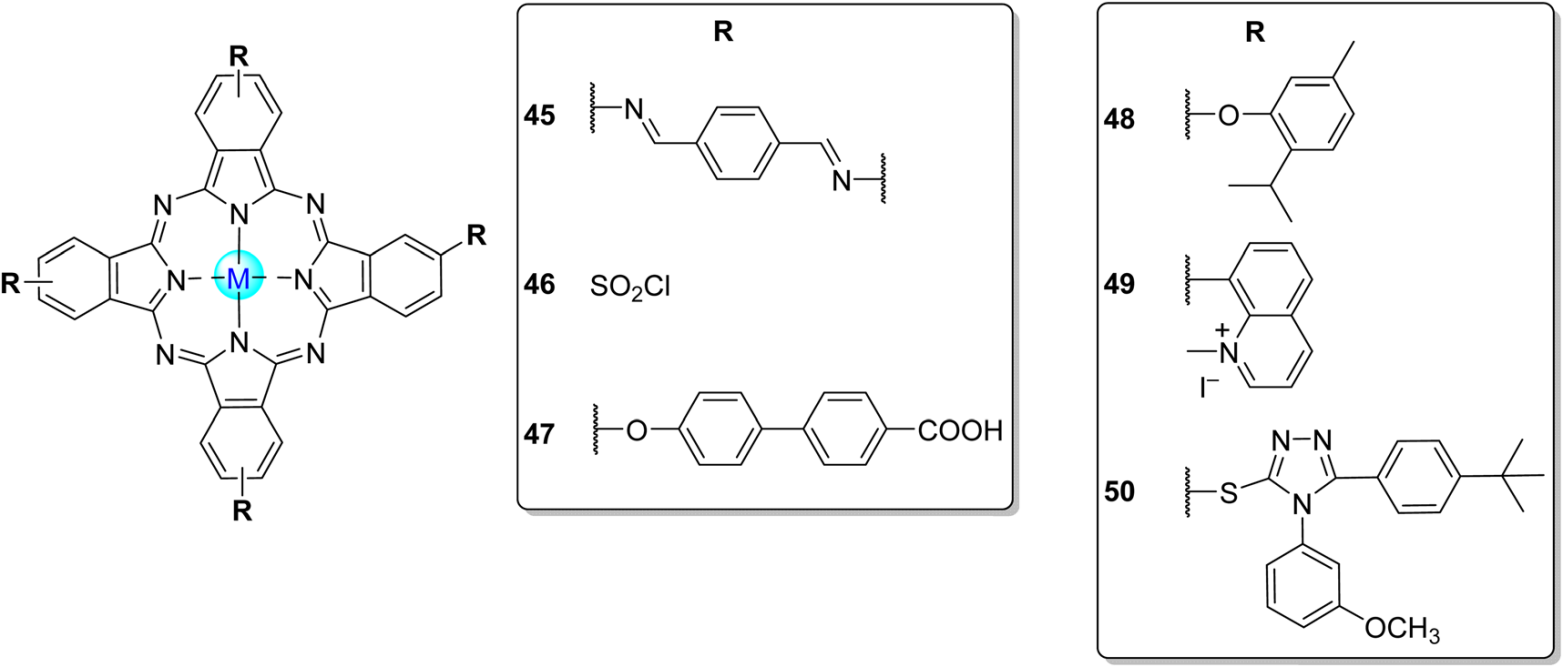
Tetra-β-substituted phthalocyanines 45–50.^65,164,166,169–171^

Conjugated microporous polymers (CMP) are crosslinked polymers that merge porosity and extended π-conjugated systems. Their building blocks are spatially segregated to suppress the π–π interaction. They have a high surface area, rigid backbone, and high thermal stability.^163^ So, Duan and co-workers^65^ studied in aqueous media the degradation of RhB in the presence of H_2_O_2_ with a series of metallophthalocyanine-based microporous polymers M45–CMP (M = Co(ii), Cu(ii), or Zn(ii), Fig. 16) and visible light irradiation (*λ* > 400 nm, Table 10).

The authors discovered that the efficiency of the photocatalytic activity on RhB was improved when the amount of catalyst increased, but an excessive amount of catalyst proved useless for the system. Also, when comparing the photocatalytic activity of Co45–CMP with the monomer Co5, the polymer Co45–CMP showed an enhanced photocatalytic activity (∼100% after 30 min of irradiation *vs.* 80% after 150 min of irradiation). After discovering the ideal conditions, the three photocatalysts' ability to degrade RhB was accessed under the same conditions (15 mg of M45, in H_2_O_2_). It was possible to complete the degradation of RhB after 30 min with Co45–CMP (∼100% of degradation after 30 min of irradiation). On the other hand, it was only possible to degrade 50% of RhB with Cu45–CMP and Zn45–CMP after 180 min of light irradiation. The best photocatalytic rate with Co45–CMP could be maintained without a significant decrease after four experiments. The authors mentioned that these results indicate that the MPc-CMPs can potentially apply to the environmental purification of organic pollutants in industrial wastewater.

**Fig. 17:**
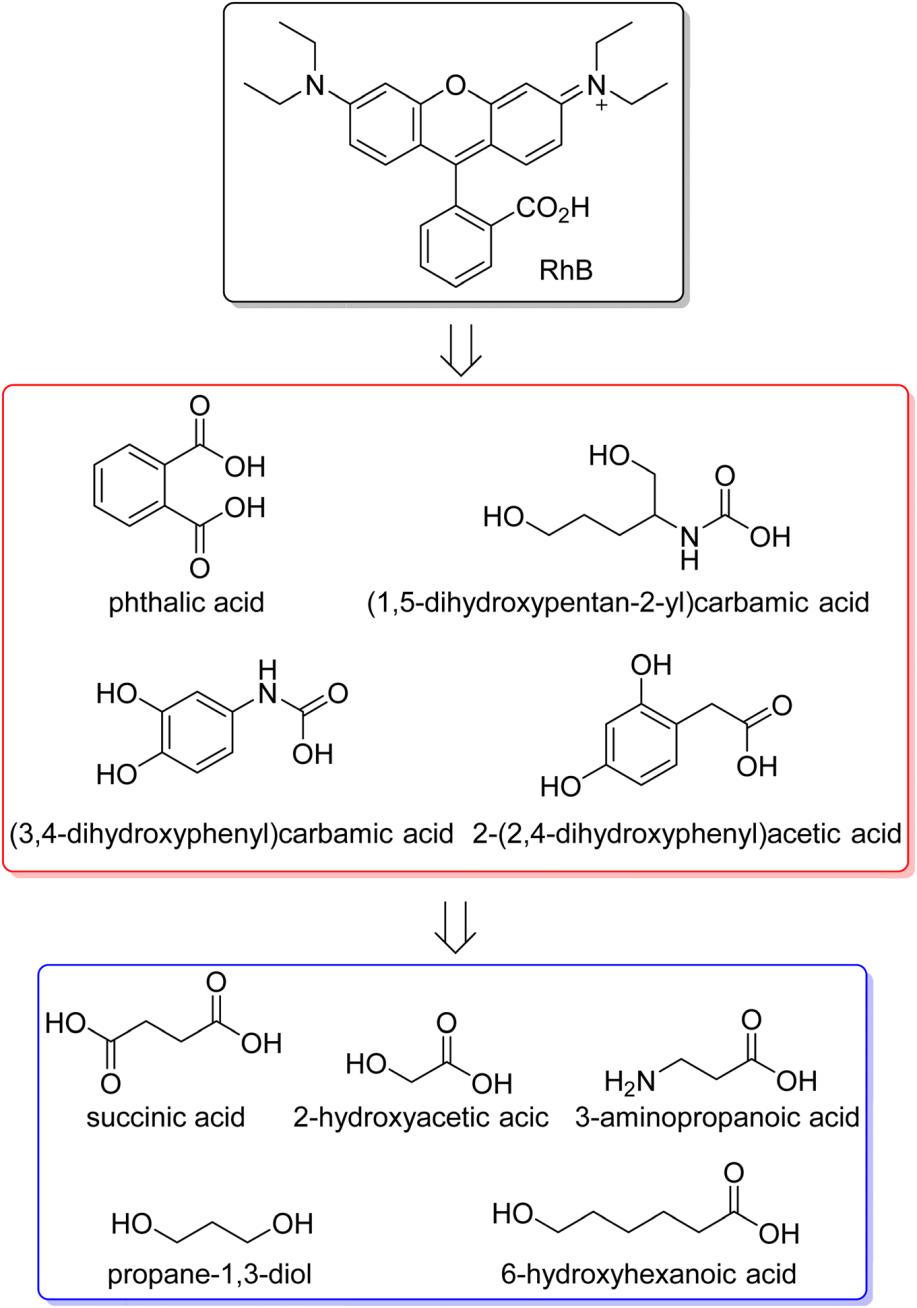
Degradation products of RhB reported by Han, Zhao, and co-workers.^63^

Another study of the RhB degradation in an aqueous media was performed by Wang and co-workers^164^ using a chitosan-supported cobalt(ii) phthalocyanine Co46 (Fig. 16) membrane under UV light irradiation. According to the authors, chitosan is linked to the phthalocyanine Co46 through a sulfonamide bond established between the chlorosulfonyl groups and the amino groups of the chitosan. There was a 99% degradation of RhB with the polymer after 60 min (Table 10). After five experiments, retaining 60% of the degradation rate was possible. Han, Zhao, and co-workers^63^ studied the photooxidation for RhB in aqueous media using iron(ii) phthalocyanine Fe4 (Fig. 4) immobilized onto copper(ii)–amidoximated polyacrylonitrile fibre (AT-PAN), visible light, and H_2_O_2_. The amidoxime groups aided in the anchoring process of Fe4 to the PAN fibre through coordination interaction. The authors prepared two different catalysts: one containing unmodified PAN fibres and the other containing the AT-PAN. When using either catalyst, complete degradation of RhB was achieved in the presence of H_2_O_2_ within 60 min (Table 10). After 5 cycles, it was only observed a 12% decrease in the photodegradation rate. Regarding the degradation products, they reported the ones presented in Fig. 17. At the end of the photocatalytic assay, the authors could find carboxylic acids and alcohol as the final products of RhB.

For the degradation of the same pollutant in aqueous media, Xin and co-workers^66^ and Varghese and co-workers^34^ used TiO_2_ sensitized with Zn4 (Zn4–TiO_2_, Fig. 4), Fe4 or Co4 and visible (*λ* > 400 nm) or simulated solar light. In the first study,^66^ the authors prepared TiO_2_ nanoparticles through a hydrothermal method with posterior impregnation of the Zn4. According to the results, the Zn4–TiO_2_ has an extended absorption band into the visible region compared to bare TiO_2_ (Degussa P25). This photophysical feature induces a higher photocatalytic activity under the simulated solar light and visible light (*λ* > 400 nm) when compared with bare TiO_2_. As expected, this difference is more pronounced under visible light than simulated solar light (Table 10). In the second study,^34^ under solar irradiation and using Fe4/TiO_2_, Co4/TiO_2_, and bare TiO_2_, it was possible to degrade 88%, 73%, and 78%, respectively (Table 10). Despite promising photocatalytic activities, evaluating the stability of compounds within several cycles would be essential.

**Fig. 18:**
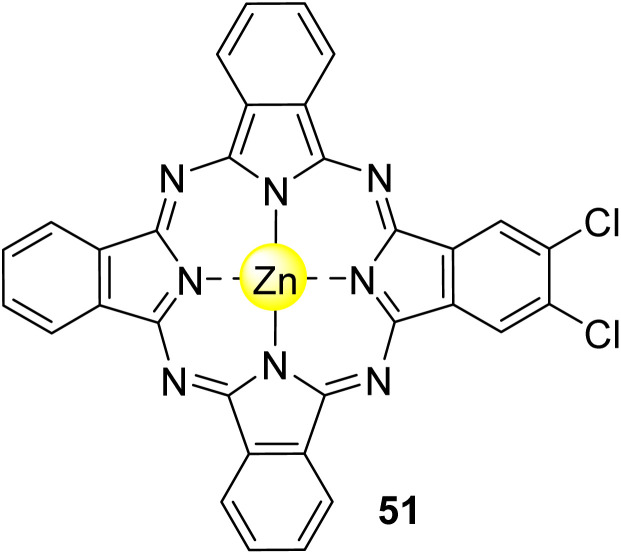
β,β′-Dichlorinated zinc(ii) phthalocyanine 51 used by Sosa-Sánchez and co-workers^165^ for the degradation of RhB.

Still studying the degradation of RhB in aqueous media, Sosa-Sánchez and co-worker^165^ used a hybrid photocatalyst with the non-symmetrical zinc(ii) phthalocyanine Zn51 (Fig. 18) and visible light. This hybrid was compared to the individual molecules (TiO_2_ and Zn51) to evaluate the sensitization effect on their photocatalytic efficiency. It was observed that an improvement in the photocatalytic degradation of RhB occurred after sensitization of the TiO_2_. After 180 min, there was a decrease of 90% in the concentration of RhB (Table 10).

Shao, Mu, and co-workers^67,68^ accessed the photooxidation of RhB using photocatalysts based on hierarchical nanostructures with Cu5 (Fig. 4) immobilized on electrospun TiO_2_ nanofibers and the loading of Fe5 (Fig. 4) nanosheets on one-dimensional carbon nanofibers. In the first study,^67^ the photocatalytic degradation of RhB under visible light irradiation (*λ* > 400 nm) showed that the 1 : 50 (molar ration) Cu5/TiO_2_ nanofibers could achieve a degradation rate of 38% after 4 h, much higher than bare TiO_2_ nanofibers (Table 10). After increasing the molar ratio to 1 : 20, 87% degradation was achieved within 240 min. In the second study,^68^ Fe5 nanosheets were uniformly distributed on the surface of each fibre without enhancing an aggregation phenomenon, offering a high surface area of the nanosheets. In the synthetic approach, the Fe5 nanosheets were grown onto the surface of the carbon fibres, which was proved by scanning electron microscopy (SEM) and transmission electron microscopy (TEM). In the photocatalytic studies, after 180 min under visible light irradiation (*λ* = 400 – 700 nm), the Fe5/carbon fibres could achieve a 91% degradation rate. It is important to highlight that pure Degussa P25 (TiO_2_ used as a comparison), Fe5, and carbon fibres did not exhibit high photocatalytic rates, reaching 27%, 35%, and 46% within the same irradiation time. The Fe5/carbon fibres could be reused (after separation by sedimentation process) for up to three cycles, maintaining the same photocatalytic activity. The authors also prepared two other materials by increasing the amount of the Pc starting materials to 2 times (2-Fe5/carbon fibres) and 4 times (4-Fe5/carbon fibres) higher than the initial one to find the ideal amount of Fe5 nanostructures. They observed that it was possible to degrade RhB more efficiently with 2-Fe5/carbon fibres, followed by Fe5/carbon fibres and 4-Fe5/carbon fibres.

Still using visible light (*λ* > 400 nm), Das, Chattopadhyay, and co-workers^69^ studied the photooxidation of RhB in aqueous by using a copper(ii) phthalocyanine Cu4 (Fig. 4) functionalized with reduced graphene oxide (rGO) nanocomposite. Compared with Cu4, the degradation efficiency using the rGO/Cu4 nanocomposite was higher due to the redshift in the absorption spectrum that increases the catalytic activity under visible light (Table 10). The degradation efficiency reached 96% after 210 min of light irradiation when rGO was twice bigger than the Cu4 in the nanocomposite. The rGO played an essential role in enhancing the degradation efficiency due to the formed donor–acceptor system.

Li, Xu, and co-workers^70,136,139^ evaluated the degradation of RhB in aqueous media with visible light (*λ* > 400 nm) and composites of zinc(ii) phthalocyanine Zn4 (Fig. 4), Zn36, and Zn38 (Fig. 11) with MWCNTs. In the first study,^136^ there was almost no degradation rate (∼19%) using MWCNTs due to the high specific surface area of these CNTs. After immobilization with Zn36 and after 240 min, it was possible to degrade 88% of RhB with the hybrid Zn36–MWCNT. More importantly, after three cycles, the degradation efficiency for RhB did not suffer any significant change (just a slight decrease of 4%). Regarding the second study,^70^ of the pure Zn4, MWCNTs, and Zn4/MWCNT hybrids exhibited a photodegradation efficiency of 54%, 4%, and 93%, respectively, within 60 min of irradiation (Table 10). The hybrid material reveals a superior photocatalytic activity compared with pure Zn4 and MWCNTs due to the MWCNTs that prevented the Zn4 aggregation and increased the catalytic active sites. According to the authors, the photocatalytic rate did not significantly change (89%) after reusing the Zn4/MWCNT for three consecutive experiments. In the later study,^139^ after 120 min of irradiation, it was possible to achieve 93% of RhB degradation with hybrid Zn38–MWCNTs when compared with bare MWCNT (14%) and Zn38 (54%).

**Fig. 19:**
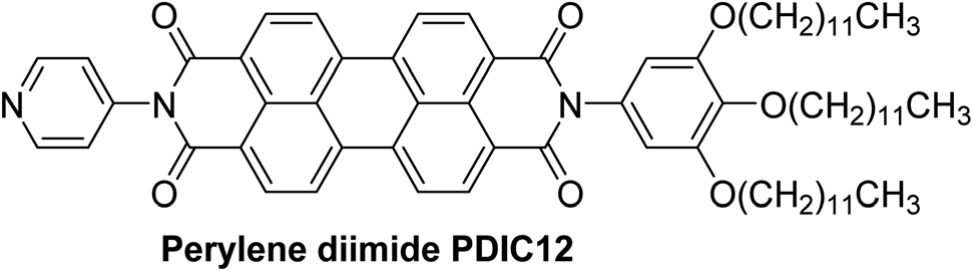
Perylene diimide derivative used by Zhang and co-workers^166^ for conjugation with Cu47 and used for the degradation of RhB.

Zhang and co-workers^166^ photodegraded RhB in aqueous media using Cu47/perylene diimide (PDIC12) (Fig. 19) p–n heterojunction under visible light irradiation (*λ* > 400 nm). This composite was based on the intramolecular hydrogen bond between the pyridyl moiety of the perylene and the carboxy group of Cu47. This type of composite shows an increase in photocatalytic activity compared with the precursors (PDIC12 and Cu47). Also, a higher molar fraction of the PDIC12 derivative on the composite allowed a higher photocatalytic degradation rate of RhB, reaching 80% after 180 min (Table 10).

Varghese and co-workers^71^ investigated the ability to degrade RhB under UV light irradiation in aqueous media. The authors prepared three different inorganic transition metal tungstate (NiWO_4_)/Sn(iv)4 (Fig. 4) nanocomposites based on the Sn4 content: C1 (1 wt%), C2 (2 wt%), and C3 (3 wt%). The best photocatalytic rate was obtained in the following order: C1 (71%) > C2 (49%) > C3 (48%) > Sn4 (37%) > NiWO_4_ (30%) – Table 10. The use of UV-visible light for the degradation of RhB in aqueous media was studied by Li, Shao, and co-workers.^167^ The authors use PAN nanofibers functionalized with Cu5 (Fig. 4 and bismuth oxychloride (BiOCl) nanosheets. The degradation rate of RhB using BiOCl/Cu5/PAN nanofibers is ∼6 times higher when compared with Cu5/PAN and BiOCl/PAN nanofibers. After 180 min, it was possible to degrade ∼80% of RhB. After three experiments, there was no decrease in the degradation rate of RhB (Table 10). On the other hand, using visible light irradiation (*λ* > 400 nm), Zhao, Han, and co-worker^73^ used as catalysts amidoximated PAN fibres anchored onto iron phthalocyanine Fe4 (Fig. 4)/TiO_2_ through coordination bonds. The best photocatalytic activity was achieved with an optimal amount of 10.2 wt%. After 80 min, it was possible to complete RhB degradation (Table 10). After 5 cycles, the photocatalytic activity rate remained intact.

**Fig. 20:**
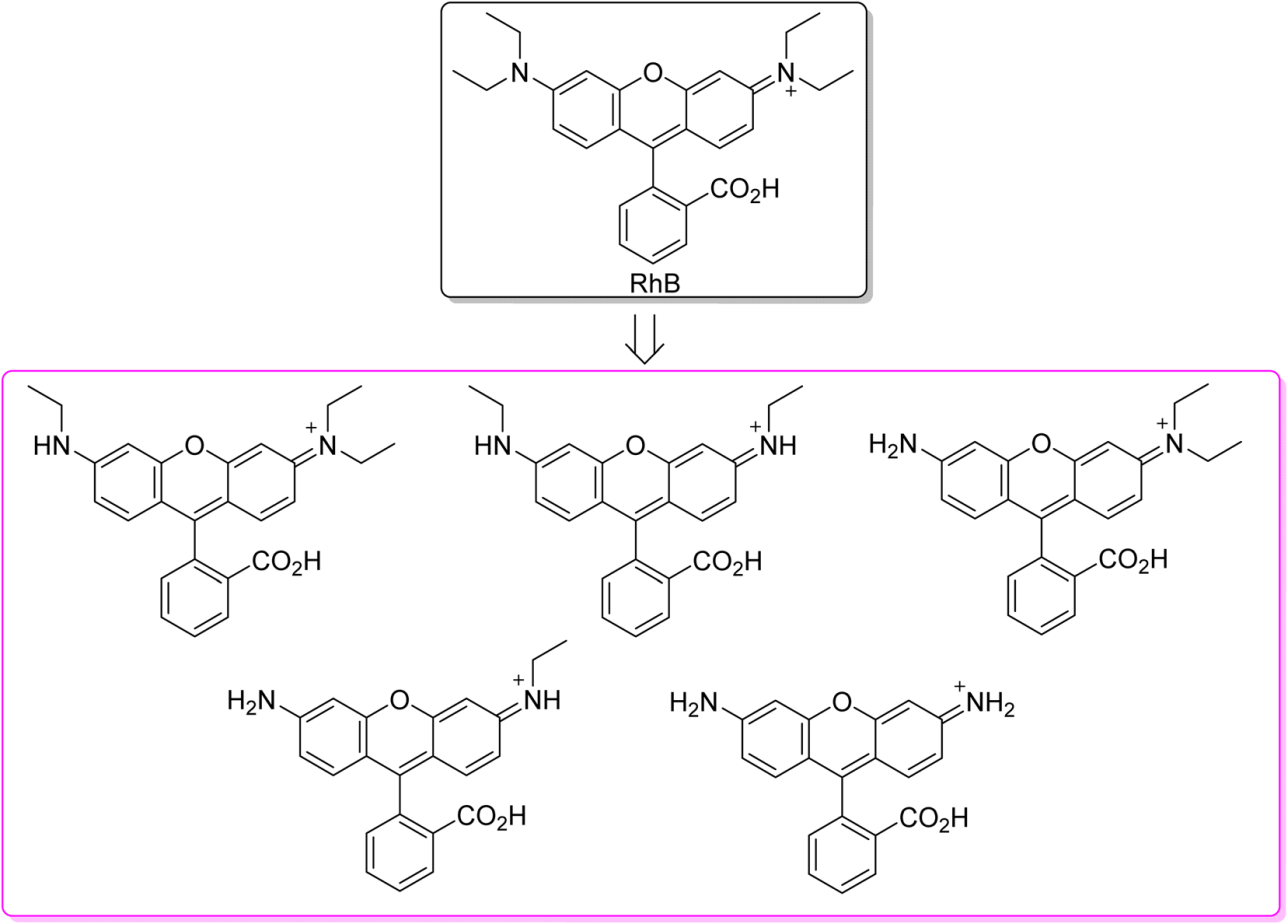
First intermediates in the degradation of RhB resulted from the cleavage of ethyl groups.

Still using PAN fibres and RhB as a pollutant under visible light and solar irradiation, Lu, Chen, and co-workers^56^ studied its degradation in aqueous media using a photocatalyst based on PAN-supported g-C_3_N_4_ coupled with Zn(ii) phthalocyanine 2 (Fig. 4) nanofibers. In the visible light studies, the removal rate of RhB using g-C_3_N_4_/Zn2/PAN as photocatalyst was higher than g-C_3_N_4_/PAN, reaching a maximum of ∼98% after 120 min of light irradiation (Table 10). Under solar irradiation, almost a complete degradation of RhB was achieved after 180 min with the g-C_3_N_4_/Zn2/PAN. The degradation rate could be maintained after three cycles of experiment with g-C_3_N_4_/Zn2/PAN. The first degradation products are presented in Fig. 20. After this, these intermediates are degraded into the ones mentioned by Han, Zhao, and co-workers^63^ (Fig. 17).

By also using solar irradiation, Oki and co-workers^74^ studied the photooxidation of RhB using graphene–ZnO composite with a Co4 (Fig. 4). There was a complete degradation using GR–ZnO and GR–ZnO–Co4 after 140 and 150 min under sunlight irradiation, respectively (Table 10). On the other hand, there was only a 20–60% degradation when using the Co4, ZnO, and GR alone as catalysts. Under the same experimental conditions, the degradation efficiency of GR–ZnO–Co4 was found to be higher than GR–ZnO catalyst. The interactions between the GR nanosheets and ZnO particles enhanced the photocatalytic activity of the GR–ZnO composite when compared with bare GR and ZnO. The GR nanosheets were the main ones responsible for the absorption of RhB and its posterior degradation. The sensitization with Co4 enhances the composite's absorption ability, increasing the photocatalytic efficiency. Still regarding the degradation of RhB and using the same phthalocyanine 8 but with N-PC (Cu4/N-PC), Dabiri and co-workers^144^ prepared a series of hybrids with different weight ratios: Cu4_0.125_/N-PC, Cu4_0.25_/N-PC, and Cu4_0.5_/N-PC. After 90 min of visible light irradiation (*λ* > 400 nm), there was no degradation for RhB with Cu4_0.125_/N-PC. However, it was possible to observe a more efficient photocatalytic degradation with Cu4_0.25_/N-PC (0.0627 min^−1^) than with Cu4_0.5_/N-PC (0.0551 min^−1^) in the presence of H_2_O_2_. Also, after performing seven experiments with the best photocatalyst, it was possible to maintain most of the activity (4% loss) –Table 10.

Huang and co-workers^168^ studied the photooxidation for RhB in aqueous media under visible light irradiation (*λ* > 420 nm) with a tetra-substituted manganese(ii) phthalocyanine bearing sulfonic acid groups Mn3 (Fig. 4) immobilized on a TiO_2_–SiO_2_ hybrid support. After 240 min of irradiation, complete degradation of RhB was achieved (Table 10). Under visible light irradiation (*λ* > 400 nm), Chen and co-workers^106^ studied the degradation of RhB using a magnetically recyclable composite AT/Fe_3_O_4_–Fe18 (Fig. 6). This composite was prepared using Fe18 and magnetic palygorskite nanoparticles (AT/Fe_3_O_4_). The remarkable superparamagnetic properties of this composite allow their recuperation by simply applying an external magnetic field. Different composites were prepared based on the amounts of phthalocyanine. The photodegradation rate was 96% after 300 min of light irradiation (Table 10) using Fe18 (0.6 nmol). However, the best photocatalytic activity was obtained with higher amounts of Fe2 (0.6 nmol). More recently, Zang, Sun, Zhang, and co-workers^93^ studied the degradation of RhB in an aqueous solution under visible light irradiation (*λ* > 420 nm) by using the Cu4 (Fig. 4) supported by PACA HIOBs. After 40 min of light irradiation, there was a complete degradation of RR3B with a degradation rate of 0.1218/min. After five cycles, the degradation efficiency was still 97.3%. The authors also assessed these photocatalysts' ability to degrade other anionic dyes, such as RBBR. Regarding RhB, the authors did not study the degradation products.

##### 3.2.3.2. Rhodamine 6G

As RhB, RhG is also a chemically stable dye that is difficult to degrade.^75^ Nyokong and co-workers^75^ evaluated the photocatalytic degradation of RhG in aqueous media using bare zinc(ii) tetraaminophthalocyanine Zn6 (Fig. 4) or conjugated with Ag nanoparticles. Both were incorporated into chitosan beads to facilitate the recovery after photocatalysis. In the presence of Ag nanoparticles, the photocatalytic degradation under visible light irradiation (*λ* > 400 nm) of RhG was enhanced (*t*_1/2_ = 41 min) when compared with Zn6 immobilized onto chitosan beads (*t*_1/2_ = 46 min, Table 10). Besides Ag nanoparticles, the previous authors^137^ also used the peripherally substituted phthalocyanine 35 (Fig. 11) but conjugated with ZnO affording Zn35–ZnO/PS fibres. The authors compared this composite's photocatalytic activity against RhB with Zn35–AgNPs/PS fibres. ZnO and AgNPs significantly improved the photocatalytic activity of the composites, being Zn35–AgNPs/Ps a better photocatalyst (shorter half-life times and higher degradation rate constant) compared to Zn35–ZnO/PS fibres (Table 10). The structure of the products was not identified, but the authors mentioned some peaks in the HPLC analysis that could be attributed to the removal of the diethyl group from the nitrogen atom, as presented in Fig. 20.

#### 3.2.4. Thiazine dyes

Thiazine dyes are a class of heterocycles with a low oxidation potential and a high propensity to form stable cations. When inadequately discharged, they can cause harmful impacts due to their high toxicity.^172^

##### 3.2.4.1. Methylene blue

Methylene blue (MB) has been shown to cause central nervous system toxicity. When released into wastewaters without treatment, it can cause a serious threat.^173^ So, Vallejo and co-workers^174^ studied the degradation of MB in aqueous media under visible light irradiation using Cu(ii)2 and Zn(ii)2 (Fig. 4) immobilized on TiO_2_. After 140 min, it was possible to achieve 47% and 30% of RhB degradation, respectively, which were 2.8 times and 3.6 times better than bare TiO_2_ (7%) (Table 11).

**Table 11:** Photophysical parameters of Pcs immobilized on several supports used for the photocatalytic activity of MB

MPc	Support	Light	Efficiency (%)	Time (min)	*k*_obs_ (min^−1^)	Recycle	Ref.
No	TiO_2_	Visible light (*λ* > 400 nm)	7	140	—	—	174
Zn2			30				
Cu2			47				
Co4		Solar	91	90			175
Fe4			97				
No			81				
Zn4		Visible (*λ* > 400 nm)	85 (3.7 nm)				76
			90 (3.9 nm)				
			70 (3.6 nm)				
			67 (3.8 nm)				
			42 (P25)				
Cu49			100				169
Zn52				100		5 cycles (∼16% loss)	38
Co52							
Ni52							
Cu52							
Cu18	g-C_3_N_4_		98	270		—	104
Cu4	ZnO			120			77
		UV (*λ* = 365 nm)		150			
Zn53	UIO-66 (NH_2_)	Visible (*λ* > 400 nm)	90	120		4 cycles (∼20% loss)	176
Zn49	P_2_W_17_		100	450		—	170
	P_2_W_18_		20				
	PW_12_		100	240			
Zn36	S-g-C_3_N_4_		97	100			78
Zn4			39				
Zn53			82				
Zn36	g-C_3_N_4_		90	120			138
	S-g-C_3_N_4_		82			3 cycles (∼30% loss)	
Co3	Fe_3_O_4_@SiO_2_@TiO_2_		100	30		3 cycles (0% loss)	177
		UV-vis		60		—	
Zn37	SiO_2_–TiO_2_	Visible (*λ* > 400 nm)		300			37
Cu4	PS-*b*-PAA + TiO_2_		—	—	2.9 × 10^−3^ (PS_241_-*b*-PAA_51_)		79
					1.8 × 10^−3^ (PS_330_-*b*-PAA_34_)		
					1.6 × 10^−3^ (PS_834_-*b*-PAA_162_)		
Cu50	silicate–TiO_2_		40 (10%^a^)	180	—		171
			33 (25%^a^)				
			30 (10%^a^)				
Zn50			35 (50%^a^)				
			28 (25%^a^)				
			24 (10%^a^)				
No			29 (50%^a^)				
			25 (25%^a^)				
Zn54	TiO_2_	Visible (*λ* > 420 nm)	100	110		3 cycles (18% loss)	178
Co54							
Cu54				130			
Ti55(O)							

a Amount of silicate polymer network.

Using TiO_2_ and Pc 4, Kwak, Chung, and co-workers^76^ and Varghese and co-workers^34^ both studied the degradation of MB in aqueous media under visible light irradiation with TiO_2_/Zn4 hybrids with several pore sizes and or sensitized TiO_2_ with M4 (M = Co(ii) or Fe(ii)) to form M4/TiO_2_ composites. In the first study,^76^ as expected, no MB degradation was observed within 90 min in the presence of all hybrids and the absence of light. The hybrids incorporating Zn4 showed higher photocatalytic activity when compared with the unmodified samples. The photocatalytic degradation efficiencies against MB were 85%, 90%, 70%, 67%, and 42% for L121–TiO_2_/Zn4 (3.7 nm of pore size), P123–TiO_2_/Zn4 (3.9 nm of pore size), F68–TiO_2_/Zn4 (3.6 nm of pore size), F127–TiO_2_/Zn4 (3.8 nm of pore size), and Degussa P-25/Zn4, respectively (Table 11). Herein, it was shown that the size of the pores did not influence the photocatalytic activity. The high photocatalytic activity is due to a cascade of Mie light scattering. The pore size of P123–TiO_2_/Zn4, which is comparable with the wavelength of the irradiation source, strongly generated the Mie scattering, followed by a considerable enhancement of the photocatalytic activity. In the second study, it was possible to achieve higher photocatalytic rates using Fe4/TiO_2_ (97%) when compared with Co4/TiO_2_ (91%) and bare TiO_2_ (81%) after 90 min under solar irradiation (Table 11). Despite having promising photocatalytic activities, it would be essential to evaluate the stability of the compounds within several cycles in future work.

For the degradation of MB in aqueous media, Zahmakiran, Agirtas, and co-workers^169^ also studied their photooxidation using a copper(ii) phthalocyanine Cu48 (Fig. 16) immobilized in TiO_2_. MB was degraded entirely within 90 min under visible light irradiation (Table 11). After five cycles, the photocatalyst could retain > 80% of its initial activity.

**Fig. 21:**
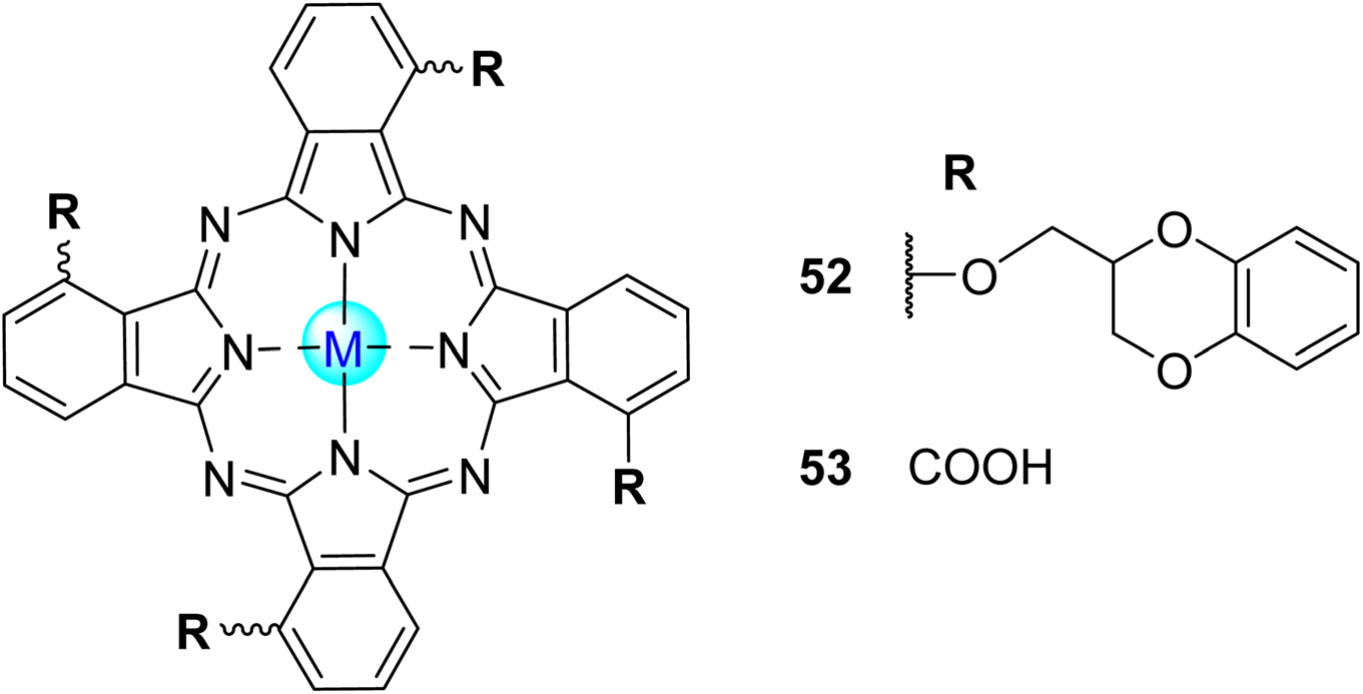
Tetra-α-substituted phthalocyanines 52^38,78^ and 53^176^ developed by Gorduk and Avciata,^38^ Li and Liang^78,176^ and co-workers.

The degradation of MB in aqueous media under visible light irradiation (*λ* > 400 nm) was performed using free-base and metallophthalocyanine M52 (M = Zn(ii), Co(ii), Ni(ii), or Cu(ii), Fig. 21) immobilized on TiO_2_ by Gorduk, Avciata, and co-workers.^38^ According to the authors, within 100 min, it was possible to achieve complete degradation of MB using all the composites. After being reused up to five times, they could maintain their activity above 76%.

Schneider and co-workers^104^ could degrade 98% of MB in aqueous media within 270 min under visible light irradiation using Cu18 (Fig. 6) immobilized in g-C_3_N_4_ (Table 11). Under UV (*λ* = 365 nm) or visible light (*λ* = 400–700 nm) irradiation, Maya-Treviño and co-workers^77^ studied the photodegradation for MB using Cu4 (Fig. 4) sensitized on ZnO through a sol–gel method. The authors prepared two materials varying the percentage containing Cu4. The first material contains 0.1% wt, and the second comprises 0.5% wt. According to the authors, after 120 min under visible light irradiation, it was possible to achieve 98% degradation for MB (Table 11). Compared with TiO_2_ (a widely known photocatalyst), it could only achieve similar percentages after 180 min of light irradiation. Under UV light irradiation, the same activity could be achieved after 150 min under light exposure. Li, Liang, and co-workers^176^ achieved a 90% degradation for MB in aqueous media after 120 min under visible light irradiation (*λ* > 420 nm) using a zirconium-based MOF, UIO-66 (NH_2_), covalently linked to Zn53 (Fig. 21). This photocatalytic rate was much higher when compared with a mixture of Zn53 and UIO-66(NH_2_) (Table 11). Also, after four cycles, the photocatalytic activity decreased to 20%.

**Fig. 22:**
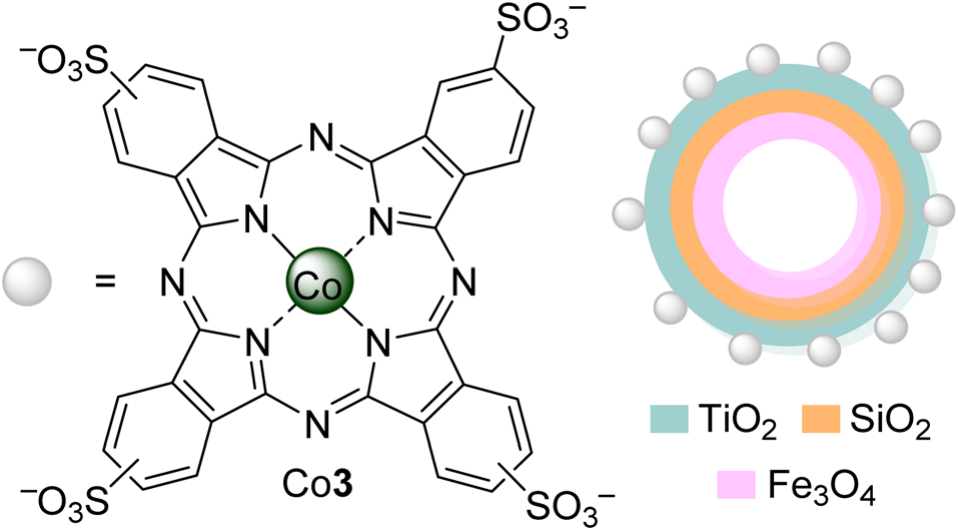
Hollow Fe_3_O_4_@SiO_2_@TiO_2_ nanostructure sensitized with Co3 used by Liu and co-workers^177^ for the degradation of MB.

Wang, Liu, and co-workers^170^ studied the degradation of MB in aqueous media using Zn49 (Fig. 16) immobilized in three different polyoxometalates (POMs): P_2_W_17_, P_2_W_18_, and PW_12_. Under visible light irradiation (*λ* > 400 nm), achieving complete degradation after 240 min and 450 min was possible using Z49/PW_12_ and Zn49/P_2_W_17_, respectively. However, after 450 min, only 20% of MB degradation was achieved using Zn49/P_2_W_18_. A degradation rate of 100% was achieved with Zn49/P_2_W_17_ with a small photocatalyst dosage (Table 11). Still using visible light irradiation (*λ* > 400 nm), Liang, Li, and co-workers^78,138^ studied the photooxidation of MB in aqueous media with sulfur-doped g-C_3_N_4_ (CNS) coupled with zinc(ii) phthalocyanines Zn36 (Fig. 11), Zn4 (Fig. 4), and Zn52 (Fig. 21). In the first study,^78^ composites exhibited higher photocatalytic degradation (39–97%) than bare CNS (30%). Zn36/CNS degrade ∼97% of MB after 100 min under irradiation. On the other hand, with Zn52/CNS and Zn4/CNS, the photocatalytic rate decreased to 82% and 39%, respectively (Table 11). In the second study,^138^ the pure g-C_3_N_4_ and Zn36 showed degradation efficiencies of 36% and 42%, respectively, within 120 min of irradiation. Incorporating sulfur in the g-C_3_N_4_ enhanced the photocatalytic activity of the catalysts. The activities of the composites followed the order: g-C_3_N_4_ < Zn36 < g-CNS < Zn36/g-C_3_N_4_ < Zn36/g-CNS. After three cycles, there is a decrease of 30% in the photocatalytic efficiency of the Zn36/g-CNS. Liu and co-workers^177^ studied the degradation of MB in aqueous media under visible light (*λ* > 400 nm) with cobalt(ii) phthalocyanine Co3–sensitized hollow Fe_3_O_4_@SiO_2_@TiO_2_ hierarchical nanostructures (Fig. 22). According to the authors, the composite showed better photocatalytic activity, reaching ∼100% degradation of MB after 30 min of irradiation compared with the UV-visible light (60 min). The photocatalyst could be separated by an external magnetic field and reused for three cycles without significant activity loss.

**Fig. 23:**
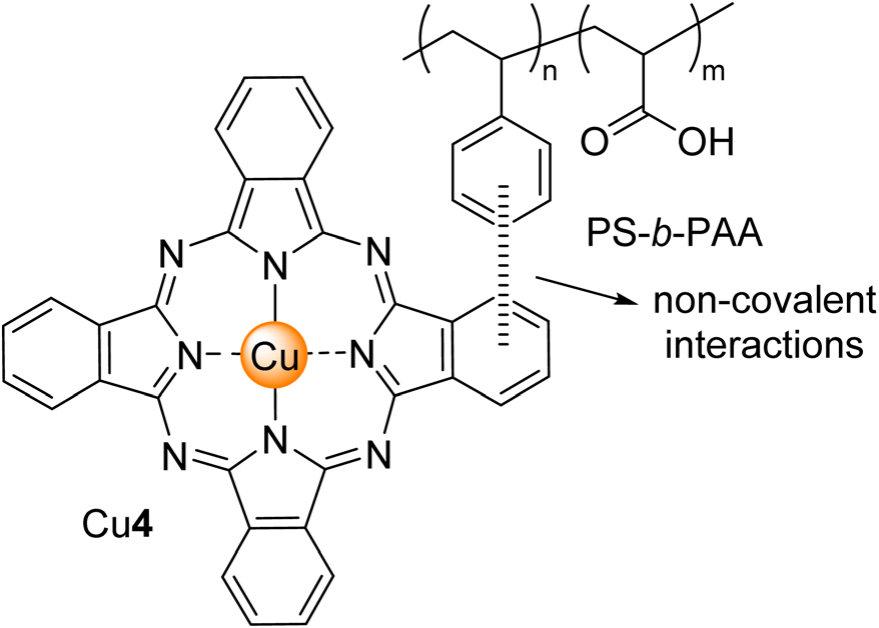
Cu4 and PS-*b*-PAA were used to incorporate TiO_2_ gel developed by Nakatani and co-workers.^79^

The degradation of MB in aqueous media under visible light irradiation (*λ* > 400 nm) using Zn37 (Fig. 11) bearing cyanuric chloride immobilized on SiO_2_–TiO_2_ microparticles (Zn37–SiO_2_–TiO_2_) through covalent bonds was studied by Yao and co-workers.^37^ After 300 min, it was possible to complete MB degradation in a saturated-O_2_ environment using the Zn37–SiO_2_–TiO_2_ hybrid. Despite the remarkable activity, there are no studies regarding the stability of the material for 300 min under light irradiation. Under the same light and media conditions, Nakatani and co-workers^79^ studied the degradation of MB with Cu4 incorporated in a poly(styrene-*block*-acrylic acid) (PS-*b*-PAA, Fig. 23) containing TiO_2_ gel. The authors prepared different lengths in the copolymers that afforded different photocatalytic activities. The photocatalytic activity under visible light irradiation was greatly improved (three times higher) when loading Cu4 into the system. It was possible to achieve better degradation rates with Cu4-PS_241_-*b*-PAA_51_ containing TiO_2_ gel (*k*_obs_ = 2.9 × 10^−3^ min^−1^) followed by Cu4-PS_330_-*b*-PAA_34_ (*k*_obs_ = 1.8 × 10^−3^ min^−1^) containing TiO_2_ gel and Cu4-PS_834_-*b*-PAA_162_ (*k*_obs_ = 1.6 × 10^−3^ min^−1^) containing TiO_2_ gel.

Saka and co-workers^171^ accessed the photodegradation of MB under visible light irradiation (*λ* > 400 nm) in aqueous media using hybrid organic–inorganic nanocomposite containing Cu(ii) and Zn(ii) phthalocyanines bearing 1,2,4-triazole groups modified TiO_2_ nanoparticles (Zn50–TiO_2_ and Cu50–TiO_2_). The hybrid polymer network used tetraethyl orthosilicate, 3-(glycidyloxypropyl)triethoxysilane. These Zn50 and Cu50 (Fig. 16) modified nanoparticles were added to the hybrid polymer network at 10% (M50–T_10_), 25% (M50–T_25_), and 50% (M50–T_50_). These coating solutions were then put in glass substrates through a spray methodology. After 180 min of irradiation, the best photocatalytic degradation was more efficient with Cu50–T_10_ with a 40% degradation rate, followed by Zn50–T_50_ (35%), Cu50–T_25_ (33%), Cu50–T_10_ (30%), bare T_50_(29%), Zn50–T_25_ (28%), bare T_25_ (25%), Zn50–T_10_ (24%), and bare T_10_ (20%).

**Fig. 24:**
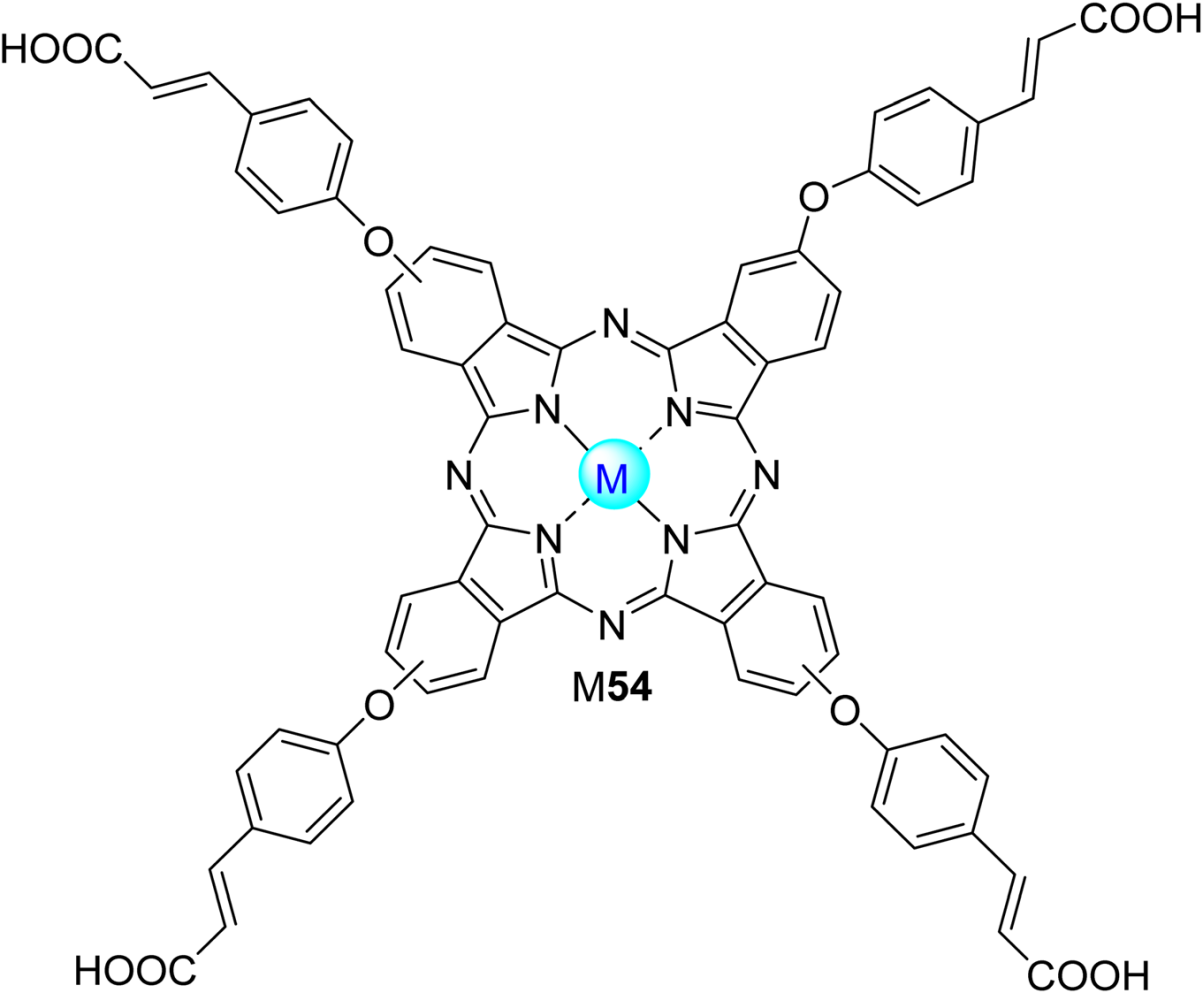
Tetra-β-substituted phthalocyanine 54 used for the photodegradation of MB by Karaoğlan and co-workers.^178^

Karaoğlan and co-workers^178^ could completely degrade MB using M54 (M = Zn(ii), Co(ii), Cu(ii), and TiO(iv)(OPr)) (Fig. 24) and visible light irradiation (*λ* > 420 nm) in aqueous media. After 110 min, it was possible to completely degrade MB using Zn54 and Co54, whereas it took Cu54 and Ti54O 130 min to achieve the same degradation. After 3 reusability cycles, there was only a loss of 18% for all the photocatalysts. The authors did not study the degradation products of MB.

#### 3.2.5. Xanthene dye – fluorescein

Xanthene dyes are characterized by their intense fluorescence, being fluorescein the most well-known example. This dye is the most common marker for maritime accidents or tracers for underground rivers. For this reason, it is commonly found in wastewater.^179^ Finding an environmentally friendly process to degrade this pollutant is a very attractive task. So, for the photodegradation of fluorescein (FC) in aqueous media under visible light, Raducan and co-workers^86^ used Cu3–TiO_2_, Cu4–TiO_2_ (Fig. 4), and Cu25–TiO_2_ (Fig. 6). The different photocatalytic activities of these nanocomposites in aqueous media revealed that the best photocatalyst was Cu3–TiO_2_ (90%), followed by Cu25–TiO_2_ and Cu4–TiO_2_. However, when using bare TiO_2_, a 90% photodegradation rate was also achieved, meaning that no improvement was observed with the addition of the phthalocyanines. Regarding the recyclability studies, the authors selected Cu3–TiO_2_ and performed it in another dye: brilliant blue.

#### 3.2.6. Anthraquinone dye – remazol brilliant blue R

Anthraquinone dyes, more specifically, RBBR is a vinyl sulfone-based formazan dye known for its bright color, easy application techniques, low energy consumption in the dyeing process and high-water solubility. Its discharge into the environment can seriously harm organisms in aquatic life due to their toxicity, carcinogenicity, and non-biodegradability.^180^ So, Zang, Sun, Zhang and co-workers studied^93^ the degradation of RBBR in an aqueous solution under visible light irradiation (*λ* > 420 nm) by using the Cu4 (Fig. 4) supported by PACA HIOB. After 40 min of light irradiation, there was a complete degradation of RBBR with a degradation rate of 0.0461/min. Regarding RBBR, the authors did not study the degradation products or recyclability.

### 3.3. Agrochemicals

**Fig. 25:**
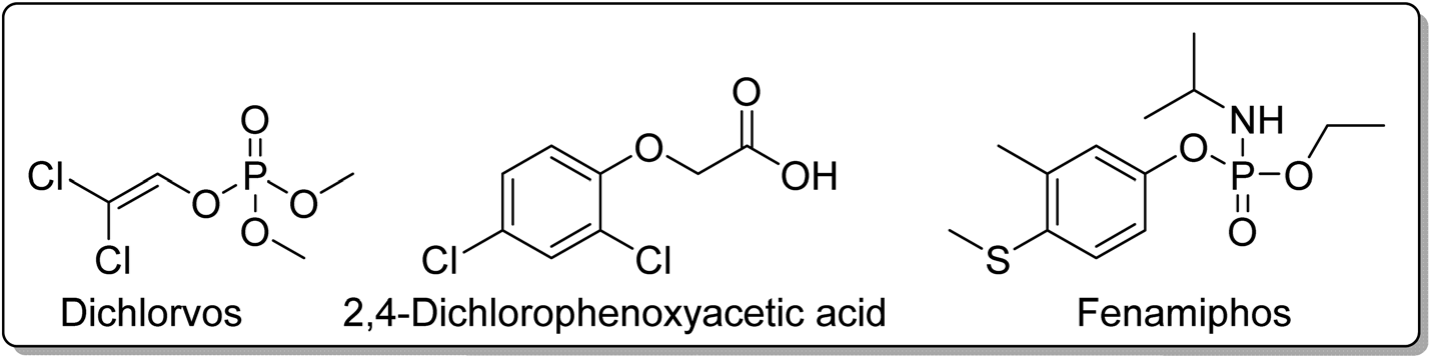
Structure of the agrochemicals mentioned in this review.

Agrochemical and pharmaceutical compounds play very important roles in modern society, providing greater quantity and quality of food as well as health and well-being for the entire population. However, due to the worldwide massive amounts of used agrochemicals (mainly insecticides, fungicides, and herbicides), their negative impact on the environment is unquestionable. When these compounds reach humans, they can cause neurotoxic, endocrine-disruptor, and DNA-damaging effects.^181^ The photodegradation of dichlorvos, 2,4-dichlorophenoxyacetic acid, and fenamiphos (Fig. 25), catalysed by phthalocyanines, is discussed in the following sections.

#### 3.3.1. Dichlorvos

Dichlorvos (Fig. 25) is an organophosphate used as an insecticide to control household pests. It is soluble in water and difficult to degrade or absorb sediment. Its toxicity appears as an irreversible inhibitor of acetylcholinesterase. This inhibition causes an accumulation of acetylcholine in synapsis, providing a disruption of nerve function.^182^ So, its degradation becomes crucial when found in wastewater. For this reason, Núñez and co-workers^183^ studied the photodegradation of dichlorvos in aqueous media (pH = 7) with Cu3 (Fig. 4) adsorbed on TiO_2_-Degussa P25 and UV and/or visible light irradiation. Under visible light, the best photocatalytic activity was obtained when using Cu3–TiO_2_ (*k*_obs_ = 0.7 × 10^2^ min^−1^) with a lower surface coverage area (*θ* = 9%). Under UV light, anatase TiO_2_ could achieve a high rate of dichlorvos degradation: *k*_obs_ = 1.0 × 10^2^ min^−1^. Under simulated solar light (visible + UV), the photocatalytic rate constant increases for the best photocatalyst: Cu1–TiO_2_ (*k*_obs_ = 1.2 × 10^2^ min^−1^) with the higher surface coverage area (*θ* = 79%). High Cu3–TiO_2_ surface coverage decreases the O_2_ oxidation (sensitization *via* visible light) but increases the water's approach to the surface due to a less distribution of Cu3 on the photocatalyst surface. In the low surface coverage area (*θ* = 9%), the UV irradiation was less effective due to the parallel distribution of Cu3, which limited the proximity of water and the formation of hydroxyl radicals.

#### 3.3.2. 2,4-Dichlorophenoxyacetic acid

**Fig. 26:**
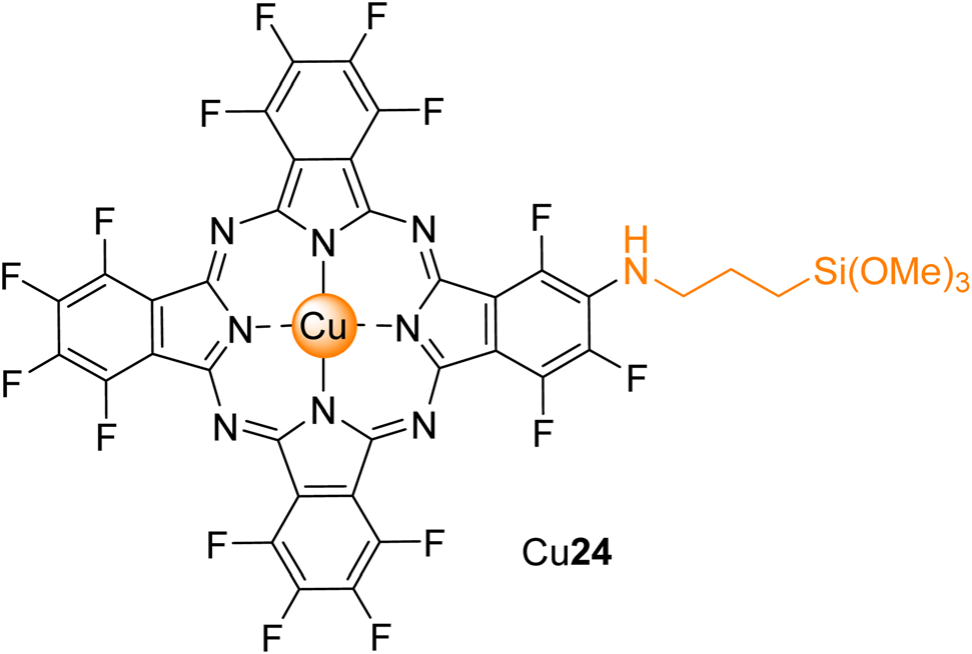
Cu24 functionalized with 3-aminopropyltrimethoxysilane developed by Serra and co-workers.^108^

2,4-Dichlorphenoxyacetic acid is a widely used herbicide. It is soluble in water and distributes throughout the body without a specific accumulation. Also, studies are associated with its oncogenicity, genotoxicity, and neurotoxicity. Given all of this, its removal from wastewater is crucial.^184^ The photooxidation of 2,4-dichlorophenoxyacetic acid in aqueous media under UV light exposure using a mesoporous photocatalyst based on the Cu24 (Fig. 26) and 3-aminopropyltrimethoxysilane was studied by Serra and co-workers.^108^ After condensation of tetraethylorthosilicate around a micelle, the hexagonal mesoporous Si–Cu24 was obtained. According to the authors, achieving almost 90% of pesticide degradation after 30 min in the presence of H_2_O_2_ was possible. After 6 photocatalytic cycles, 30% of the photocatalytic efficiency was reduced and maintained constant until the tenth cycle. This reduction could be due to the significant amount of Cu24 leached after ten cycles. Once again, the Cu24 practically does not leach between the sixth and tenth cycles.

#### 3.3.3. Fenamiphos

**Scheme 1:**
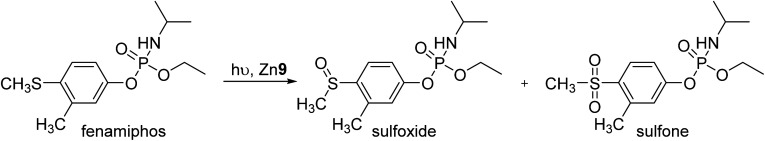
Fenamiphos photooxidation products.

Fenamiphos is a nematicide used to control a wide variety of pests. Its toxicity has been studied in aquatic and terrestrial organisms. Its removal by photocatalysis has already been studied.^185^ Pereira, Azenha and co-workers^85^ studied the photodegradation of fenamiphos in aqueous media with Zn5, Zn7, and Zn16 (Fig. 4) immobilized into Al-MCM-41. After 300 min under UV light irradiation (*λ* = 320–460 nm), the best photocatalytic rate was obtained with Zn5/Al-MCM-41 (*k*_obs_ = 8.5 × 10^−3^ min^−1^ for fenamiphos) and the cationic derivative Zn16 (*k*_obs_ = 8.1 × 10^−3^ min^−1^ for fenamiphos). The cationic material Zn16/Al-MCM-41 could maintain a stable photocatalytic activity after three reusability cycles. In contrast, when using Zn5/Al-MCM-41, there was a decrease of 40% in the photodegradation rate after the same cycles. The authors identified sulfone and sulfoxide (Scheme 1) as the degradation products.

### 3.4. Pharmaceuticals

**Fig. 27:**
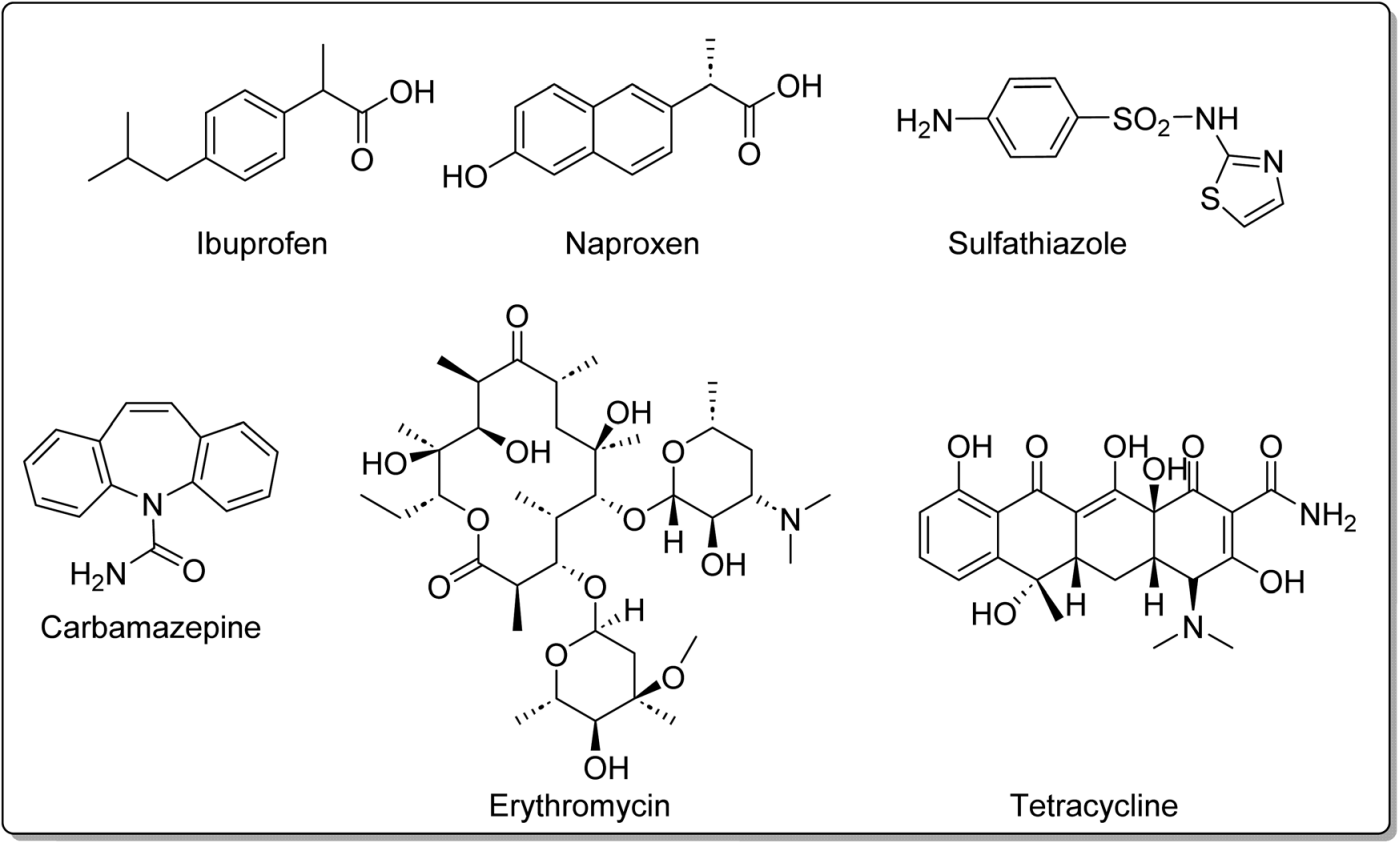
Pharmaceuticals for photodegradation studies mentioned in this review.

Antibiotics, anti-inflammatory, and anti-depressive drugs are among the thousands of human pharmaceuticals produced and consumed yearly. These drugs reach aquatic habitats *via* the disposal of domestic sewage. When they reach the wastewater treatment plants, after some degradation process, they can be converted into other potentially more harmful and long-lasting chemicals.^186^ Thus, it seems evident that finding innovative and sustainable treatment processes to remove these pollutants from wastewater is essential. In the following sections, the degradation of ibuprofen, naproxen, sulfathiazole, carbamazepine, erythromycin, and tetracycline (Fig. 27) using several phthalocyanines will be mentioned.

#### 3.4.1. Ibuprofen and naproxen

Ibuprofen and naproxen are anti-inflammatory drugs widely described. The main source of contamination of these drugs is the excretion of non-metabolized and metabolized drugs in human urine. To avoid the harmful health consequences of ibuprofen and naproxen and its metabolites, removing them from natural water and wastewater is necessary.^187,188^ To photodegrade ibuprofen and naproxen in aqueous media, Mlynarczyk and co-workers^189^ used a material based on Zn4 or Cu4 (Fig. 4) immobilized in TiO_2_. Different photocatalytic activities were obtained depending on the catalyst and type of light used. Both photocatalysts can degrade naproxen more efficiently than ibuprofen. For all the experimental conditions, introducing Zn4 or Cu4 could enhance the photocatalytic activity of bare TiO_2_. When UV irradiation was used, complete degradation of naproxen with Zn4–TiO_2_ and Cu4–TiO_2_ within 120 min under irradiation. In the case of ibuprofen, more time was needed (360 min) to achieve a 90% degradation using Cu4–TiO_2_ and 85% with Zn4–TiO_2_. Under red light irradiation, neither catalyst could degrade ibuprofen. After 3 cycles, it was still possible to completely degrade naproxen with Zn4–TiO_2_ within 180 min (instead of 120 min in the first cycle).

#### 3.4.2. Erythromycin

Erythromycin is the first antibiotic used to treat human infections. Despite most erythromycin and other antibiotics being discharged into the sewage system, reaching wastewater treatment plants, the inadequate disposal of unused medicines also reaches these plants. Their removal from wastewater is essential.^190^ So, Suganthi, Vignesh, and co-workers^94^ studied the degradation of erythromycin in aqueous media under visible light (*λ* > 400 nm) using Zn4 (Fig. 4) with modified TiO_2_ nanoparticles (Zn4–TiO_2_). The composite was prepared by chemical impregnation and used to improve the photocatalytic activity of TiO_2_. According to the authors, the composite has a slightly shifted absorption to the visible region of the spectrum and a higher surface area when compared with TiO_2_. As expected, after 180 min under light irradiation, it was possible to degrade erythromycin using Zn4–TiO_2_ (74%) and bare TiO_2_ (32%). The photodegradation rate could be maintained after 5 cycles with the same catalyst.

#### 3.4.3. Tetracycline

Tetracycline is one of the most effective broad-spectrum antibiotics. It gets into the environment through urine and faeces since it is completely absorbed but has a low metabolic transformation. Due to its high stability, it becomes difficult to degrade it under natural conditions. So, it becomes important to find new, effective, and feasible technologies for the degradation of tetracycline in wastewater.^191^ Lu and co-workers^80^ could photodegrade tetracycline in aqueous media in the presence of Cu5 (Fig. 4)/CeO_2_/Bi_2_MoO_6_ nanofibers and sunlight. After 120 min, it was possible to degrade ∼95% of the tetracycline compared with the material by itself (50%) (without the Cu5). This enhanced photocatalytic activity can be correlated to the synergetic effect between Cu5, CeO_2_, and Bi2MoO_6_. More recently, Li, Guo, and co-workers^81^ developed a Fe4/g-C_3_N_4_ heterojunction nanosheets for the photodegradation of TC. After 40 min of visible light irradiation (*λ* > 420 nm), 97% of TC was degraded. According to the authors, the construction of this heterojunction *via* π–π conjugation inhibited phthalocyanine aggregation, promoting charge separation and transfer and broadened the response range of visible light. The degradation of TC was extent to wastewater and the degradation rate was maintained. However, the authors did not perform recyclability studies or identify the degradation products of TC.

More recently, Yang, Yu, and co-workers^82^ studied the degradation of TC in an aqueous solution under visible light irradiation (*λ* > 420 nm) by using the Fe4 (Fig. 4)/perylene diimide heterojunctions. After 60 min of light irradiation, there was degradation of 79% of TC (degradation rate of 0.0264/min). This degradation rate was higher when compared with perylene diimide (41%, 0.0088/min) and Fe4 (2%, 0.0003/min) by itself. After five cycles, the degradation efficiency was almost unchanged. The authors did not study the degradation products of TC.

#### 3.4.4. Carbamazepine

Carbamazepine is an important drug used to treat epilepsy and other psychotherapy applications. This drug is one of the most frequently detected pharmaceuticals in wastewater and their corresponding metabolites.^192^ So, Lu, Chen, and co-workers^56,109^ studied the photooxidation in aqueous media of carbamazepine using PAN-supported g-C_3_N_4_ coupled with Zn(ii) phthalocyanine 2 (Fig. 4) nanofibers and an iron hexadecachlorophthalocyanine Fe25 (Fig. 6) coordinated with g-C_3_N_4_ previously functionalized with pyridine-based ligand isocotinic acid (INA). According to the first study,^56^ the g-C_3_N_4_/Zn2 was introduced as the catalytic entity, and the PAN nanofibers were employed as support to overcome the defects of easy aggregation and enable an easy recycling process. Under solar irradiation, the g-C_3_N_4_/Zn2/PAN could only induce degradation of ∼98% after 300 min, which could be maintained five times during the experiment. In the second study,^109^ nearly 55% of carbamazepine was degraded over g-C_3_N_4_ or g-C_3_N_4_/Fe25 in the presence of peroxymonosulfate within 40 min under visible light irradiation. In the presence of g-C_3_N_4_–INA–Fe25, the removal rate was much higher (∼94%). Almost no degradation was observed without light, even in the presence of peroxymonosulfate. Regarding the degradation products, the authors reported the identification of several intermediates by mass spectrometry in both studies. The degradation products are very similar between the two studies. The difference between them is the utilization of peroxymonosulfate in the second study.^56,109^

**Fig. 28:**
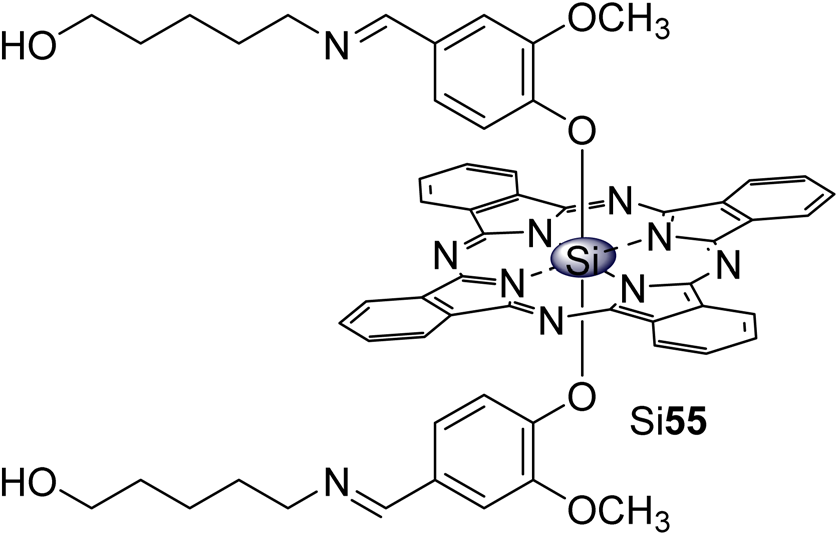
Silicon phthalocyanine Si55 used by Anucha, Altin and co-workers^193^ for the degradation of carbamazepine.

In another study, Anucha, Altin, and co-workers^193^ studied the photooxidation of carbamazepine in aqueous media under UV-irradiation using boron/sodium fluorine co-doped titanium dioxide sensitized with an axial substituted phthalocyanine Si55 (Fig. 28) (B/NaF–SiHTiO_2_). After 240 min, there was complete degradation using the B/NaF–Si55TiO_2_, whereas the unsensitized B/Na–TiO_2_ could only achieve 70% of carbamazepine degradation. Both composites showed higher photocatalytic activity when compared with the bare TiO_2_ (40%). In this study, the authors identified similar degradation products to the ones reported by Lu, Chen, and co-workers.^56,109^

#### 3.4.5. Sulfathiazole

The presence of medicines, their metabolites and/or their degradation products, is a current problem.^194^ The photodegradation of sulfathiazole in aqueous media under visible light irradiation (*λ* > 400 nm) was studied by Yang and co-workers.^195^ They used well-faceted TiO_2_ nanosheets with coexposed (001) and (101) and deposited selectively on α-Fe_2_O_3_ and Fe/Co–N_4_ on (001) facets (NTiO_2_–NS). Then, they prepared a hybrid material containing NTiO_2_–NS and Co3 (Fig. 4). There was 65% sulfathiazole degradation with the prepared catalyst, which was much higher when compared to NTiO_2_–NS (20%). Also, even after 40 recycle usages, the photocatalytic performance had no significant changes.

### 3.5. Other pollutants

Other pollutants such as trichlorobenzene (TCB), aniline, and dimethyl phthalate were found in wastewater. These aromatic compounds are considered toxic and should be eliminated from wastewater.^83,99,196^ In the following three studies, the authors analysed the degradation of these pollutants by using two different phthalocyanines.

#### 3.5.1. Chlorobenzene and 1,2,4-trichlorobenzene

Gül and co-workers^99^ evaluated the photocatalytic degradation of chlorobenzene (PhCl) and TCB using TiO_2_ sensitized with Co15 and Zn15 (Fig. 4) under visible light (*λ* > 400 nm). Compared with the previous study, the Co15 and Zn15 could also remove PhCl and TCB within 30 min under visible light irradiation. The recyclability studies showed no more than 16% loss after 5 cycles (Table 12).

**Table 12:** Efficiency *vs.* recyclability of Co15/Zn15–TiO_2_ composites for the photocatalytic degradation of 4-CP, PhCl, and TCB

Catalyst	Pollutant	Photocatalyst activity (% of degradation)				
		1st cycle	2nd cycle	3rd cycle	4th cycle	5th cycle
Co15–TiO_2_	4-CP	99	97	95	92	91
Zn15–TiO_2_		98	95	93	90	88
Co15–TiO_2_	PhCl	99	96	94	91	90
Zn15–TiO_2_		97	94	90	87	86
Co15–TiO_2_	TCB	98	93	92	88	87
Zn15–TiO_2_		95	91	89	86	84

#### 3.5.2. Aniline

The photodegradation of aniline in aqueous media under visible light using chitosan–H_2_4–TiO_2_ (Fig. 4) hybrid was accessed by Bouattour and co-workers.^196^ In the presence of unmodified samples, there was a 30% degradation of aniline within 600 min. After sensitization with H_2_4, two different hybrids were obtained according to the amount of phthalocyanine: A–H_2_4/chitosan–TiO_2_ (1 wt%) and B–H_2_4/chitosan–TiO_2_ (2 wt%). Under visible light irradiation, the A–H_2_4/chitosan–TiO_2_ and B–H_2_4/chitosan–TiO_2_ have higher photocatalytic activity when compared with the unsensitized hybrid and bare TiO_2_. After 10 h, it was possible to degrade ∼70% and 50% of aniline using A–H_2_4/chitosan–TiO_2_ and B–H_2_4/chitosan–TiO_2_, respectively. The second catalyst could be reused up to three times, with a loss of 20% on their photocatalytic activity. This loss was mainly observed from the first to the second cycle (16%).

#### 3.5.3. Dimethyl phthalate

Chang and co-workers^83^ studied the photooxidation of plasticizer dimethyl phthalate in aqueous media using magnetic hybrid photocatalysts from Cu4 (Fig. 4)/TiO_2_/silica/magnetite. The best photocatalytic rate was achieved with 1.92 wt% (0.0009 min^−1^), only half of P25, a well-known photocatalyst (0.001715 min^−1^). However, after 10 cycles, no significant loss of activity was observed (20% rate decrease).

## 4. Summary and outlook

This review shows the significant advances for the photocatalytic degradation of several water pollutants using simple Pc derivatives or Pcs immobilized on organic or inorganic materials, such as polymers, fibres, carbon nanostructures, ZnO, SiO_2_, TiO_2_, or mixtures of these supports, when exposed to UV, visible, UV-visible, and/or solar light irradiation. It is also shown that the photocatalytic activities of the phthalocyanine dyes are strongly correlated with their substituents, the presence/absence of positive charges, and the metal ions on their macrocycle structure.

The light source (UV, UV-visible, visible, or solar) is also a key element for the photodegradation approach. When solar/visible light is used, the Pc can be excited along with the support, but only a small or no excitation of the Pcs is observed using UV light. The use of solar light is considered an enormous advantage for photocatalysis since it reduces costs and takes advantage of the solar spectrum. It is also important to mention that most photocatalytic studies lack reusability studies, an essential parameter for the potential industry application. However, for most of the reused catalysts, no significant loss of activity was observed, and, in some cases, the photocatalyst is specific for a selected pollutant.

This review highlights the efforts of the scientific community to find new alternative methodologies for the degradation of pollutants, which are usually difficult to remove from water. The most critical issue is the prevention of Pcs leaching, which was revealed to be the leading cause of activity loss of the photocatalysts after several cycles of their use. Almost all the works mentioned in this review referred to the recyclability of the photocatalysts. Fortunately, the tendency to carry out those studies has been increasing over time. Moreover, for a scientist who wants to reproduce the reported work, it is important to know the exact conditions of the photocatalytic assay, namely the irradiance of the lamp used. However, most of the cited papers in this review did not mention the irradiance of the lamp used (nor the lamp's brand), making it difficult to compare results and even reproduce them. Nevertheless, the application of these photocatalysts in wastewater is (urgently) needed to offer an important insight for addressing future environmental challenges.

## Literature review information

The search for research papers for this review was conducted in 2021, but it was updated in April 2023 before the submission of the manuscript. This review has 103 papers on photocatalytic studies and a total of 196 references dated from 2007 to 2023.

## Abbreviations

2,4-DCP: 2,4-Dichlorophenol
2,4,6-TCP: 2,4,6-Trichlorophenol
4-CP: 4-Chlorophenol
4-NP: 4-Nitrophenol
AO7: Acid orange 7
BB: Brilliant blue
BPA: Bisphenol A
BPB: Bromophenol blue
BR29: Basic Red 29
BR195: Basic Red 195
CMP: Conjugated microporous polymers
CV: Crystal violet
DCM: Dichloromethane
FS: Fuchsine
FSC: Fluorescein
GR: Graphene
MB: Methylene blue
MNP: Magnetic nanoparticle
MO: Methyl orange
MPc: Metallophthalocyanine
MR: Methyl red
MWCNT: Multi-walled carbon nanotubes
N-GR: Nitrogen-doped graphene
NPS: Nanoparticles
OG: Orange G
PA-6: Polyamide-6
PAA: Polyacrylic acid
PAN: Polyacrylonitrile
PCP: Pentachlorophenol
Pc: Phthalocyanine
PDIC12: Perylene diimide C_12_ derivative
P4R: Ponceau 4R
PS: Polystyrene
PSU: Polysulfone
PUR: Polyurethane
RDB: Reactive dark blue
rGO: Reduced graphene oxide
RBBR: Remazol brilliant blue R
RhB: Rhodamine B
RhG: Rhodamine G
ROS: Reactive oxygen species
SB: Select brown
SFB: Sella fast black
SWCNT: Single-walled carbon nanotubes
TCB: 1,2,4-trichlorobenzene

## Conflicts of interest

The authors declare no conflict of interest.

## Supplementary Material
